# IRCAS: a novel end-to-end approach to identify, rectify, and classify comprehensive alternative splicing events in a transcriptome without genome reference

**DOI:** 10.1093/bib/bbag384

**Published:** 2026-07-21

**Authors:** Chenchen Shen, Quanbao Zhang, Qilong Cao, Xiaojun Liu, Zhen Zhang, Bailei Li, Zhenning Jin, Rongqing Zhang

**Affiliations:** Zhejiang Key Laboratory of Multiomics and Molecular Enzymology, Yangtze Delta Region Institute of Tsinghua University, Zhejiang, 705 Yatai Road, Jiaxing， Zhejiang, 314006, China; Department of Mathematics and Statistics, Northeastern University at Qinhuangdao, 143 Taishan Road, Qinhuangdao, Hebei, 066004, China; Zhejiang Key Laboratory of Multiomics and Molecular Enzymology, Yangtze Delta Region Institute of Tsinghua University, Zhejiang, 705 Yatai Road, Jiaxing， Zhejiang, 314006, China; Zhejiang Key Laboratory of Multiomics and Molecular Enzymology, Yangtze Delta Region Institute of Tsinghua University, Zhejiang, 705 Yatai Road, Jiaxing， Zhejiang, 314006, China; Zhejiang Key Laboratory of Multiomics and Molecular Enzymology, Yangtze Delta Region Institute of Tsinghua University, Zhejiang, 705 Yatai Road, Jiaxing， Zhejiang, 314006, China; Zhejiang Key Laboratory of Multiomics and Molecular Enzymology, Yangtze Delta Region Institute of Tsinghua University, Zhejiang, 705 Yatai Road, Jiaxing， Zhejiang, 314006, China; Zhejiang Key Laboratory of Multiomics and Molecular Enzymology, Yangtze Delta Region Institute of Tsinghua University, Zhejiang, 705 Yatai Road, Jiaxing， Zhejiang, 314006, China; Zhejiang Key Laboratory of Multiomics and Molecular Enzymology, Yangtze Delta Region Institute of Tsinghua University, Zhejiang, 705 Yatai Road, Jiaxing， Zhejiang, 314006, China; Zhejiang Key Laboratory of Multiomics and Molecular Enzymology, Yangtze Delta Region Institute of Tsinghua University, Zhejiang, 705 Yatai Road, Jiaxing， Zhejiang, 314006, China

**Keywords:** alternative splicing, graph neural networks, splice site rectification, genome reference-free, de Bruijn graph

## Abstract

Alternative splicing (AS) is a fundamental posttranscriptional mechanism that amplifies proteomic diversity and enables adaptive responses across eukaryotes. Current AS detection methods rely heavily on reference genomes, limiting their applicability to non-model organisms. Existing reference-free approaches suffer from inaccurate splice site prediction and treat detection and classification as separate processes, resulting in cascading errors. We present IRCAS, an integrated end-to-end framework for reference-free AS analysis, comprising three modules: identification, rectification, and classification. IRCAS employs colored de Bruijn graphs for AS detection, an attention-based convolutional neural network for splice site rectification, and a hybrid graph neural network combining graph attention network and Transformer layers for classification. Evaluation across four species demonstrates substantial improvements: splice site accuracy increased to 92%–96% versus 50%–55% for existing methods, and end-to-end inference accuracy reached 83.4% on rice (fine-tuned) compared to 44.7% for the previous best method. IRCAS establishes a new benchmark for reference-free AS detection in non-model organisms.

## Graphical Abstract

**Graphical Abstract:**
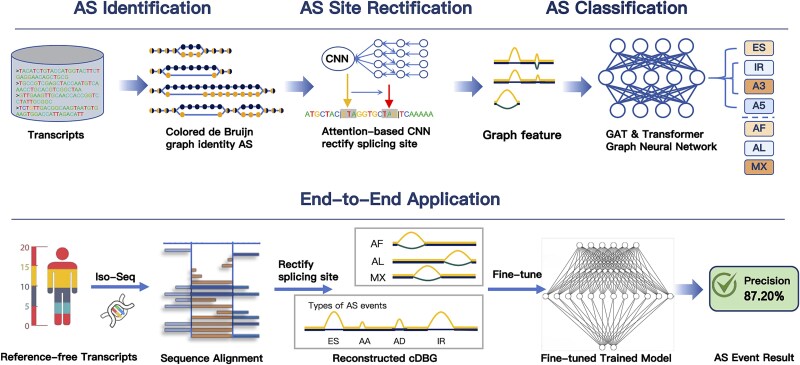

## Introduction

Alternative splicing (AS) is a posttranscriptional mechanism that increases proteomic diversity from a limited genome, contributing to phenotypic complexity and developmental plasticity in eukaryotes [1]. By enabling a single gene to generate multiple functionally distinct mRNA isoforms through differential AS events, this process fine-tunes cellular functions across tissues and developmental stages [2–4]. Critically, recent research demonstrates that AS is not merely a static feature but a dynamic modulator of environmental adaptation and disease response, including in host responses to viral infection [5–8]. The extensive regulatory capacity of AS establishes it as a pivotal evolutionary innovation, allowing organisms to enhance functional diversity without gene duplication and to swiftly adapt to selective pressures [9].

RNA-Seq [10] enhances AS investigation [11], while Iso-Seq [12] enables full-length transcriptome sequencing [13]. Tools like rMATS [14], SUPPA2 [15], and AStool [16] leverage these technologies to detect genome-wide AS events with corresponding genomic data. However, AS detection accuracy varies significantly, with over 70% discrepancy in differential splicing calls when using distantly related reference genomes [17, 18]. Additionally, many species lack corresponding genomic data, and acquiring such data is time-consuming and costly [19, 20]. These limitations underscore the need for reference-free AS detection methods to overcome the dependency on genomic data and improve applicability across diverse species.

Reference-free AS detection methods have evolved by improving how they identify splicing patterns in transcriptome data. Early approaches like Liu et al. [21] method utilized all-versus-all BLAST [22] to identify high-scoring segment pairs (HSPs) in order to detecting indels. These methods faced limitations in sensitivity for short splice variants, prompting AStrap [23] to integrate CD-HIT clustering with GMAP alignment [24], while IsoSplitter [25] leveraged SIM4-based alignments to detect insertion–deletion patterns characteristic of AS events, significantly improving AS detection performance. DeepASmRNA [26] enhanced the precision of AS event identification beyond AStrap by integrating biologically informed constraint. The paradigm shifted dramatically with MkcDBGAS [27], which introduced mixed k-mer colored de Bruijn graphs to dynamically identify topological “bubbles”—structures where shorter arm lengths (k-1) specifically indicate AS events. This approach achieved unprecedented precision by distinguishing paralogous genes in bubble topologies. These methodological leaps established the foundational principle that reference-free computational theory could replace reference genomes for accurate AS transcriptome pairing.

Subsequent research focused on accurate classification frameworks that transitioned from feature-dependent machine learning to end-to-end deep architectures. Seven canonical AS types are mechanistically defined: exon skipping (ES), alternative 3′ splice site (A3), alternative 5′ splice site (A5), intron retention (IR), alternative first exon (AF), alternative last exon (AL), and mutually exclusive exons (MX) [28]. AStrap pioneered AS typing with handcrafted features (splice site motifs, guanine-cytosine (GC) content) and tree-based classifiers, but struggled with unsatisfied precision. DeepASmRNA revolutionized this space through attention-based convolutional neural networks (CNNs) that directly processed one-hot encoded sequences around splice site, automatically learning discriminative patterns like “GT-AG” boundaries without manual feature engineering. The multi-scale CNN-Transformer hybrid in MCTASmRNA [29] further amplified this capability, where transformer captured long-range dependencies in sequence. Collectively, these advances represent feasible pathway toward integrated deep learning architectures capable of comprehensive AS detection and classification in reference-free contexts.

However, all previous methods have separated the prediction and classification of AS events, rather than providing an integrated inference workflow starting from the raw transcript sequence. In the prediction phase, past studies evaluated the accuracy of AS event prediction by comparing correctly paired transcripts against reference-based tools like SUPPA2. While this approach provides a general assessment of prediction accuracy, it fails to precisely pinpoint the exact locations of splicing events at the base-pair level. Although predictive algorithms incorporating constrained Blast data selection, such as DeepASmRNA and MCTASmRNA, achieve over 90% accuracy in identifying correct transcript pairs, these algorithms still exhibit an average error of 2.1 bp in predicting the actual splice sites compared to their true positions. MkcDBGAS optimizes splice site selection using mixed k-mer colored de Bruijn graphs (cDBGs), but it still exhibits an average position error of 1.3 bp in predicting splice sites. In the classification phase, past studies directly utilize data generated by reference-based tools that provide accurate splice sites and achieve relatively satisfied performance. In our ablation study, when using data with missing or incorrect splice sites, the model’s predictive performance significantly declined, with the previously best-performing model achieving only 74% accuracy. Critically, incorrect splice site predictions directly compromise downstream functional analysis, as even single nucleotide errors can misidentify exon-intron boundaries and lead to erroneous protein isoform annotations that fundamentally alter biological interpretations [30, 31]. In application scenarios, such positional inaccuracies not only reduce the reliability of AS event classifications but also propagate errors throughout comparative genomics studies, evolutionary analyses, and biomarker discovery workflows, resulting in a substantial loss of practical utility for researchers investigating alternative splicing mechanisms [32]. Moreover, due to the high diversity and species-specific nature of AS events, previous methods have generally failed to achieve satisfactory performance in transfer learning.

**Figure 1:**
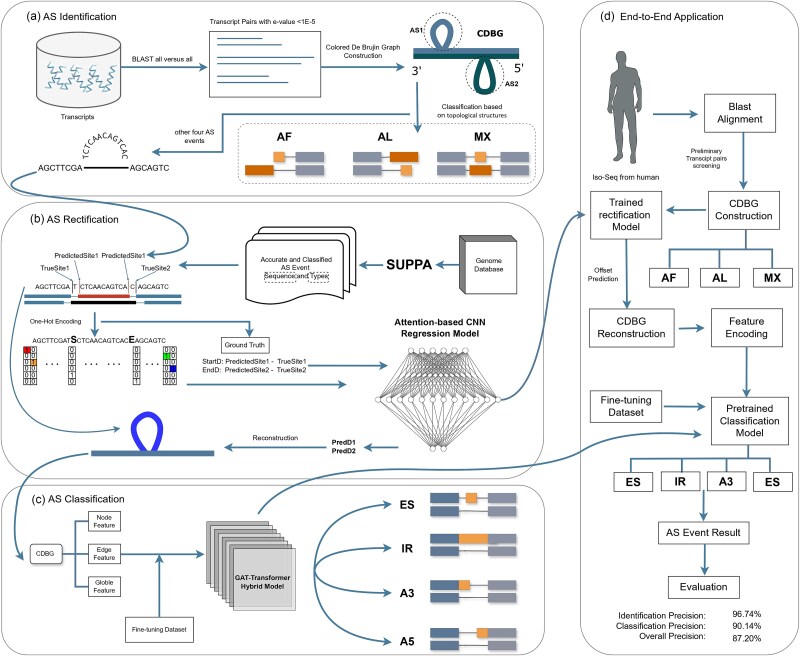
Workflow for construction and application of IRCAS. IRCAS is composed of three parts: identification, rectification, and classification. (a) Workflow for reference-free AS identification from a raw transcriptomic data. First, according to the input transcripts, we apply BLAST all versus all alignment for preliminary screen. Then, we adopt the MkcDBGAS graph construction strategy. A cDBG was constructed from two sequences using a specified k-mer size. Based on bubble topologies, bubbles were classified into five types: SNV-induced, four AS-induced, MX-induced, AL-induced, and AF-induced. (b) Workflow for AS position offset rectification and reconstruction of cDBG. Input transcript pairs are converted into a single sequence that includes two virtual nucleotides denoting the splicing start and end sites. SUPPA, a reference-based method, is utilized to determine the true splicing sites. The sequence is encoded into an n × 6 vector using one-hot encoding. The offset between the true and predicted splicing sites is calculated and encoded as the ground truth. An attention-based CNN rectification model is trained on these data to predict the offset, enabling the reconstruction of the cDBG with corrected splicing positions. (c) Workflow for four types of AS events shared same topology structure classification. For each cDBG, node features, edge features, and global features are extracted. These features are integrated into distinct layers of a GAT-Transformer hybrid model. This architecture enables high-precision classification of four types of AS events. (d) Workflow for end-to-end application of IRCAS. Transcriptomic data from any species lacking a reference genome is processed by IRCAS, enabling the classification of seven AS types with high accuracy.

In our study, we propose IRCAS, an end-to-end inference pipeline for AS event prediction and classification—one that, given a raw transcriptome, automatically produces classified AS events without manual intervention between modules, while internally consisting of three sequentially trained sub-modules each optimized for its specific sub-task (Fig. 1). For raw transcripts, we employ an initial screening process utilizing BLAST to identify potential transcript pairs, complemented by the construction of cDBG to detect topological signatures indicative of AS events [33]. To enhance the accuracy of splice site localization, we introduce an attention-based CNN regression rectification step, which refines predicted positions by leveraging contextual sequence information. Subsequently, a hybrid graph neural network (GNN) architecture is applied for the classification of AS event types, capitalizing on the refined positional data to achieve robust and accurate categorization. This integrated approach significantly surpasses the end-to-end accuracy of previous models, overcoming the challenges posed by inaccurate splice site predictions and enhancing the practical utility of AS detection and classification algorithms across diverse species and datasets. We evaluate IRCAS in two deployment modes: zero-shot inference on held-out species using only the pretrained base model, and fine-tuned inference using a small target-species labeled dataset. We report both modes separately throughout the manuscript.

## Materials and Methods

### Data acquisition and validation set construction

To ensure comprehensive and accurate training and evaluation of the AS event detection and classification models, we selected full-length transcript datasets encompassing both animal and plant species. Specifically, human and *Arabidopsis thaliana* datasets were utilized for training, while mouse and rice datasets were employed for evaluation. All datasets and their sample sizes used in this study are detailed in Table 1. Relevant data sources are available in Supplementary Table S1.

**Table 1:** All AS datasets and their sample sizes.

**AS types**	**Human**	***Arabidopsis thaliana***	**Rice**	**Mouse**
**IR**	7329	5992	2946	1762
**ES**	40 055	1051	788	11 945
**A5**	15 209	3161	1336	5885
**A3**	16 727	4211	2472	7294
**AF**	86 273	936	235	6119
**AL**	18 923	106	61	3398
**MX**	5591	31	18	1526
**Total**	190 107	15 488	7856	37 924

To train and test the IRCAS model, we applied the “generateEvents” function in SUPPA2 with default settings to human and *A. thaliana* annotation files, leveraging SUPPA2’s capability for accurate, systematic, and efficient genome-wide detection of all precise AS event positions and seven AS event types, which served as ground truth and labels.

### Comparing existed identification methods

To identify splice sites and clarify transcript pairs, the DeepASmRNA employed BLAST for pairwise alignment of transcript sequences with imposed constraints to detect potential splice sites. Alternatively, MkcDBGAS employs a cDBG algorithm to investigate AS events through their topological structures. Although both methods demonstrated satisfactory performance in identifying transcript pairs, they did not prioritize the accuracy of AS position detection.

### cDBG construction and AF, AL, MX classification

We employed MkcDBGAS’ workflow to identify potential alternatively spliced transcript pairs and roughly provide splicing positions. Transcript sequences were first screened for similarity using BLAST with an e-value threshold of 1e-10. For each qualifying pair, the minimum k-mer length (k) was determined as the shortest non-repetitive sequence segment to ensure an acyclic cDBG. The cDBG was then constructed as G = (V, E, C) where V comprises nodes representing k-mers from the transcripts, E includes directed edges connecting nodes with overlapping (k-1)-mers, and C denotes color labels indicating transcript origins. Bubbles [34], representing variant regions, were identified and classified topologically into single nucleotide variant (SNV)-induced (arm lengths = k), four-AS-induced (shorter arm = k-1), or other-induced (both arms > k). Three types of AS events were detected by traversing G’ to identify other-induced bubbles, applying specific criteria: a single other-induced bubble with the shorter arm <30% of the transcript length, positioned at the start (AF), end (AL), or middle (MX). For other AS types, one or more four-AS-induced bubbles with fewer than three SNV-induced bubbles were identified. The four-AS-induced category encompasses the remaining four AS event types (ES, IR, A3, and A5), which exhibit identical topological structures.

### Computational complexity

The identification module consists of two stages: an all-vs-all BLAST pre-screen and per-pair cDBG construction for the survivors. For N input transcripts of average length L-, the BLAST screen has worst-case complexity O(N^2^ · L-) but is substantially faster in practice due to BLAST’s k-mer seeding heuristic and the sparsity induced by the stringent e-value threshold (1 × 10^−10^). Let M denote the number of transcript pairs surviving this filter. Biologically, M depends on gene-family structure but is in practice much smaller than the theoretical maximum N(N − 1)/2, because most cross-gene pairs lack significant homology under the stringent cutoff. For each surviving pair, cDBG construction uses rolling-hash k-mer extraction and graph traversal at cost O(L_max), giving an aggregate identification cost of O(BLAST_screen + M · L_max). The downstream rectification and classification stages also operate only on surviving pairs and contribute additional O(M · L_max) terms with small constants. Empirical pair counts on the four species evaluated in this study show M roughly linear in N (specifically, M / N ≈ k with k a small constant on the order of 10^1^ for our datasets).

### AS rectification overview

Given the substantial splicing position errors inherent in previous AS identification algorithms, we propose an attention-based CNN [35] rectification model to address these inaccuracies. The model integrates multi-scale convolutional feature extraction with self-attention mechanisms to capture both local sequence patterns and long-range dependencies critical for splice site recognition, and outputs rectified positions with high accuracy.

### Rectification train and test set construction and data preprocessing

We used the positional offsets between cDBG-predicted splice sites and the corresponding true splice sites (determined by SUPPA2 based on the reference genome) as the ground truth for model training. Since offsets exceeding 50 bp are excessively large and extend beyond the supplemented upstream and downstream sequence boundaries, we excluded these samples during the rectification process.

The rectification model processes transcript sequences through a systematic encoding scheme that transforms nucleotide sequences into numerical representations suitable for deep learning. To address class imbalance and improve model generalization, samples with large splice site deviations (|offset| > 3) are replicated twice. Each sequence is composed of three distinct regions: upstream sequences (50 bp), AS region (padded to uniform dimension), and downstream sequences(50 bp). Virtual nucleotides are inserted between these regions to delineate splice boundaries: “S” represents the splice start site and “E” represents the splice end site. The encoding strategy employs a six-dimensional one-hot encoding for each position.

### Convolutional feature extraction and self-attention mechanism

The rectification model employs a hierarchical convolutional architecture with progressively increasing filter complexity, consisting of four convolutional blocks: 64 filters with 3 × 1 kernels for local pattern detection, 128 filters with 5 × 1 kernels for medium-range motif recognition, 256 filters with 7 × 1 kernels for extended sequence context, and 512 filters with 3 × 1 kernels for high-level feature abstraction. Each convolutional layer is followed by batch normalization, ReLU activation, and strategic max-pooling operations (stride = 2) to reduce dimensionality while preserving critical features, with dropout regularization (rate = 0.1) applied to prevent overfitting. Following convolutional feature extraction, the model incorporates a multi-head self-attention module to capture long-range dependencies between distant sequence positions, comprising 8 attention heads with 512-dimensional embeddings, layer normalization applied before attention computation for training stability, and residual connections through the attention mechanism design. The attention module enables the model to dynamically focus on relevant sequence regions regardless of their positional distance, which is crucial for accurate splice site boundary detection, effectively combining the local pattern recognition capabilities of CNNs with the global context modeling of attention mechanisms.

### Huber loss

To address the imbalanced offset distribution centered around zero, we employed Huber loss [36] to ensure robust optimization and stable convergence during training.


(1) \begin{equation*} {L}_{\delta}\left(y,f(x)\right)=\left\{\begin{array}{l}\frac{1}{2}{\left(y-f(x)\right)}^2,\kern0.5em \mathrm{if}\ \left|y-f(x)\right|\le \delta \\{}\delta \left|y-f(x)\right|-\frac{1}{2}{\delta}^2,\kern0.5em \mathrm{if}\ \left|y-f(x)\right|>\delta \end{array}\right. \end{equation*}

The dual-regime mechanism enables precise learning of dominant near-zero offsets through a quadratic penalty for small errors (|offset| ≤ δ) and bounded gradients for rare large offsets through a linear penalty above δ. We selected the threshold parameter δ by examining the empirical distribution of positional offsets on the validation set during early training, setting δ at the 90th percentile of this distribution. Under this choice, approximately 90% of validation samples fall within the quadratic-penalty regime, preserving fine-grained gradients for the dominant near-zero offsets, while the remaining 10%, predominantly outliers from false-positive transcript pairings, fall within the linear-penalty regime, where the bounded gradient stabilizes training and prevents extreme positional noise from disrupting convergence on the majority. The resulting value (δ = 3.6 on *Arabidopsis* dataset) and the validation error distribution motivating this choice are shown in Supplementary Fig. S1.

### Reconstruction of AS events after rectification

Using the predicted offset from the rectification model, we precisely relocate splicing sites and align the upstream and downstream sequences, generating an accurate dataset for following AS classification and subsequent analyses

### AS classification model architecture overview

**Figure 2:**
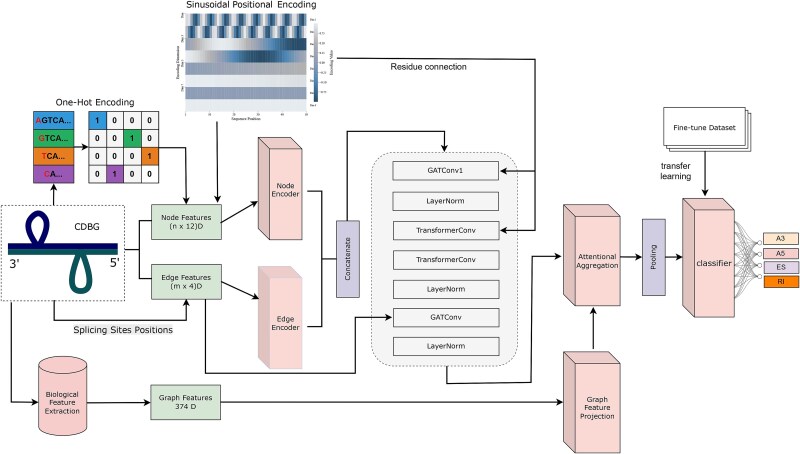
The classification model architecture. (a) Backbone: encodes sequence, structural, and biological features into node, edge, and graph representations. (b) Neck: graph neural network layers with residual connections and attentional aggregation to extract high-level graph embeddings. (c) Head: applies transfer learning to predict AS event types.

To accurately classify AS events into distinct categories, we propose a hybrid GNN architecture that combines graph attention networks (GATs) and Transformer [37] convolution layers. The model processes cDBG graphs representing AS events, where nodes encode sequence features and edges capture splicing structure. The architecture integrates attentional aggregation mechanisms to extract discriminative graph-level representations, enabling robust classification across four AS event types despite significant class imbalance (Fig. 2).

### Two-level classification design

Classification of the seven SUPPA2 AS categories is performed at two levels in IRCAS, reflecting their distinctive cDBG topologies. Three categories, MX, AF, and AL, produce structurally distinctive cDBG patterns: MX events form multi-bubble structures, while AF and AL events form open-ended structures at the transcript boundaries. These categories are identified directly from the cDBG topology by structural inspection, without requiring a learned classifier. The remaining four categories, A3, A5, ES, and IR, all produce single-bubble topologies that are structurally similar to one another and are distinguishable only through joint analysis of graph topology and underlying sequence. These four categories are classified by the GAT-Transformer hybrid module described above.

### Classification train set construction and feature extraction

The training and test dataset comprises cDBG graph representations of AS events, with each graph labeled according to its AS type based on the result of SUPPA. Each graph contains three different levels of features: node, edge, and graph. Node features represent k-mers from the RNA sequence, encoded with a 4-dimensional one-hot vector based on the last nucleotide and an 8-dimensional sinusoidal positional encoding (PE) [38], forming a 12-dimensional feature vector.


$$ P{E}_{\left( pos,2i\right)}=\sin \left(\frac{pos}{10000^{2i/{d}_{\mathrm{model}}}}\right) $$

(2) \begin{equation*} P{E}_{\left( pos,2i+1\right)}=\cos \left(\frac{pos}{10000^{2i/{d}_{\mathrm{model}}}}\right), \end{equation*}

$$ w\mathrm{h} ere\ i\in \left[0,\frac{d_{\mathrm{model}}}{2}\right) $$

Edge features capture connections between k-mers, encoded as a 4-dimensional vector with two binary indicators for out-degree and in-degree indicating the splicing site and a 2-dimensional sinusoidal positional encoding of relative positions. Graph-level features (Supplementary Tables S14 and S15), derived from the sequences based on validated biological features [39, 40], represented as a tensor to capture global biological properties of each AS event.

### Hybrid graph neural network architecture

The classification model employs a hierarchical architecture to process cDBG graph representations for AS event classification, integrating node, edge, and graph-level features. Node features are processed through a dual-layer encoder with linear transformations, layer normalization, GELU activation, and dropout for robust feature extraction. Edge features are transformed via a dedicated edge encoder. Initially, a GAT [41] layer with multiple attention heads models local neighborhood interactions, followed by a projection layer. Subsequently, two Transformer layers employ edge-aware attention to capture long-range dependencies across the graph. A final single-head GAT layer refines local patterns. Each layer incorporates layer normalization, GELU activation [42], dropout, and residual connections to enhance training stability and performance. Node embeddings are augmented with positional encoding through a scaled residual connection. Graph-level representations are derived through attentional aggregation and global mean pooling, refined by a multi-head self-attention mechanism. Graph-level features are processed through a multilayer encoder and concatenated with pooled node features. The combined representation undergoes feature fusion through a multilayer network with linear transformations, layer normalization, GELU activation, and dropout, followed by a linear classifier producing logits for classification.

### Hybrid loss function addressing data imbalance

To address the class imbalance prominent in the AS events dataset—where categories with fewer samples are critical—we developed a novel hybrid loss function during training. This combined cross-entropy loss [43], focal loss [44], and center loss [45] to simultaneously mitigate imbalance issues and reduce intra-class feature distances. We further enhanced this approach with dynamic weight adaptation, boosting the model’s responsiveness to all categories and overall classification accuracy.

Cross-entropy loss: served as the main loss function, provide stable loss for classification.


(3) \begin{equation*} {\mathrm{L}}_{\mathrm{c}\mathrm{e}}=-\frac{1}{\mathrm{N}}{\sum}_{\mathrm{i}=1}^{\mathrm{N}}{\sum}_{\mathrm{c}=1}^4{\mathrm{y}}_{\mathrm{i},\mathrm{c}}\log \left(\hat{{\mathrm{y}}_{\mathrm{i},\mathrm{c}}}\right) \end{equation*}

where $N$ denotes the total number of samples in the dataset. The variable $C$ the class. For the $i$ sample, ${y}_{i,c}$ is the ground-truth label. The term $\hat{{\mathrm{y}}_{\mathrm{i},\mathrm{c}}}$ is the predicted probability that the $i$ sample belongs to class $C$.

Focal loss: addresses class imbalance through effective number weighting and focuses learning on hard-to-classify samples.


(4) \begin{equation*} {L}_{\mathrm{focal}}=-{\mathrm{\alpha}}_t{\left(1-{p}_t\right)}^{\mathrm{\gamma}}\log \left({p}_t\right) \end{equation*}

where ${\alpha}_t=\frac{1-\beta }{1-{\beta}^{n_t}}$ class-specific weights computed from sample frequencies ${\mathrm{n}}_{\mathrm{t}}$ with smoothing parameter $\mathrm{\beta}$ closing to 1, and γ controls the focusing effect on misclassified examples. Additional bias factor is applied to minority class to compensate for its severe underrepresentation.

Center loss: enhances intra-class feature compactness by minimizing feature dispersion around class centers.


(5) \begin{equation*} {L}_{\mathrm{center}}=\frac{1}{2N}{\sum}_{i=1}^N{\left|{f}_i-{c}_{y_i}\right|}_2^2 \end{equation*}

where ${f}_i$ denotes the feature embedding, ${c}_{y_i}$ represents the learnable class center for label ${y}_{i.}$

The hybrid loss integrates these components with adaptive normalization:


(6) \begin{equation*} {L}_{\mathrm{total}}=\alpha \cdotp \widehat{L_{\mathrm{focal}}}+\beta \cdotp \widehat{L_{\mathrm{ce}}}+\gamma \cdotp \widehat{L_{\mathrm{ce}\mathrm{nter}}} \end{equation*}

where α, β, and γ are weight hyperparameters constrained to α + β + γ = 1, governing the relative contributions of focal loss, cross-entropy loss, and center loss, respectively. We set α = 0.7, β = 0.29, γ = 0.01 on plant model, for example, selected by evaluating several candidate combinations on the validation set and retaining the best-performing setting. The ordering α > β > > γ reflects the distinct role of each component: focal loss (α) bears the dominant weight because it directly addresses the class imbalance that is the central difficulty in AS classification; cross-entropy loss (β) provides stable gradients for the majority classes and is comparable to but smaller than α; center loss (γ) serves as a feature-level regularizer compressing intra-class variance and must remain small relative to the discriminative losses to avoid collapsing the inter-class decision boundaries.

### Evaluation and comparison to state-of-the-art model

To objectively evaluate IRCAS’s classification performance, we employed multiple metrics, including precision, recall, accuracy, and F1-score, and benchmarked IRCAS against four state-of-the-art reference-free AS detection approaches: AStrap, DeepASmRNA, MkcDBGAS, and MCTASmRNA. To ensure a fair comparison, all baseline methods were retrained from scratch on our standardized dataset, with the exception of MCTASmRNA, whose training code is not publicly available; for MCTASmRNA, we evaluated the released pretrained classifier on our dataset, composed with the upstream identification and splice-site localization pipeline of DeepASmRNA to provide complete end-to-end predictions.

### Cross-validation and statistical evaluation

All methods were evaluated under a five-fold gene-disjoint cross-validation protocol, in which transcript pairs were partitioned by gene identity (using GroupKFold on gene IDs [46] so that no gene appeared in both training and evaluation folds within any single fold split. This protocol prevents the gene-level information leakage that can arise from sample-level random splits. Each (method, fold) combination was repeated with 3 distinct training seeds, yielding 15 independent runs per (species, method) pair. Performance is reported throughout the manuscript as mean ± standard deviation across these 15 runs.

### Statistical significance testing

Because IRCAS and all baseline methods share an identical 5-fold × 3-seed grid, the 15 per-run accuracy differences between IRCAS and each baseline are naturally paired. We tested the null hypothesis of equal per-run performance using the two-sided paired Wilcoxon signed-rank test, with multiple-comparison correction across the four baseline comparisons within each species via the Holm–Bonferroni procedure.

### Comparison on the MCTASmRNA dataset

To address concerns that the use of MCTASmRNA’s pretrained model on our dataset might disadvantage MCTASmRNA, we additionally re-evaluated all methods on the original MCTASmRNA dataset under five-fold cross-validation.

### Fine-tune dataset and transfer learning

Due to the significant variation in the characteristics and distribution of AS events across species, we employed a transfer learning strategy with a fine-tuned dataset to enhance the model’s generalization and accuracy. This approach utilized a small labeled dataset to adjust critical parameter in order to improve classification performance. In our study, we set the models trained on Human and Araport as the base model for animal and plant. For each pretrained model, only the head layer was trained, with all other layer parameters kept frozen. To validate the significance of transfer learning and assess model generalizability, we conducted an ablation study comparing models with different dataset scales and without fine-tuning on the same dataset.

## Results

We introduce IRCAS, a novel tool for predicting AS events from full-length transcripts without reliance on a reference genome. IRCAS accepts full-length transcripts as input and generates two outputs: (i) AS transcript pairs with high credited positions of AS events, (ii) AS event types along with their confidence scores.

### Alleviating offset inaccuracy through rectification model

Previous methods for identifying AS, such as DeepASmRNA and MkcDBGAS, primarily focused on detecting transcript pairs, often neglecting precise verification of AS event positions. In the *Arabidopsis* dataset, these methods achieved exact AS-position accuracy of only 50.1% and 53.8%, respectively. Analysis of cDBG identification splice-site offsets (Table 2) revealed that approximately 56%–58% of predicted start and end positions were exactly accurate, with most inaccuracies concentrated within a small range (33%–39% within ±2 bp) and a minor fraction (5%–6%) deviating further (up to ±10 bp or more), indicating moderate positional imprecision in prior approaches.

**Table 2:** cDBG identification start position and end position offset in *Arabidopsis* dataset.

**Interval**	**Start site offset (count)**	**Start site offset (Percent)**	**End site offset (count)**	**End site offset (Percent)**
**(−∞, −10)**	0	0.00%	1	0.00%
**[−10, −2)**	0	0.00%	0	0.00%
**[−2, 0)**	850	4.40%	0	0.00%
**[0]**	11 075	57.50%	10 740	55.80%
**(0, 2 ]**	6348	33.00%	7443	38.60%
**(2, 10 ]**	929	4.80%	1017	5.30%
**(10, ∞)**	59	0.30%	60	0.30%

These residual offsets are a systematic consequence of how the two upstream identification paradigms locate splice junctions, rather than random noise. BLAST-based identification uses high-scoring segment pair (HSP) scoring with a similarity threshold: when two transcripts diverge gradually around a splice junction, the HSP boundary falls on whichever side the cumulative similarity score first crosses threshold, producing systematic offsets typically within ±5 bp and biased toward whichever flank carries higher local similarity. cDBG-based identification uses bubble structures in compacted de Bruijn graphs: when the sequence near a splice junction contains a low-complexity tract such as a homopolymer run (e.g. AAAA…), the bubble’s k-mer endpoints shift by several base pairs in the direction of the repeat, because adjacent k-mers become indistinguishable within the repeat region. In both cases, the residual error is small, continuous, and sequence-context dependent, properties that motivate the regression-based rectification step described in the next section.

**Figure 3:**
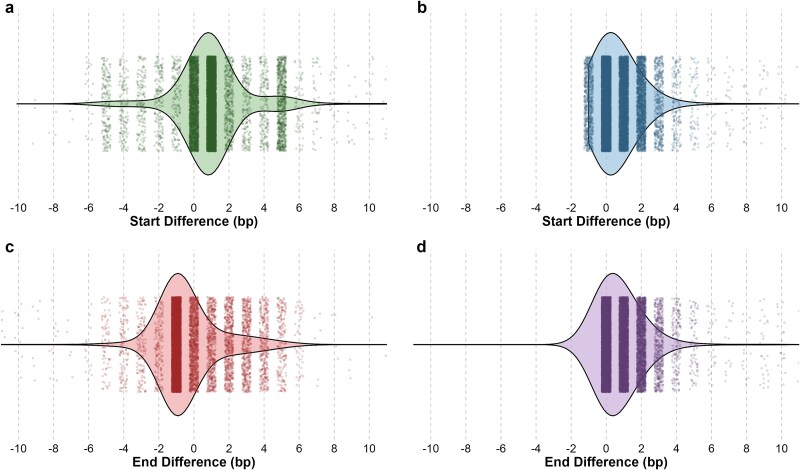
AS position errors distribution by violin plot superimposed with jittered points. (a) Splicing start position error of BLAST. (b) Splicing end position error of BLAST. (c) Splicing start position error of cDBG. (d) Splicing end position error of cDBG.

Figure 3 shows violin plots of the AS-position identification offsets (predicted minus true coordinate) for both methods. The cDBG approach produces a distribution that is more tightly concentrated around zero than BLAST, with narrower tails on both sides, consistent with the mechanistic difference described above, although a small residual offset distribution remains, which the rectification module is designed to remove.

To evaluate the performance of our rectification models in accurately localizing AS sites across diverse species, we developed two independent models tailored to the distinct transcriptome organizations of animals and plants. The animal-trained model was developed using human data to capture splicing patterns characteristic of animal species, while the plant-trained model was trained on *A. thaliana* data to account for plant-specific features. These models were designed to address lineage-specific sequence characteristics and enhance splice site prediction accuracy.

**Figure 4:**
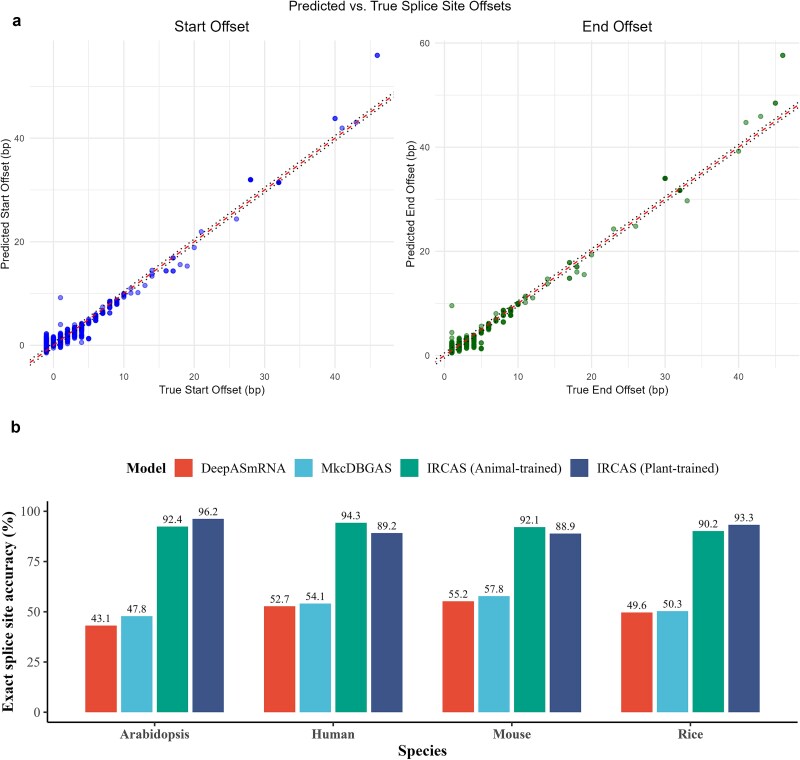
(a) Scatter plots of predicted versus true splice-site offsets (bp) for the *Arabidopsis* dataset, with the dashed diagonal line indicating the ideal 1:1 correlation. (b) Comparison of exact splice-site accuracy (%) across species (human, mouse, *Arabidopsis*, rice) for DeepASmRNA, MkcDBGAS, and IRCAS (animal-trained and plant-trained).

We assessed the performance of three tools: DeepASmRNA, MkcDBGAS, and our proposed IRCAS (with separate animal- and plant-trained models) across four species: human, mouse, *Arabidopsis*, and rice. The exact splice site accuracy (%) was measured for each model, with results summarized in a bar chart (Fig. 4). The animal-trained IRCAS model achieved mean accuracies of 94.3% for human and 92.1% for mouse, significantly outperforming DeepASmRNA (52.7% for human, 55.2% for mouse) and MkcDBGAS (54.1% for human, 57.8% for mouse). Similarly, the plant-trained IRCAS model demonstrated superior performance in plant species, with mean accuracies of 96.2% for *Arabidopsis* and 93.3% for rice, compared to DeepASmRNA (43.1% for *Arabidopsis*, 49.6% for rice) and MkcDBGAS (47.8% for *Arabidopsis*, 50.3% for rice).

The rectification step in IRCAS substantially improved splice site localization across all tested species. Compared to baseline methods, the proportion of exactly aligned splice sites increased by 34%–43% across human, mouse, *Arabidopsis*, and rice, highlighting the robustness of our attention-based CNN rectifier. Moreover, the training curve showcases the loss decreasing significantly, indicating the effectiveness of Huber loss (Supplementary Fig. S2). These improvements confirm that the rectification strategy effectively generalizes beyond the training organism, accommodating species-specific splicing patterns. Collectively, these results demonstrate that our species-specific training approach, combined with the rectification methodology, provides a robust and scalable framework for accurate AS event reconstruction across diverse species (Fig. 4).

To verify that the rectification model performs consistently across AS categories of varying structural complexity, we further stratified rectification accuracy by SUPPA2 category (Supplementary Table S2 and Supplementary Method S2). The model maintained high performance across all seven categories, with an overall weighted accuracy of 96.2% on *Arabidopsis*. The highest accuracies were observed for A3 (98.0%) and ES (97.2%), reflecting their well-defined exonic consensus motifs at clearly separated splice sites; the structurally more complex IR (94.8%) and MX (92.2%) categories, where intronic boundaries and coordinated dual-junction selection are intrinsically harder to localize, yielded somewhat lower but still consistent performance. This uniformity across categories confirms that rectification is not specialized to a subset of canonical AS events.

### Topology-based classification of structurally distinctive AS categories

For the three SUPPA2 categories whose cDBG topologies are structurally distinctive, AF, AL, and MX, direct topological inspection achieved high identification accuracy on both training species (Supplementary Table S3 and Supplementary Method S3). Precision exceeded 93% for all three categories on both human and *Arabidopsis* (AF: 95.3%/97.1%; AL: 94.4%/94.5%; MX: 93.4%/94.5%), confirming that the multi-bubble (MX) and open-ended (AF, AL) topologies are unambiguously distinguishable from cDBG structure alone. The four single-bubble categories (A3, A5, ES, and IR), which share structurally similar cDBG patterns, are instead classified by the GAT-Transformer module evaluated below.

### A robust and highly accurate landscape of AS classification

To evaluate the effectiveness of ICRAS for AS event classification, we conducted comprehensive performance assessments across four species datasets and compared IRCAS against state-of-the-art methods. Our evaluation encompassed both individual class performance and overall classification accuracy, with particular attention to the challenging class imbalance inherent in AS event datasets.

**Figure 5:**
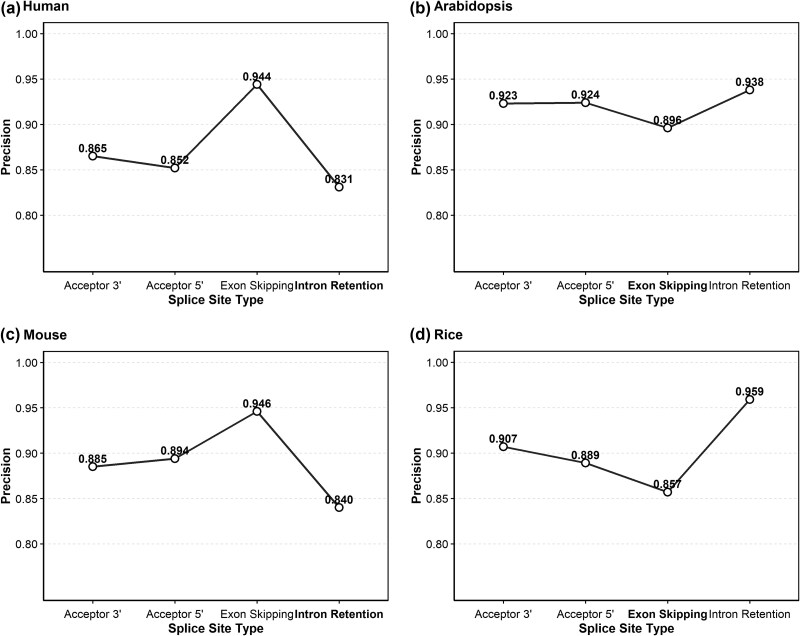
Precision of splice-site prediction across four species: (a) human, (b) *Arabidopsis*, (c) mouse, and (d) rice. Bars indicate precision values for four event types (A3, A5, ES, and IR), with the least frequent splicing event type highlighted in bold on the *x*-axis.

IRCAS demonstrated superior classification performance across all tested species, achieving consistently high accuracy, precision, recall, and F1-scores (Fig. 5, Table 3, Supplementary Fig. S3). The overall classification accuracies were: human (90.1%), mouse (91.1%), *Arabidopsis* (92.8%), and rice (91.8%). These results represent substantial improvements over baseline methods, with particularly notable enhancements in precision and recall balance across all AS event types. The hybrid loss function effectively addressed class imbalance issues, as evidenced by the balanced performance across different AS types. Species-specific analysis revealed distinct performance patterns across taxonomic lineages. For animal species, the animal-trained model based on human dataset achieved excellent performance across both human and mouse datasets. For the human dataset, precision ranged from 83.1% (IR) to 94.4% (ES). The mouse dataset showed similar patterns, with precision values from 84.0% (IR) to 94.6% (ES). The consistent high performance across both species validates the generalizability of our animal-specific training approach. For plant species, the plant-trained model based on *Arabidopsis* dataset exhibited even stronger performance on plant datasets. *Arabidopsis* achieved the highest overall accuracy (92.8%), with precision values ranging from 89.6 (ES) to 93.8 (IR). The performance on rice datasets was similarly robust, exhibiting comparable trends, with precision ranging from 85.7% (ES) to 95.9% (IR). The superior performance on plant datasets may reflect the more conserved splicing patterns in plant transcriptomes compared to the complexity of animal AS. Additionally, IRCAS effectively addressed minority class samples, achieving acceptable precision for ES events in plants and RI events in animals.

**Table 3:** Prediction performance for four species evaluated using overall accuracy, recall, F1-score, and precision for A3, A5, ES, and IR splicing events.

**Dataset**	**Accuracy**	**Recall**	**F1**	**Precision**			
				**A3**	**A5**	**ES**	**IR**
**Human**	0.901	0.899	0.900	0.865	0.852	0.944	0.831
**Ara**	0.928	0.887	0.907	0.923	0.924	0.896	0.938
**Mouse**	0.911	0.873	0.892	0.885	0.894	0.946	0.840
**Rice**	0.918	0.903	0.910	0.907	0.889	0.857	0.959

### Classification performance comparing to state-of-the-art model

**Figure 6:**
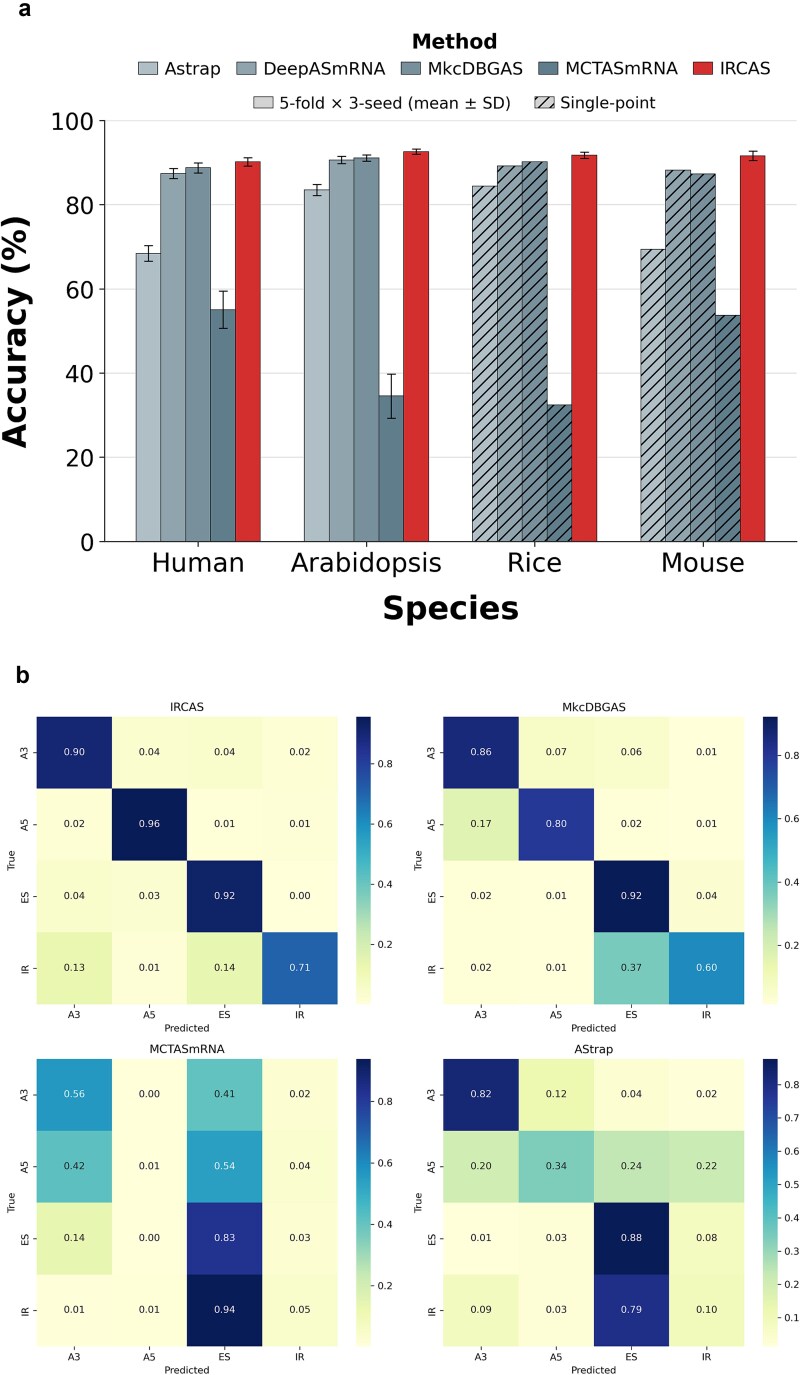
(a) Classification accuracy comparison of five AS-event detection methods (AStrap, DeepASmRNA, MkcDBGAS, MCTASmRNA, and IRCAS) across human, *Arabidopsis*, rice, and mouse datasets. (b) Normalized confusion-matrix heatmaps of AS-event classification on the human dataset across four methods (IRCAS, MkcDBGAS, MCTASmRNA, and AStrap).

IRCAS was benchmarked against four established AS classification methods across four species (Table 4, Fig. 6). Under a 5-fold gene-disjoint cross-validation protocol with 3 training seeds per fold (15 runs per species, see Methods), IRCAS demonstrated superior classification accuracy across all tested species, with improvements of 1.5–4.3 percentage points over the previous best-performing method, MkcDBGAS. Paired two-sided Wilcoxon signed-rank tests with Holm–Bonferroni correction confirmed that IRCAS significantly outperforms every baseline on the human and *Arabidopsis* datasets (Holm-adjusted *P* ≤ 2 × 10^−3^ for all comparisons; Table 4 and Supplementary Tables S4–S12); the per-run sign pattern (IRCAS – baseline) was 15/0/0 in every comparison, indicating that IRCAS wins on every fold and every seed without exception.

**Table 4:** Overall classification accuracy (%) comparison across methods and species, with Holm–Bonferroni correction across the four baselines (^**^P < .01; ^***^P < .001).

**Method**	**Human**	***Arabidopsis***	**Rice**	**Mouse**
**Astrap**	68.43 ± 1.88^**^	83.49 ± 1.35^**^	84.4	69.4
**DeepASmRNA**	87.41 ± 1.17^**^	90.63 ± 0.87^**^	89.2	88.2
**MkcDBGAS**	88.75 ± 1.20^**^	91.06 ± 0.75^**^	90.2	87.3
**MCTASmRNA**	55.05 ± 4.41^***^	34.52 ± 5.26^***^	32.4	53.7
**IRCAS**	90.20 ± 0.97	92.61 ± 0.65	91.75 ± 0.77	91.61 ± 1.13

DeepASmRNA and MkcDBGAS showed the strongest baseline performance on the human and *Arabidopsis* datasets (87.4%–91.1% classification accuracy across species), while AStrap lagged significantly (68.4%–84.4%), reflecting the limitations of traditional feature-based approaches compared to deep learning methods. MCTASmRNA displayed considerably lower performance (32.4%–55.3% across datasets) on our standardized dataset.

To verify that this gap reflects genuine generalization differences rather than dataset bias against MCTASmRNA, we additionally re-evaluated all methods on the original MCTASmRNA dataset under five-fold cross-validation (Supplementary Fig. S4). On that dataset, every method including MCTASmRNA itself achieved substantially higher accuracy (86.7%–97.96%) than on our standardized dataset (68.4%–90.2%), consistent with the MCTASmRNA dataset having undergone extensive preprocessing prior to release, a property that benefits every model evaluated on it. Critically, IRCAS retained the highest accuracy (97.96%) on the MCTASmRNA dataset as well, indicating that its margin is not dataset-specific and reflects algorithmic differences rather than dataset-tuning advantages.

The normalized confusion matrices heatmaps (Fig. 6b) further illustrate the classification patterns of the four methods. IRCAS achieved balanced and high precision across all AS event types, with relatively few off-diagonal misclassifications. In contrast, MkcDBGAS maintained competitive accuracy but exhibited higher confusion of minority class. AStrap showed pronounced misclassifications, particularly for ES and IR events, highlighting its limited generalizability. MCTASmRNA exhibited the poorest performance, displaying widespread misclassification across categories, indicative of its instability and bias toward the majority class.

### Ablation study

**Figure 7:**
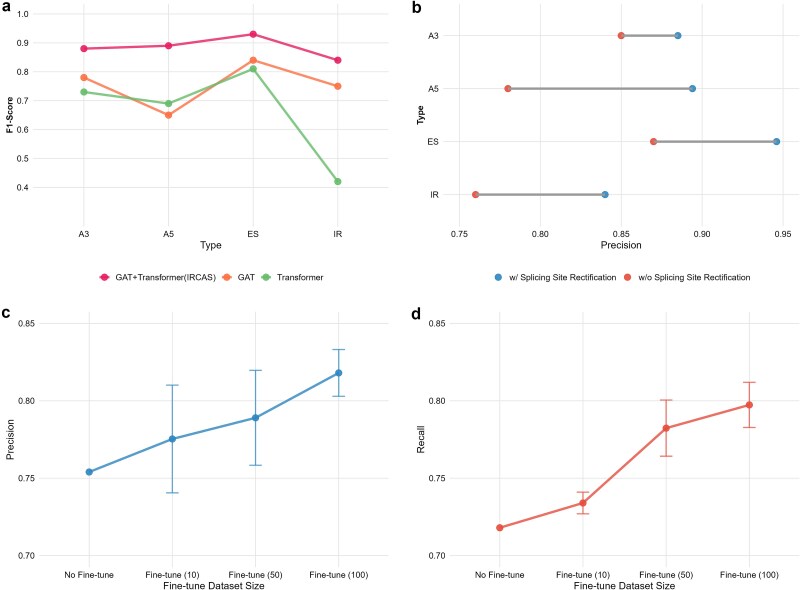
Ablation studies of IRCAS components based on the mouse dataset. (a) F1-scores comparing the GAT+Transformer hybrid against individual GAT and Transformer architectures. (b) Precision improvement with splicing-site rectification. (c) Fine-tuning performance across dataset sizes (10, 50, 100 samples) for precision. (d) Fine-tuning performance across dataset sizes (10, 50, 100 samples) for recall. Error bars represent standard deviation from three independent random stratified sampling experiments.

To comprehensively assess the contribution of individual components within IRCAS, we conducted systematic ablation studies examining the GNN components, the rectification module, and the fine-tuning strategy across multiple performance dimensions (Fig. 7).

To validate the effectiveness of our hybrid architecture, we compared the performance of individual components against the complete IRCAS model (Fig. 7a). The GAT+Transformer (IRCAS) hybrid architecture consistently outperformed individual components across all AS event types. Compared to standalone Transformer implementation, standalone GAT implementation presents better performance, especially in minority class (IR). These results demonstrate that the local graph relationships near the AS region captured by GAT are of paramount importance, while the Transformer’s modeling of long-range sequential patterns also significantly contributes to effective classification. The hybrid architecture successfully integrates these complementary strengths, with GAT providing structural understanding and Transformer capturing sequential dependencies that neither component adequately addresses alone.

We evaluated the impact of the splicing site rectification module in classification by comparing model performance with and without this component (Fig. 7b). The rectification module demonstrated consistent improvements across all four AS event types. For A3 events, precision increased from 0.85 to 0.885. A5 events showed the most substantial improvement, rising from 0.78 to 0.894. ES events improved from 0.76 to 0.84, while IR events increased from 0.87 to 0.946. These results demonstrate that the rectification module provides crucial position refinement that significantly enhances classification accuracy across all AS types. The pronounced improvements in detecting these splice variants suggest that they are particularly sensitive to positional accuracy, likely due to subtle sequence differences that distinctly alter the features of the AS region.

To assess the relationship between fine-tuning strategy and model performance, we conducted experiments on mouse dataset using different numbers of fine-tuning samples: 10, 50, and 100 samples (Fig. 7c and d). For precision metrics, the baseline model trained on human dataset without fine-tuning achieved 0.754. Fine-tuning with 10 samples improved performance to 0.775, representing a 2.1 percentage point increase. Scaling to 50 samples yielded further improvements to 0.789 while 100 samples achieved the highest precision of 0.818. The diminishing marginal returns observed between 50 and 100 samples suggest that substantial performance gains can be achieved with relatively small fine-tuning datasets. Similar trends were observed for recall metrics, with baseline performance at 0.718 improving to 0.734 with 10 samples, 0.782 with 50 samples, and reaching 0.797 with 100 samples. The consistent reduction in error bars with increasing sample size indicates improved model stability and generalization. Crucially, the selection of the fine-tuning dataset is pivotal to the model’s effectiveness, as non-representative datasets may compromise performance. Notably, the most substantial performance gains were observed between the baseline and 10-sample fine-tuning conditions, suggesting that even minimal fine-tuning yields significant benefits for cross-species generalization.

### Applying IRCAS to non-trained datasets

To assess IRCAS’s cross-species generalizability, the practical setting in which a researcher lacks reference data for the target species, we evaluated end-to-end performance on mouse using models trained exclusively on human data, and on rice using models trained on *Arabidopsis*. Table 5 reports per-stage accuracies (transcript pair identification, splice-site localization, AS-type classification) and end-to-end accuracy measured directly on the full pipeline output, allowing both stage-level diagnosis and overall comparison.

**Table 5:** End-to-end cross-species performance comparison.

**Method**	**Pair identification (%)**	**Splice-site position (%)**	**AS-type classification (%)**	**End-to-end accuracy (%)**
***Mouse (Training: Human)***				
**AStrap**	50.8	47.9	65.1	15.7
**DeepASmRNA**	90.7	55.5	72.2	36.2
**MCTASmRNA**	90.7^b^	55.5^b^	52.2	26.3
**MkcDBGAS**	97.1	55.3	73.1	41.2
**IRCAS (Zero-Shot)**	**97.1** ^**a**^	**92.1**	**75.0**	**67.4**
**IRCAS (Fine-tune)**	**97.1** ^**a**^	**92.1**	**81.5**	**73.2**
***Rice (Training: Arabidopsis thaliana)***				
**AStrap**	95.7	52.9	84.1	42.5
**DeepASmRNA**	94.7	49.8	87.5	41.2
**MCTASmRNA**	94.7^b^	49.8^b^	60.8	28.6
**MkcDBGAS**	98.7	50.2	89.9	44.7
**IRCAS (Zero-Shot)**	**98.7**	**93.3**	**88.4**	**81.7**
**IRCAS (Fine-tune)**	**98.7** ^**a**^	**93.3**	**90.3**	**83.4**

^a^Using cDBG pipeline shared with MkcDBGAS.

^b^Using BLAST pipeline shared with DeepASmRNA.

IRCAS demonstrated superior end-to-end performance across both cross-species evaluations (Table 5). On mouse, the zero-shot IRCAS model achieved 67.4% end-to-end accuracy, a 26.2 pp improvement over the previous best method MkcDBGAS (41.2%); the gain is driven primarily by splice-site localization, where IRCAS reaches 92.1% compared with MkcDBGAS’s 55.3%, while transcript-pair identification accuracy is shared across the cDBG-based methods (~97%). On rice, the zero-shot IRCAS model achieved 81.7% end-to-end accuracy versus 44.7% for MkcDBGAS, representing a 37.0 pp improvement under the same protocol. Fine-tuning on a small target-species labeled dataset further improved both transfer settings: on mouse, fine-tuned IRCAS reached 73.2% end-to-end accuracy (a 5.8 pp gain over zero-shot), and on rice 83.4% (a 1.7 pp gain). The mouse improvement was driven mainly by AS-type classification, which rose from 75.0% to 81.5% with fine-tuning; on rice, classification rose from 88.4% to 90.3%. The smaller rice gain reflects that the zero-shot model is already performing well in the plant–plant transfer (*Arabidopsis* → rice), where regulatory and structural features are more similar than in the human → mouse transfer.

Across all baselines, the dominant bottleneck was splice-site localization rather than transcript-pair identification: methods that handled identification well (DeepASmRNA 90.7%, MkcDBGAS 97.1%) still suffered low end-to-end accuracy (36.2%, 41.2%) because of splice-site position errors around 55% (Table 5). This pattern motivates the rectification module that drives IRCAS’s end-to-end gain. Moreover, input format, training/inference compute, supported outputs, and approximate wall-clock time are summarized for qualitative comparison in Supplementary Table S13; these estimates are derived from training logs rather than a controlled benchmark.

### Experimental validation of zero-shot predictions on mouse liver transcriptome

To assess whether IRCAS’s predictions correspond to biologically valid splicing events independent of agreement with reference-based ground-truth labels, we performed Reverse transcription PCR (RT-PCR) validation on mouse liver, applying the zero-shot IRCAS model, the base model trained on human without any mouse-specific fine-tuning. Mouse liver transcripts were assembled from publicly available RNA-seq data [47], and the six predicted AS events with the highest classifier confidence scores were selected for experimental verification. All six predicted events were experimentally confirmed (Supplementary Method S4 available online at http://bib.oxfordjournals.org/, Supplementary Figs S5–S12).

**Figure 8:**
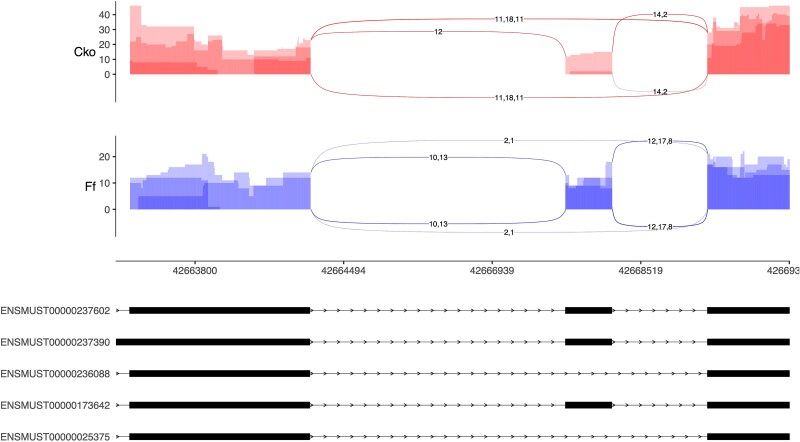
Sashimi plot of *Tcerg1* (chr11:42663800–42669300) in mouse liver showing the cassette exon predicted by zero-shot IRCAS to undergo exon skipping (included in ENSMUST00000237602, skipped in the other four annotated isoforms). RT-PCR yielded amplicons of 362 bp and 299 bp at a ~ 7:3 ratio, in agreement with the prediction.

As a representative example, IRCAS predicted alternative splicing at the *Tcerg1* locus on chromosome 11, involving the isoforms ENSMUST00000025375, ENSMUST00000173642, ENSMUST00000236088, ENSMUST00000237390, and ENSMUST00000237602. RT-PCR using primers flanking the predicted variable region yielded 2 bands of 362 bp and 299 bp, differing by 63 bp in exact agreement with the predicted exon length, with a band-intensity ratio of approximately 7:3 matching the predicted isoform usage in the source RNA-seq data (Fig. 8). This independent experimental confirmation, obtained without reliance on SUPPA2 labels and from a species absent in the training data, supports the validity of IRCAS’s zero-shot predictions.

## Discussion

The development of IRCAS represents a significant advancement in reference-free AS analysis by addressing fundamental limitations that have constrained previous methodologies. Previous approaches treated AS detection and classification as separate processes, creating cascading errors where inaccuracies in splice site prediction directly compromised downstream classification performance and practical application. IRCAS overcomes this limitation through an integrated end-to-end framework comprising three complementary modules: identification, rectification, and classification. The framework is supervised during training on annotated species using SUPPA2-derived labels and reference-free during inference: when applied to a target species, IRCAS requires only an assembled transcriptome and no reference genome, annotation, or reference-based tool. The substantial improvement in splice site localization accuracy (94.3% for human, 96.2% for *Arabidopsis*) compared to existing methods (50%–55%) addresses a critical bottleneck through our attention-based CNN rectification model, which leverages contextual sequence information to capture subtle sequence patterns that define accurate splice site.

The hybrid GNN architecture employed in IRCAS introduces several methodological innovations that collectively enhance AS classification performance. The integration of GAT and Transformer layers addresses complementary aspects of the classification problem: GAT captures local topological relationships within the cDBG structure, while Transformer layers model long-range sequence dependencies that span beyond immediate graph neighborhoods. Our ablation study demonstrates that the classification of AS events strongly relies on both local topological relationships and long-range sequence dependencies. Successful application of IRCAS across diverse species demonstrates the generalizability of learned splice site patterns and AS structural features. The species-specific training strategy, employing separate models for animal and plant, acknowledges fundamental differences in splicing mechanisms while maintaining computational efficiency. Furthermore, our transfer learning approach demonstrates that substantial performance improvements can be achieved with minimal species-specific fine-tuning data, with as few as 10 samples providing meaningful gains for cross-species adaptation. We accordingly report two inference modes throughout the manuscript: zero-shot, using only the pretrained base model on a target species, and fine-tuned, after adaptation on a small target-species labeled set, to clearly separate these two operational settings.

IRCAS demonstrated superior performance across all tested species, achieving 1.5–4.3 percentage point improvements over the previous best method MkcDBGAS. The evaluation revealed distinct performance tiers: traditional feature-based approaches like AStrap showed limited effectiveness (68.6%–84.4%), while deep learning methods including DeepASmRNA and MkcDBGAS achieved competitive results (87.5%–91.1%). Notably, MCTASmRNA exhibited poor performance (32.4%–55.3%), highlighting reproducibility challenges in deep learning AS applications. Particularly noteworthy, IRCAS demonstrated significantly higher accuracy than other models on untrained datasets, exhibiting exceptional robustness. The end-to-end accuracy of 83.4% achieved by fine-tuned IRCAS on Rice dataset demonstrates practical utility for non-model organisms where genome reference is limited.

Despite the significant advances achieved by IRCAS, several limitations warrant acknowledgment.

First, IRCAS operates on assembled mature-mRNA sequences, which constrains classification, particularly for IR events. Branch points, polypyrimidine tracts, and intronic splicing enhancers/silencers that govern intron retention lie outside the mature-mRNA input, and these signals are central to IR regulation. While IRCAS’s IR recall (71% in Fig. 6b) exceeds that of the next-best reference-free method (MkcDBGAS at 60%), the gap to non-IR categories, most pronounced in human, where IR regulation is mechanistically more complex than in *Arabidopsis*, reflects this constraint. Extending classification with pre-mRNA-aware features or pre-mRNA-aware training is a natural direction for future work.

Second, IRCAS predicts splice sites whose flanking sequence patterns fall within the distribution observed during pretraining. For genuinely atypical splicing patterns, including non-canonical splice sites or AS event types outside the SUPPA2 taxonomy, the pretrained model may fail to generalize zero-shot. The fine-tuning protocol allows users to adapt the model using a small labeled dataset on the target organism. AS mechanisms fundamentally outside the seven SUPPA2 categories are not directly discoverable without category-extended retraining; this is a constraint of the supervised training paradigm and a direction for future work.

Third, the classification performance for animal species remains suboptimal due to the inherent complexity of animal AS events, which exhibit greater structural diversity and regulatory heterogeneity compared to plant systems. Future work could explore alternative deep learning architectures or implement species-specific subclassification within animal lineages to better capture this complexity.

Fourth, the computational overhead associated with constructing individual cDBG representations for each transcript pair significantly impacts training efficiency, particularly when scaling to large transcriptomic datasets. Alternative graph construction strategies or more efficient graph representation methods could address this bottleneck while maintaining the biological interpretability that makes the cDBG approach effective for AS detection.

**Key Points**

- End-to-end reference-free AS detection framework.
- 92%–96% splice site accuracy via CNN rectification.
- GNN-Transformer hybrid for robust AS classification.

## Supplementary Material

supplementary_data_final_bbag384

## Data Availability

The code of IRCAS is available at https://github.com/S444shen/IRCAS. All relevant data is available at http://zhangqblab.cn/IRCAS/index.php/download.
