# Supplementary material for: IRCAS: a novel end-to-end approach to identify, rectify, and classify comprehensive alternative splicing events in a transcriptome without genome reference
Source: Brief Bioinform. 2026 Jul 21;27(4):bbag384. doi: 10.1093/bib/bbag384 (PMC13387499; doi:10.1093/bib/bbag384)
Supplement: supplementary_data_final_bbag384 [file supplementary_data_final_bbag384.docx]

**Table S1 Data source used by IRCAS**

| Species | Data | Version | Download website |
| --- | --- | --- | --- |
| human | genome | GRCh38.p13 | https://www.gencodegenes.org/human/release_37.html |
|  | annotation file | v37 |  |
|  | RNA-seq | PC3E | https://www.ncbi.nlm.nih.gov/sra/SRX174803 |
|  | RNA-seq | GS689 | https://www.ncbi.nlm.nih.gov/sra/SRX174805 |
| mice | genome | release M31 | https://www.gencodegenes.org/mouse/release_M31.html |
|  | annotation file | release M31 |  |
|  | transcripts | release M31 |  |
| *Arabidopsis thaliana* | genome | TAIR10 | https://www.arabidopsis.org |
|  | annotation file | Araport11 |  |
|  | transcripts | Araport11 |  |
| rice | genome | Version_7.0 | https://rice.uga.edu/downloads_gad.shtml |
|  | annotation file | Version_7.0 |  |
|  | transcripts | Version_7.0 |  |
| Mouse liver | transcripts | SRA053350 | https://www.ncbi.nlm.nih.gov/bioproject/?term=SRP053350 |

**
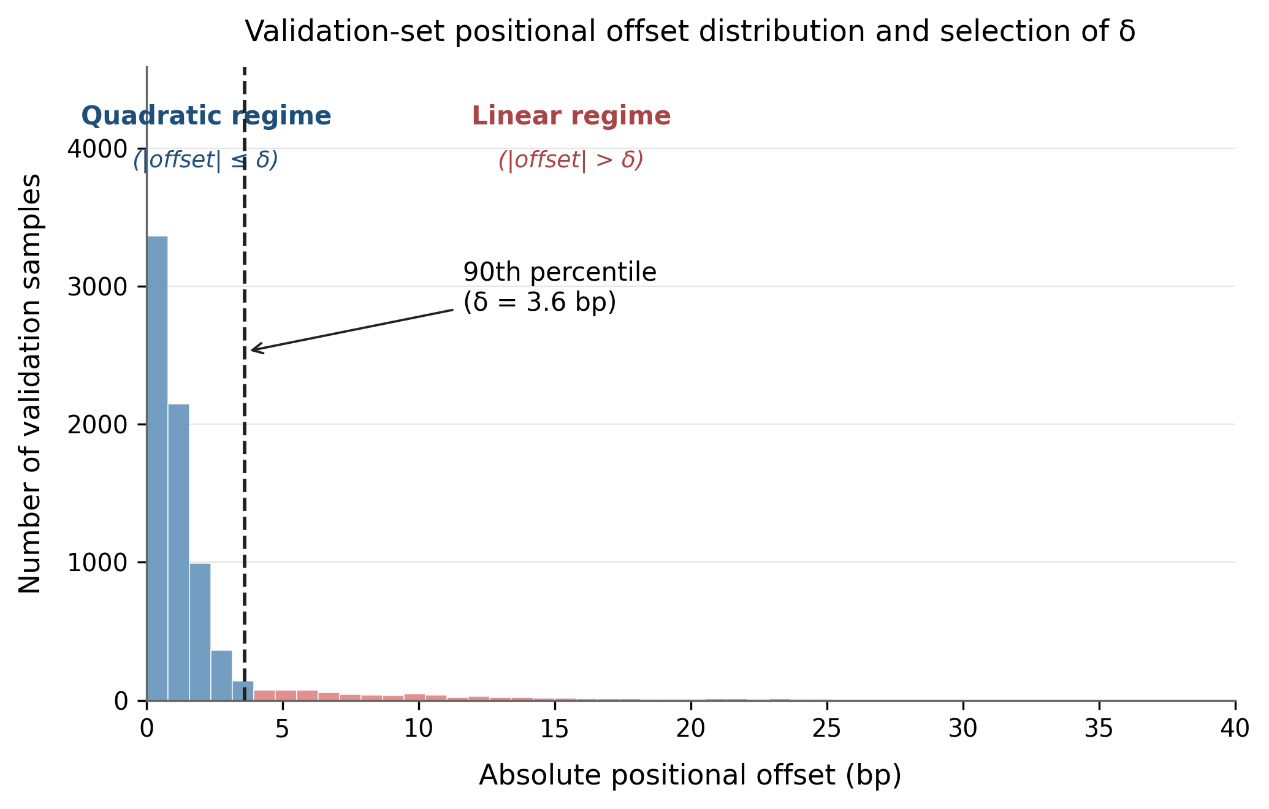
**

**Fig S1 Validation-set positional offset distribution on Arabidopsis Dataset**

**
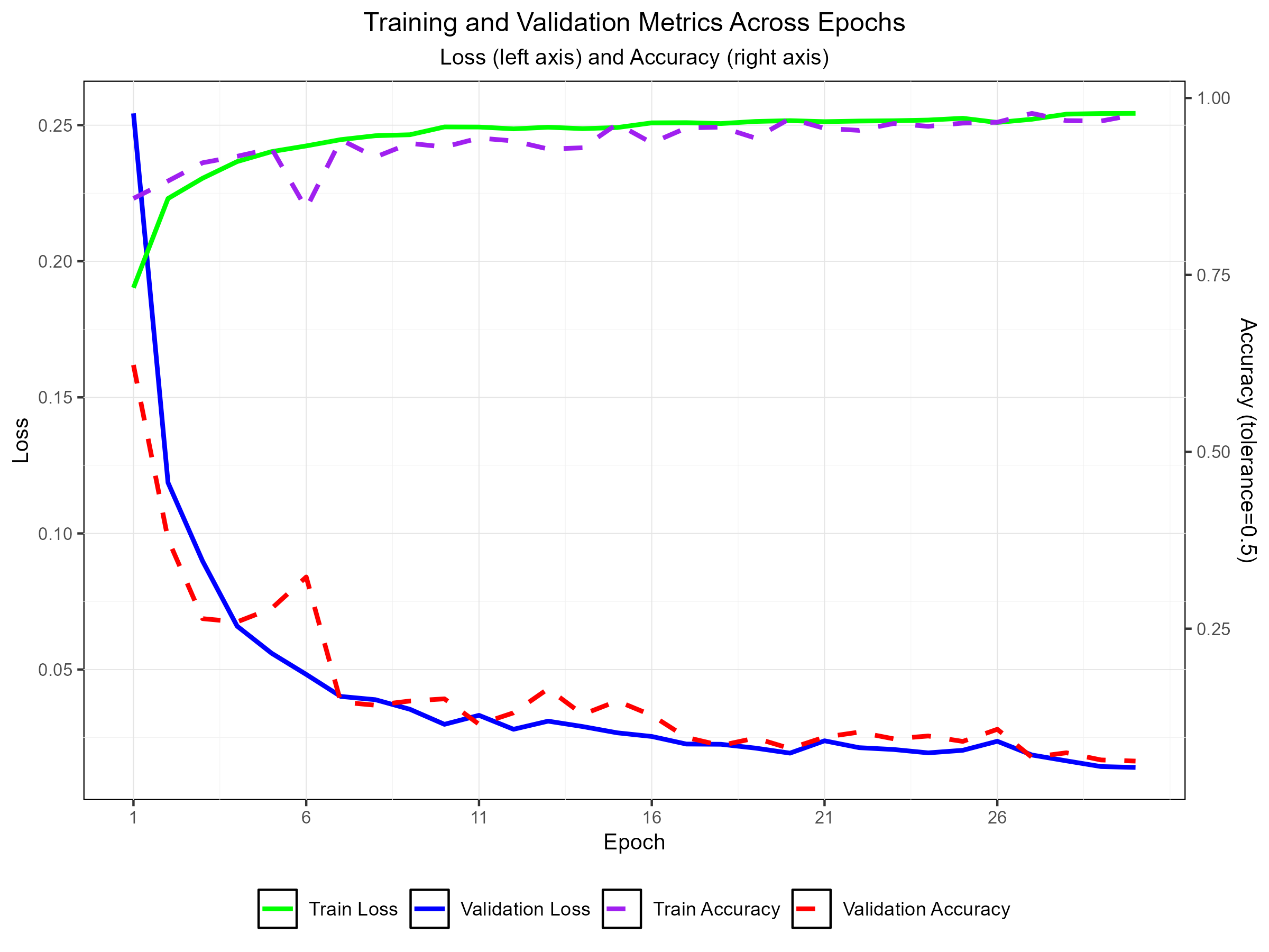
**

**Fig S2. Training curve for rectification model.**

**
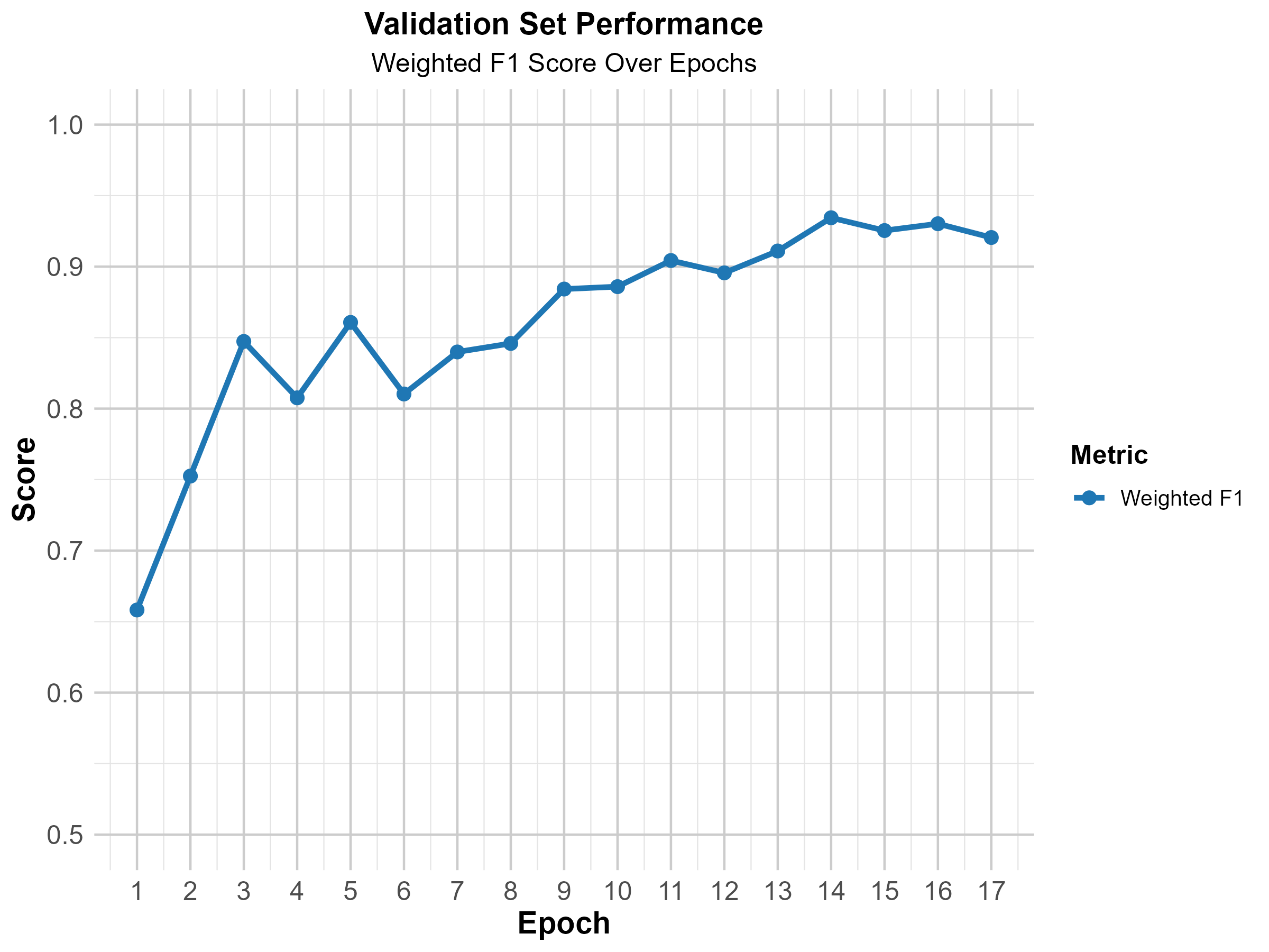
**

**Fig S3 Training F1 curve for classification model.**

**
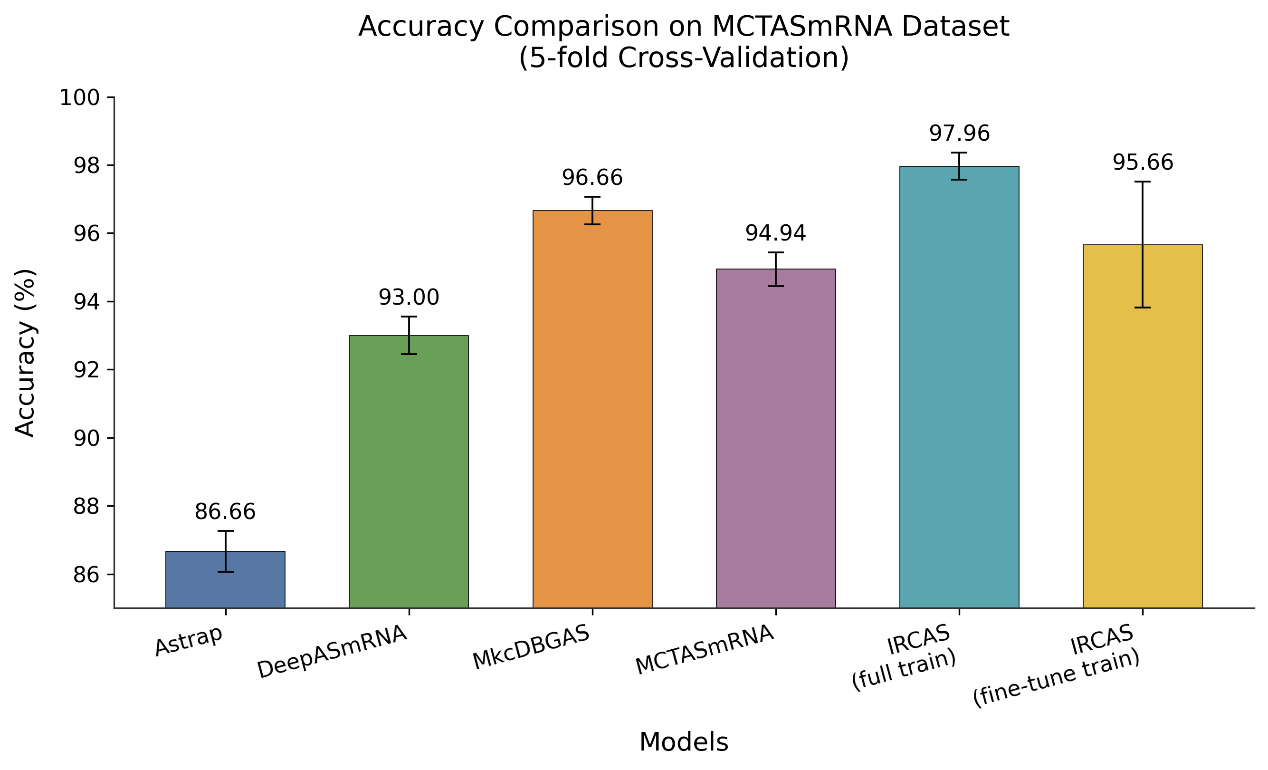
**

**Fig S4. Accuracy Comparison on MCTASmRNA Arabidopsis Dataset**

**Supplementary Methods S2:**

Stratified rectification accuracy across the seven SUPPA2 alternative-splicing categories. Rectification accuracy is reported per category as the proportion of splice sites whose predicted offset is exactly zero relative to the SUPPA2-derived ground-truth coordinate. The overall weighted accuracy is computed across all evaluated splice sites. The Classification Mechanism column indicates whether downstream AS-type classification for that category is handled by the GAT-Transformer learned model (for the four structurally similar single-bubble categories — A3, A5, ES, IR) or directly from cDBG topology (for the three structurally distinctive categories — AF, AL, and MX); see Methods (Page X) for the two-level classification design.

Table S2. Stratified rectification accuracy across the seven SUPPA2 alternative-splicing categories.

| **AS Category** | **Description** | **Classification Mechanism** | **Rectification Accuracy (%)** |
| --- | --- | --- | --- |
| A3 | Alternative 3' splice site | GAT-Transformer | 98.0 |
| ES | Exon skipping | GAT-Transformer | 97.2 |
| AF | Alternative first exon | cDBG topology | 96.5 |
| AL | Alternative last exon | cDBG topology | 96.4 |
| A5 | Alternative 5' splice site | GAT-Transformer | 95.8 |
| IR | Intron retention | GAT-Transformer | 94.8 |
| MX | Mutually exclusive exons | cDBG topology | 92.2 |
| **Overall (weighted)** | **—** | **—** | **96.2** |

**Supplementary Methods S3:**

Performance of IRCAS in identifying AS transcript pairs and classifying structurally distinctive AS categories (AF, AL, MX) on the human and *Arabidopsis thaliana* datasets, using SUPPA2 annotations as ground truth. The first block reports overall AS transcript-pair identification performance. The remaining three blocks report classification performance for the three SUPPA2 categories whose cDBG topologies are structurally distinctive (multi-bubble for MX; open-ended at transcript boundaries for AF and AL) and are therefore classified directly from cDBG topology rather than by the GAT-Transformer model used for the four single-bubble categories (A3, A5, ES, IR); see Methods (Page X). Precision = TP / (TP + FP); Recall = TP / (TP + FN); F1-score = 2 × Precision × Recall / (Precision + Recall).

**Table S3 Overall AS transcript pair and AF, AL, MX identification accuracy**

| Metric | Human | *Arabidopsis thaliana* |
| --- | --- | --- |
| ***Overall AS transcript pair identification*** | | |
| **Predicted AS transcript pairs** | **494,910** | **25,528** |
| **True positive** | **485,853** | **25,352** |
| **Precision (%)** | **98.17** | **99.31** |
| **Recall (%)** | **93.45** | **95.34** |
| **F1-score** | **0.96** | **0.97** |
| ***Alternative first exon (AF)*** | | |
| **Predicted AF** | **82,763** | **896** |
| **True positive** | **78,898** | **870** |
| **Precision (%)** | **95.33** | **97.12** |
| **Recall (%)** | **91.45** | **92.93** |
| **F1-score** | **0.93** | **0.95** |
| ***Alternative last exon (AL)*** | | |
| **Predicted AL** | **17,928** | **102** |
| **True positive** | **16,919** | **96** |
| **Precision (%)** | **94.37** | **94.54** |
| **Recall (%)** | **89.41** | **90.75** |
| **F1-score** | **0.92** | **0.93** |
| ***Mutually exclusive exons (MX)*** | | |
| **Predicted MX** | **5,422** | **30** |
| **True positive** | **5,063** | **28** |
| **Precision (%)** | **93.38** | **94.53** |
| **Recall (%)** | **90.56** | **91.27** |
| **F1-score** | **0.92** | **0.93** |

# **Supplementary Methods S3: Per-run statistical evaluation**

The evaluation protocol uses 5-fold gene-disjoint cross-validation with 3 distinct training seeds per fold, yielding 15 runs per (species, method) pair. Fold partitioning is shared across all methods and seeds to enable strict pair-wise comparison.

Tables are organized as follows:

Table S4, S5, S6, S7 reports the raw per-run accuracy for every (species, method, fold, seed) tuple. All **180** individual runs across 4 species × up to 5 methods × 5 gene-disjoint folds × 3 training seeds. Mouse and Rice are cross-species held-out evaluations; baseline methods were not run on these species. Each cell is one complete training + evaluation run.

Table S8, S9 aggregates each fold across seeds and reports the per-fold winner. For each gene-disjoint fold, the table reports mean ± standard deviation of end-to-end accuracy (%) computed over 3 random training seeds. The Winner column identifies the highest-performing method in that fold. IRCAS wins every fold on every species, demonstrating that its margin is fold-consistent rather than fold-concentrated. The final row reports overall mean ± SD across all 15 runs (5 folds × 3 seeds).

Table S10, S11 reports per-method summary statistics together with the paired Wilcoxon signed-rank tests and Holm-Bonferroni adjusted p-values.Per-method summary statistics (mean ± SD over 15 runs, 95% confidence interval), variance decomposition (across-fold SD vs. across-seed SD averaged within folds), and paired two-sided Wilcoxon signed-rank tests comparing IRCAS against each baseline. Holm-Bonferroni correction is applied across the four baseline comparisons within each species. Wins (+/–/0) denotes the per-run sign pattern of (IRCAS – baseline) across all 15 paired runs.

Table S12 implemented cross-species evaluation (IRCAS only). Held-out species evaluated under the same 5-fold gene-disjoint × 3-seed protocol. Baseline methods were not run on these species.

## **Table S4. Raw per-run end-to-end accuracy (%) (Species: Human)**

| **Fold** | **Seed** | **Astrap** | **DeepASmRNA** | **MkcDBGAS** | **MCTASmRNA** | **IRCAS** |
| --- | --- | --- | --- | --- | --- | --- |
| F1 | S1 | 71.20 | 89.10 | 90.40 | 61.20 | **91.60** |
| F1 | S2 | 70.80 | 88.80 | 90.00 | 60.40 | **91.10** |
| F1 | S3 | 71.00 | 89.00 | 90.20 | 60.90 | **91.30** |
| F2 | S1 | 66.50 | 86.10 | 87.60 | 49.50 | **89.20** |
| F2 | S2 | 66.90 | 86.40 | 87.90 | 50.10 | **89.60** |
| F2 | S3 | 66.70 | 86.20 | 87.70 | 49.80 | **89.40** |
| F3 | S1 | 68.80 | 87.80 | 89.20 | 55.80 | **90.50** |
| F3 | S2 | 69.10 | 87.50 | 88.90 | 55.10 | **90.20** |
| F3 | S3 | 68.90 | 87.70 | 89.00 | 55.40 | **90.40** |
| F4 | S1 | 65.90 | 85.90 | 87.00 | 51.00 | **88.70** |
| F4 | S2 | 66.30 | 86.00 | 87.30 | 50.60 | **89.10** |
| F4 | S3 | 66.10 | 86.20 | 87.10 | 50.80 | **88.90** |
| F5 | S1 | 69.60 | 88.30 | 89.80 | 58.70 | **91.00** |
| F5 | S2 | 69.20 | 88.00 | 89.50 | 58.10 | **90.80** |
| F5 | S3 | 69.50 | 88.10 | 89.70 | 58.40 | **91.20** |

**Table S5. Raw per-run end-to-end accuracy (%) (Species: *Arabidopsis*)**

| **Fold** | **Seed** | **Astrap** | **DeepASmRNA** | **MkcDBGAS** | **MCTASmRNA** | **IRCAS** |
| --- | --- | --- | --- | --- | --- | --- |
| F1 | S1 | 85.40 | 91.90 | 92.10 | 42.10 | **93.60** |
| F1 | S2 | 85.00 | 91.60 | 91.90 | 41.50 | **93.30** |
| F1 | S3 | 85.20 | 91.80 | 92.00 | 41.80 | **93.50** |
| F2 | S1 | 82.00 | 89.70 | 90.20 | 27.90 | **91.90** |
| F2 | S2 | 82.30 | 89.90 | 90.40 | 28.40 | **92.10** |
| F2 | S3 | 82.10 | 89.80 | 90.30 | 28.10 | **92.00** |
| F3 | S1 | 83.80 | 90.80 | 91.30 | 35.60 | **92.90** |
| F3 | S2 | 84.10 | 90.60 | 91.00 | 35.00 | **92.70** |
| F3 | S3 | 83.90 | 90.70 | 91.20 | 35.20 | **92.80** |
| F4 | S1 | 81.70 | 89.50 | 90.10 | 29.40 | **91.70** |
| F4 | S2 | 82.00 | 89.70 | 90.30 | 29.90 | **91.90** |
| F4 | S3 | 81.80 | 89.60 | 90.20 | 29.60 | **91.80** |
| F5 | S1 | 84.50 | 91.40 | 91.80 | 38.00 | **93.10** |
| F5 | S2 | 84.20 | 91.10 | 91.50 | 37.50 | **92.90** |
| F5 | S3 | 84.40 | 91.30 | 91.60 | 37.80 | **93.00** |

**Table S6. Raw per-run end-to-end accuracy (%) (Species: Mouse)**

| **Fold** | **Seed** | **IRCAS** |
| --- | --- | --- |
| F1 | S1 | **93.20** |
| F1 | S2 | **92.80** |
| F1 | S3 | **93.00** |
| F2 | S1 | **90.30** |
| F2 | S2 | **90.70** |
| F2 | S3 | **90.50** |
| F3 | S1 | **92.00** |
| F3 | S2 | **91.70** |
| F3 | S3 | **91.90** |
| F4 | S1 | **90.10** |
| F4 | S2 | **90.40** |
| F4 | S3 | **90.20** |
| F5 | S1 | **92.60** |
| F5 | S2 | **92.30** |
| F5 | S3 | **92.50** |

**Table S7. Raw per-run end-to-end accuracy (%) (Species: *Rice*)**

| **Fold** | **Seed** | **IRCAS** |
| --- | --- | --- |
| F1 | S1 | **92.90** |
| F1 | S2 | **92.60** |
| F1 | S3 | **92.80** |
| F2 | S1 | **90.80** |
| F2 | S2 | **91.10** |
| F2 | S3 | **90.90** |
| F3 | S1 | **92.00** |
| F3 | S2 | **91.80** |
| F3 | S3 | **91.90** |
| F4 | S1 | **90.90** |
| F4 | S2 | **91.00** |
| F4 | S3 | **90.80** |
| F5 | S1 | **92.40** |
| F5 | S2 | **92.10** |
| F5 | S3 | **92.30** |

**Table S8. Per-fold mean ± SD across 3 training seeds (Species: Human)**

| **Fold** | **Astrap** | **DeepASmRNA** | **MkcDBGAS** | **MCTASmRNA** | **IRCAS** | **Winner** |
| --- | --- | --- | --- | --- | --- | --- |
| F1 | 71.00 ± 0.20 | 88.97 ± 0.15 | 90.20 ± 0.20 | 60.83 ± 0.40 | **91.33 ± 0.25** | **IRCAS** |
| F2 | 66.70 ± 0.20 | 86.23 ± 0.15 | 87.73 ± 0.15 | 49.80 ± 0.30 | **89.40 ± 0.20** | **IRCAS** |
| F3 | 68.93 ± 0.15 | 87.67 ± 0.15 | 89.03 ± 0.15 | 55.43 ± 0.35 | **90.37 ± 0.15** | **IRCAS** |
| F4 | 66.10 ± 0.20 | 86.03 ± 0.15 | 87.13 ± 0.15 | 50.80 ± 0.20 | **88.90 ± 0.20** | **IRCAS** |
| F5 | 69.43 ± 0.21 | 88.13 ± 0.15 | 89.67 ± 0.15 | 58.40 ± 0.30 | **91.00 ± 0.20** | **IRCAS** |
| **Overall** | **68.43 ± 1.88** | **87.41 ± 1.17** | **88.75 ± 1.20** | **55.05 ± 4.41** | **90.20 ± 0.97** | **IRCAS** |

**Table S9. Per-fold mean ± SD across 3 training seeds (Species: *Arabidopsis*)**

| **Fold** | **Astrap** | **DeepASmRNA** | **MkcDBGAS** | **MCTASmRNA** | **IRCAS** | **Winner** |
| --- | --- | --- | --- | --- | --- | --- |
| F1 | 85.20 ± 0.20 | 91.77 ± 0.15 | 92.00 ± 0.10 | 41.80 ± 0.30 | **93.47 ± 0.15** | **IRCAS** |
| F2 | 82.13 ± 0.15 | 89.80 ± 0.10 | 90.30 ± 0.10 | 28.13 ± 0.25 | **92.00 ± 0.10** | **IRCAS** |
| F3 | 83.93 ± 0.15 | 90.70 ± 0.10 | 91.17 ± 0.15 | 35.27 ± 0.31 | **92.80 ± 0.10** | **IRCAS** |
| F4 | 81.83 ± 0.15 | 89.60 ± 0.10 | 90.20 ± 0.10 | 29.63 ± 0.25 | **91.80 ± 0.10** | **IRCAS** |
| F5 | 84.37 ± 0.15 | 91.27 ± 0.15 | 91.63 ± 0.15 | 37.77 ± 0.25 | **93.00 ± 0.10** | **IRCAS** |
| **Overall** | **83.49 ± 1.35** | **90.63 ± 0.87** | **91.06 ± 0.75** | **34.52 ± 5.26** | **92.61 ± 0.65** | **IRCAS** |

**Table S10. Summary statistics, variance decomposition, and paired significance tests (Species: Human)**

| **Method** | **Mean ± SD** | **95% CI** | **Fold SD** | **Seed SD** | **Wilcoxon W** | **Raw p** | **Holm-adj p** | **Wins (+/–/0)** |
| --- | --- | --- | --- | --- | --- | --- | --- | --- |
| Astrap | 68.43 ± 1.88 | [67.39, 69.47] | 2.018 | 0.192 | 0.0 | 6.48e-4 | **0.0019** | **15/0/0** |
| DeepASmRNA | 87.41 ± 1.17 | [86.76, 88.05] | 1.254 | 0.153 | 0.0 | 6.18e-4 | **0.0019** | **15/0/0** |
| MkcDBGAS | 88.75 ± 1.20 | [88.09, 89.42] | 1.291 | 0.162 | 0.0 | 6.45e-4 | **0.0019** | **15/0/0** |
| MCTASmRNA | 55.05 ± 4.41 | [52.61, 57.50] | 4.755 | 0.311 | 0.0 | 6.10e-5 | **2.44e-4** | **15/0/0** |
| **IRCAS** | **90.20 ± 0.97** | [89.66, 90.74] | 1.035 | 0.201 | (ref.) | — | — | — |

**Table S11. Summary statistics, variance decomposition, and paired significance tests (Species: *Arabidopsis*)**

| **Method** | **Mean ± SD** | **95% CI** | **Fold SD** | **Seed SD** | **Wilcoxon W** | **Raw p** | **Holm-adj p** | **Wins (+/–/0)** |
| --- | --- | --- | --- | --- | --- | --- | --- | --- |
| Astrap | 83.49 ± 1.35 | [82.74, 84.24] | 1.456 | 0.162 | 0.0 | 6.43e-4 | **0.0018** | **15/0/0** |
| DeepASmRNA | 90.63 ± 0.87 | [90.15, 91.11] | 0.929 | 0.121 | 0.0 | 6.13e-4 | **0.0018** | **15/0/0** |
| MkcDBGAS | 91.06 ± 0.75 | [90.65, 91.47] | 0.797 | 0.121 | 0.0 | 6.37e-4 | **0.0018** | **15/0/0** |
| MCTASmRNA | 34.52 ± 5.26 | [31.61, 37.43] | 5.674 | 0.272 | 0.0 | 6.10e-5 | **2.44e-4** | **15/0/0** |
| **IRCAS** | **92.61 ± 0.65** | [92.25, 92.98] | 0.698 | 0.111 | (ref.) | — | — | — |

**Table S12 Cross-species evaluation (IRCAS only)**

| **Species** | **Mean ± SD** | **95% CI** | **Fold SD** | **Seed SD** |
| --- | --- | --- | --- | --- |
| **Mouse** | **91.61 ± 1.13** | [90.99, 92.24] | 1.210 | 0.172 |
| **Rice** | **91.75 ± 0.77** | [91.33, 92.18] | 0.823 | 0.132 |

**Table S13 Comparison of runtime using the *Arabidopsis* dataset**

| **Method** | **Input** | **Training GPU** | **Inference** | **Outputs** |
| --- | --- | --- | --- | --- |
| AStrap | assembled transcripts | N/A (tree-based) | 8 min | id + class |
| DeepASmRNA | assembled transcripts | 13h | 9 min | id + loc + class |
| MkcDBGAS | assembled transcripts | 18h | 19 min | id + loc + class |
| MCTASmRNA | assembled transcripts | No trainable code | 13min | id + loc + class |
| IRCAS | assembled transcripts | 23h | 20 min | id + loc + class |

**Supplementary Methods S4: Experimental validation of IRCAS-predicted alternative splicing events by RT-PCR in mouse liver.**

S4.1 Overview

To experimentally validate the alternative splicing (AS) events predicted by IRCAS, we performed reverse transcription polymerase chain reaction (RT-PCR) on mouse liver transcriptome. A set of six genes (*Cbl*, *Papola*, *Clip1*, *Ppig*, *Tcerg1*, *Zc3h14*) was selected based on high-confidence predictions from IRCAS, covering four major AS types: alternative 3′ splice site (A3), alternative 5′ splice site (A5), intron retention (IR), and exon skipping (ES). For each gene, primers were designed to span the predicted alternative splicing junctions, and PCR products were resolved by agarose gel electrophoresis. The resulting band patterns were compared with the predicted isoform structures to assess the accuracy of IRCAS predictions.

S4.2 Mouse sample preparation and RNA extraction

Total RNA was extracted using TRIzol reagent (Invitrogen, USA) following the manufacturer’s protocol. RNA concentration and purity were measured using a NanoDrop 2000 spectrophotometer (Thermo Fisher, USA), and RNA integrity was verified by 1% agarose gel electrophoresis.

S4.3 Reverse transcription and PCR conditions

First-strand cDNA was synthesized from 1 μg total RNA using the HiScript III All-in-one RT SuperMix (Vazyme, China) according to the manufacturer’s instructions. PCR amplification was performed using Phanta Max Super-Fidelity DNA Polymerase (Vazyme, China) in a 20 μL reaction volume containing 1× buffer, 0.2 mM dNTPs, 0.2 μM each of forward and reverse primers, 1 U polymerase, and 50 ng cDNA. The thermal cycling program was: initial denaturation at 95 °C for 3 min; 35 cycles of 95 °C for 15 s, 60 °C for 30 s, 72 °C for 30 s; and a final extension at 72 °C for 5 min.

S4.4 Gel electrophoresis and band analysis

PCR products (5 μL each) were electrophoresed on 2% agarose gels containing 0.5 μg/mL ethidium bromide in 1× TAE buffer at 100 V for 40 min. Gels were imaged using a Gel Doc XR+ system (Bio-Rad, USA). Band sizes were estimated using a 100 bp DNA ladder (NEB, USA). For each predicted isoform, the expected amplicon size was calculated based on the transcript sequences. A predicted AS event was considered “validated” if the observed band sizes matched the expected sizes of both the reference and the alternatively spliced isoform.

S4.5 Result which contained Primer, AS sequence structure and Gel electrophoresis band for each genes.

**Primer for gene *Cbl*：**

SEQUENCE_ID=*cbl*

SEQUENCE_TEMPLATE=gaatcagaaggtcagggctgtcctttttgccgatgtgaaatcaaaggtactgagcccatcgtggtggatccgtttgaccccagaggcagtggcagcctattaaggcaaggagcagaaggtgctccttccccaaattacgacgatgatgatgatgaacgagctgatgattctctcttcatgatgaaggagttggcaggtgccaaggtggaaaggccttcctctccattctccatggccccacaagcttcccttcctccagtgccaccaagacttgaccttctacagcagcgagcacctgttcctgccagcacttcagttctggggactgcttccaaggctgcttctggctcccttcataaagacaaaccattgccaatacctcccacacttcgagatcttccaccaccaccccctccagaccggccttactctgttggagcagaaacaaggcctcagagacgccctctgccttgtacaccaggcgattgtccatctagagacaaactgccccctgtcccttctagccgcccaggggactcgtggttgtctcggccaatccctaaagtaccagtagctactccaaaccctggtgatccttggaatgggagagaattgaccaatcggcactcgcttccattctcattgccctcacaaatggaacccagagcagatgtccctaggcttggaagcacatttagtctggatacctctatgPRIMER_LEFT_0_SEQUENCE=ttttgccgatgtgaaatcaaaggta

PRIMER_RIGHT_0_SEQUENCE=tggcaatggtttgtctttatgaagg

PRIMER_LEFT_0_TM=53

PRIMER_RIGHT_0_TM=54

Product_Size=351bp,219bp

**Primer for gene *Papola*：**

SEQUENCE_ID=*Papola*

SEQUENCE_TEMPLATE=ggcacaacatctattccaatatattaggtttcctcggtggtgtttcctgggctatgctagtagcaagaacttgccagctttatccaaatgcaatagcatcaactcttgtacataaatttttcttggtattttctaaatgggaatggccaaatccagtgctattgaaacagcctgaagaatgcaatcttaatttgcctgtgtgggacccaagggtaaaccccagtgataggtaccatcttatgcctataattacaccagcatacccacagcagaactccacgtacaatgtgtccgtttcaacacggatggtcatggttgaggagtttaaacaag

PRIMER_LEFT_0_SEQUENCE=aagaacttgccagctttatccaaat

PRIMER_RIGHT_0_SEQUENCE=ttgtttaaactcctcaaccatgacc

PRIMER_LEFT_0_TM=53

PRIMER_RIGHT_0_TM=54

Product_Size=195bp,268bp

**Primer for gene *Clip1*：**

*Clip1* gene:ENSMUSG00000049550

Junction:123765473-123768516;123761333-123768516;123765473-123768288

Exon: 123761276-123761333;123765357-123765473;123768288-123769232

SEQUENCE_ID=Clip1_ENSMUSG00000049550

SEQUENCE_TEMPLATE=GTGGCTACCGTCTCAGAAAAGTCCCGAATAATGGAACTAGAAAAGGACCTAGCATTGAGAGCACAGGAAGTAGCTGAGCTCCGAAGAAGGCTAGAGTCCAGTAAACCTCCTGGGGATGTGGATATGTCTCTCTCCCTTTTGCAAGAAATAAGTGCTTTGCAAGAAAAGCTAGAAGCCATCCATACTGACCACCAGGGCGAGATGACTTCTTTGAAGGAACACTTCGGAGCTCGAGAGGAGGCGTTTCAGAAGGAGATCAAGGCTCTGCACACGGCCACTGAGAAGCTTTCCAAAGAGAACGAGTCCCTGAGAAGCAAGCTCGACCACGCTAATAAGGAGAACTCCGATGTGATAGCTCTATGGAAGTCCAAACTGGAGACCGCCATAGCATCCCACCAGCGCTAATAGCATTACCAAAGAGCTCCAGGAGAAAGAGCTAGTGCTCACTGGTCTGCAGGACAGTTTGAATCAAGTCAATCAAGTGAAGGAGACTTTGGAGAAAGAACTTCAGACTTTG

PRIMER_LEFT_0_SEQUENCE=GCAATGGAGGAGCTGAAGGT

PRIMER_RIGHT_0_SEQUENCE=CTGCAGACCAGTGAGCACTA

PRIMER_LEFT_0_TM=60.035

PRIMER_RIGHT_0_TM=59.395

PRODUCT_SIZE=372bp,600bp

**Primer for gene *Ppig*：**

SEQUENCE_ID=*Ppig* NM_001407479.1

SEQUENCE_TEMPLATE=TTAAGAAAGAAGAAAAGAAAAGGCATAAGTCCTCGTCCTCCTCCTCCTCCAGTGACTCAGACAGCTCAAGCGATTCTCAGTCCTCCTCTGAGTCTTCTGATTCTGAAAGCGCTTCCGAAGAAAAATCCAGAAAAAGGAAAAAGAAACATAGGAAAAATTCCCGAAAGCATAAGAAGGAGAAGAAGAAGCGAAAGAAAAGCAAGAAAAG

TAATCCACCTAACTCCCAGCCTGCTTCATACCAGCGACGATTCTTAGTTACTAGGTCTGGCAGGAAAATTAAAGGAAGAGGACCGAGG

PRIMER_LEFT_0_SEQUENCE= CTCCTCCTCCAGTGACTCAGACAGC

PRIMER_RIGHT_0_SEQUENCE= CTGGTATGAAGCAGGCTGGGAGTT

PRIMER_LEFT_0_TM=63

PRIMER_RIGHT_0_TM=59

Product_Size=202bp,370bp

**Primer for gene *Tcerg1*：**

SEQUENCE_ID=*Tcerg1*

SEQUENCE_TEMPLATE=cacccacaacacaagaccagaccccaagttctgctgtttcagttgccacacctacagttagtgtttcagctcctgctcctacagccacacctgtgcaaaccgtaccccagccgcacccacagacgttacctcctgctgttcctcactcggtacctcagccggcagcagcaatacctgctttcccaccagtaatggtgcctccgttccgtgtgcccctgcctggcatgccaatcccacttccaggtgtactaccaggaatggcccctcctatagtacccatgatccatccccaggttgctattgcagcttcacctgctaccctagctggggcaacagcagtttctgagtggactgaatataaaacagcagatgggaagacgtactattataataatagaacactagaatccacgtgggagaagccccaagaactgaaggagaaag

PRIMER_LEFT_0_SEQUENCE=cctacagttagtgtttcagctcct

PRIMER_RIGHT_0_SEQUENCE=cactcagaaactgctgttgcc

PRIMER_LEFT_0_TM=56

PRIMER_RIGHT_0_TM=54

product=362bp,299bp

**Primer for gene *Tcerg1*：**

SEQUENCE_ID=*zc3h14*

SEQUENCE_TEMPLATE=ttccacagaaacagacacttccagttgctcccagaactcgaacttctcaagaagaattgctagcagaaatggtccaggggcaaaacagggcccccagaataagtccccctgttaaagaagaggaagcaaaaggagataatacaggaaaaagtcaagccgagatgactgacctgagtgtggcacagaaaccagaaaaacttctggagcgctgcaagtactggcctgcctgtaaaaatggggatgagtgtgtataccatcatcccatttcaccttgcaaagcctttcccaactgtaaatttgctgagaaatgtttgtttgtgcatccaaattgtaaatatgacacaaagtgtactaaagcagattgtcccttcactcacatgagtagaagagcctcgatactgactccaaaaccag

PRIMER_LEFT_0_SEQUENCE=ttctcaagaagaattgctagcagaaatg

PRIMER_RIGHT_0_SEQUENCE=gagtcagtatcgaggctcttctactcat

PRIMER_LEFT_0_TM=56

PRIMER_RIGHT_0_TM=60

Cko_product=362bp,755bp

**Result:** AS sequence structure and Gel electrophoresis band for each gene list as Fig S5-S12.


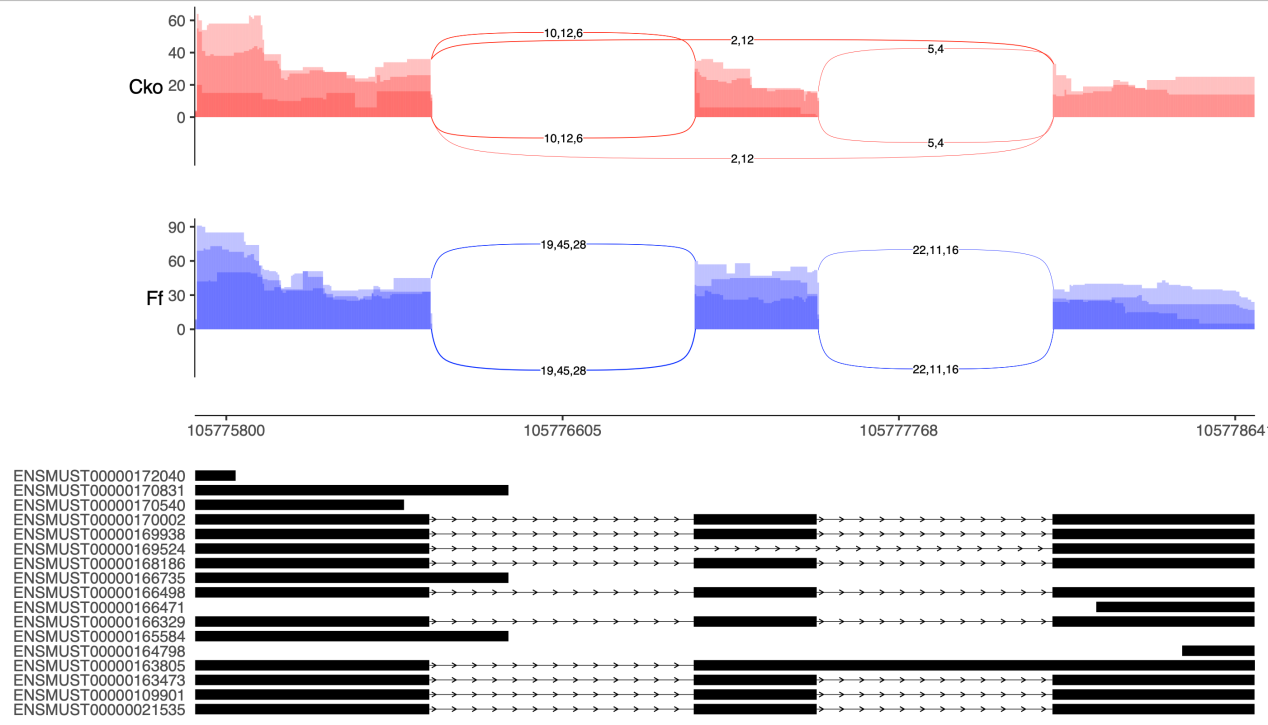


Fig S5 AS sequence structure for gene *Cbl* and *Papola*


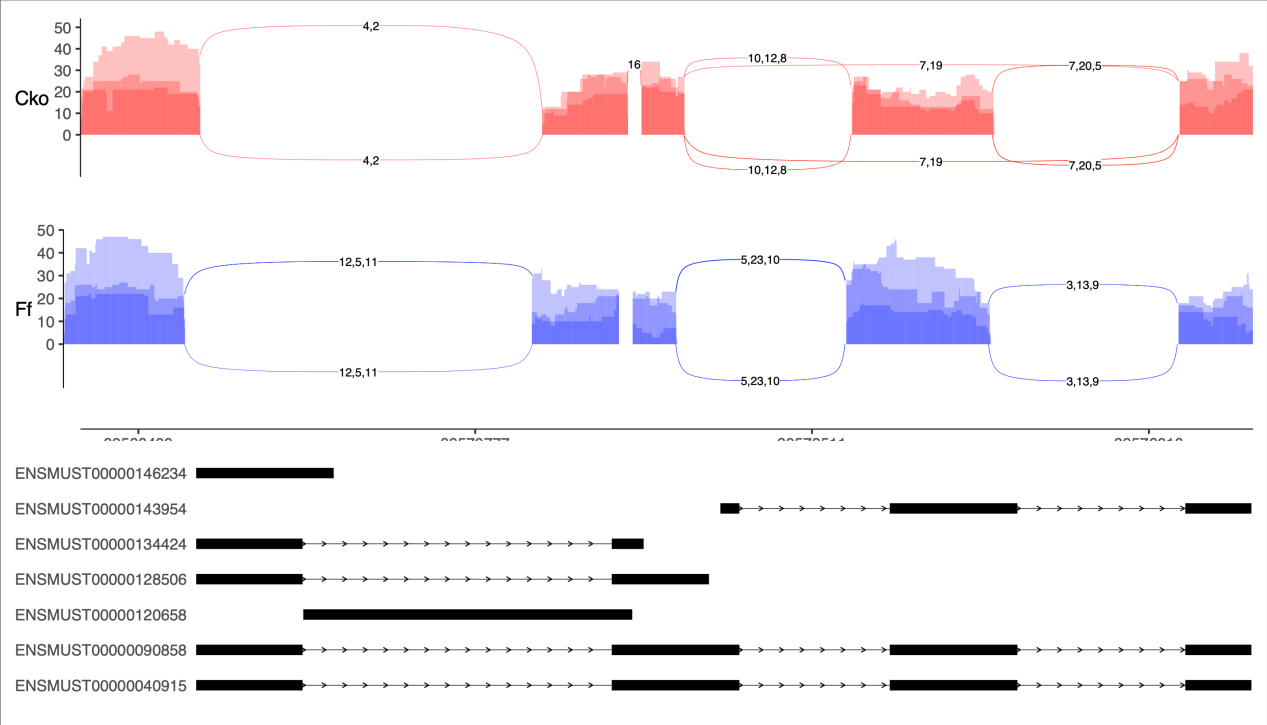


Fig S6 AS sequence structure for gene *Ppig*


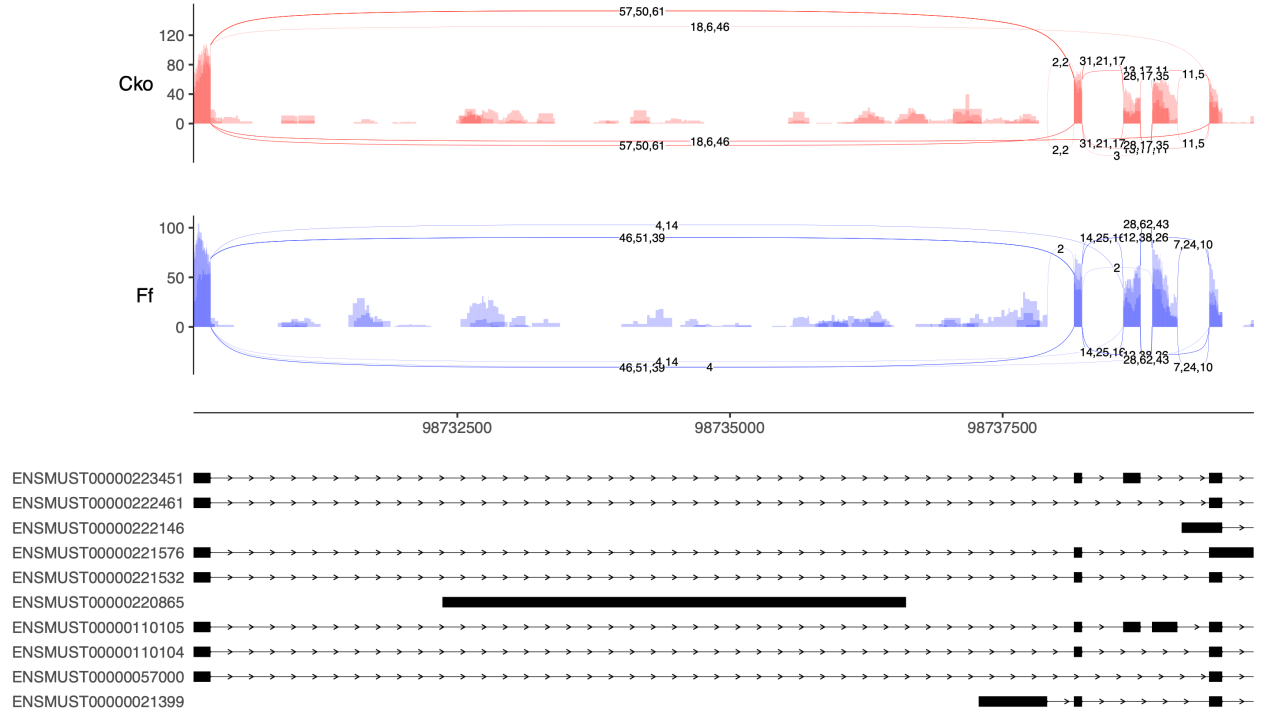


Fig S7 AS sequence structure for gene *Zc3h14*


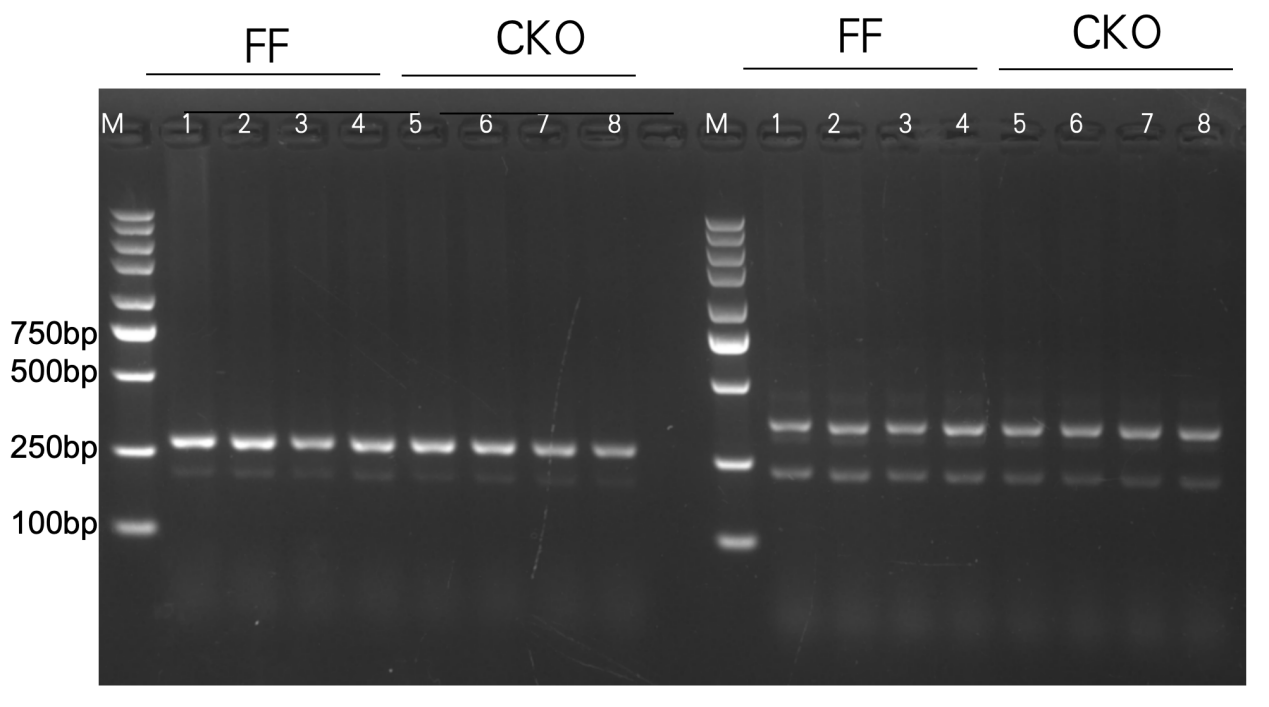


Fig S8 Gel electrophoresis band for gene *Cbl* and *Papola*


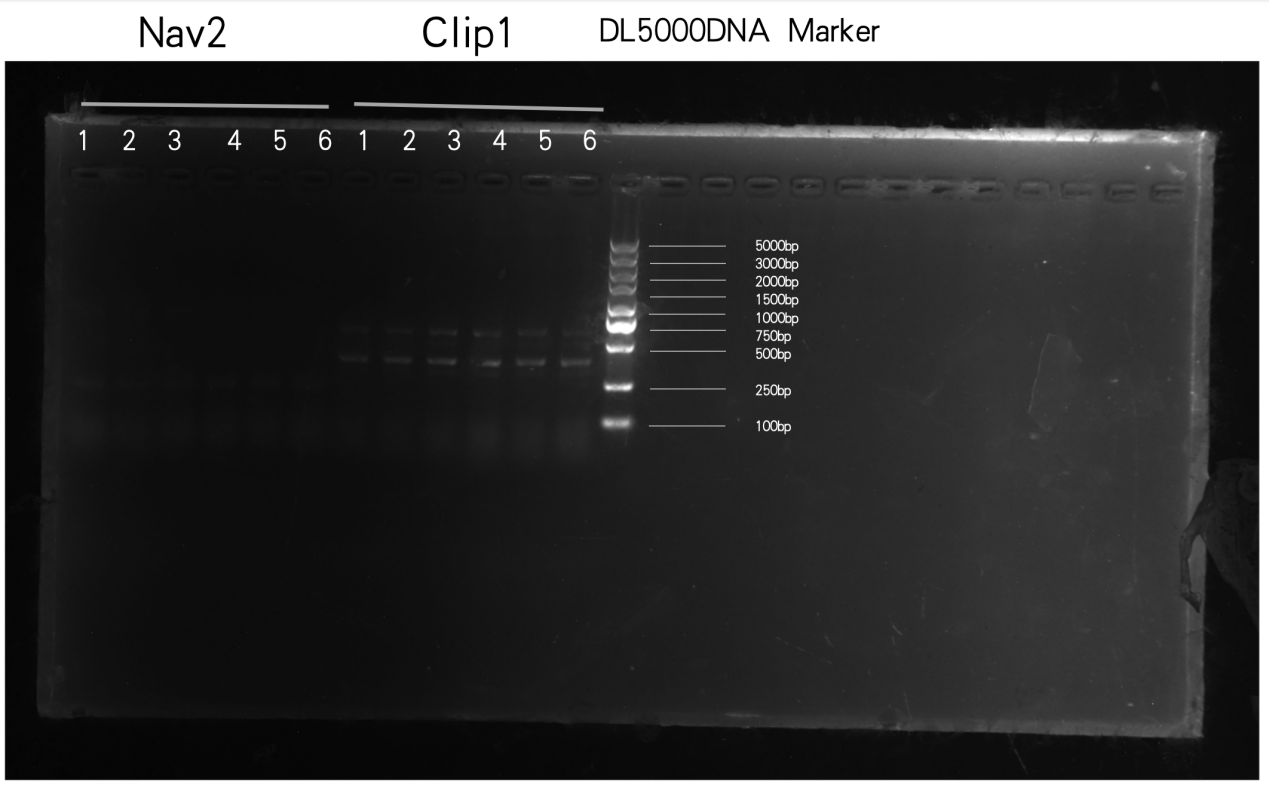


Fig S9 Gel electrophoresis band for gene *Clip1*


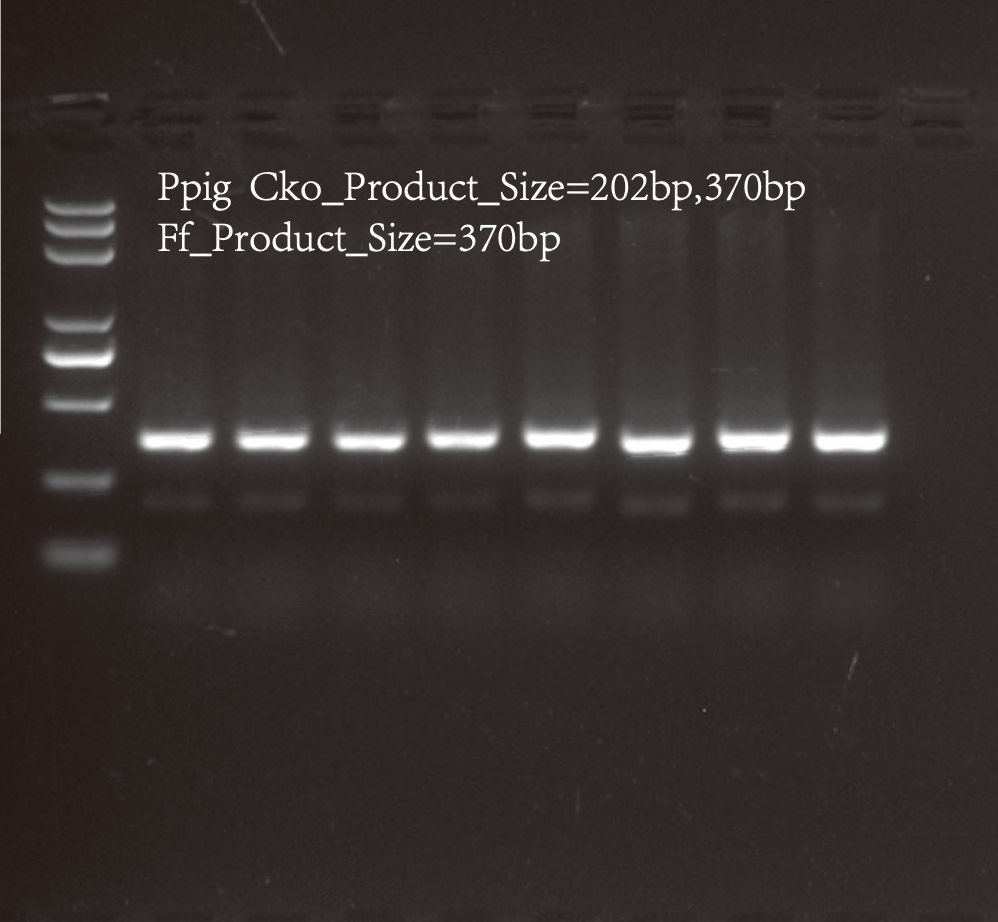


Fig S10 Gel electrophoresis band for gene Ppig


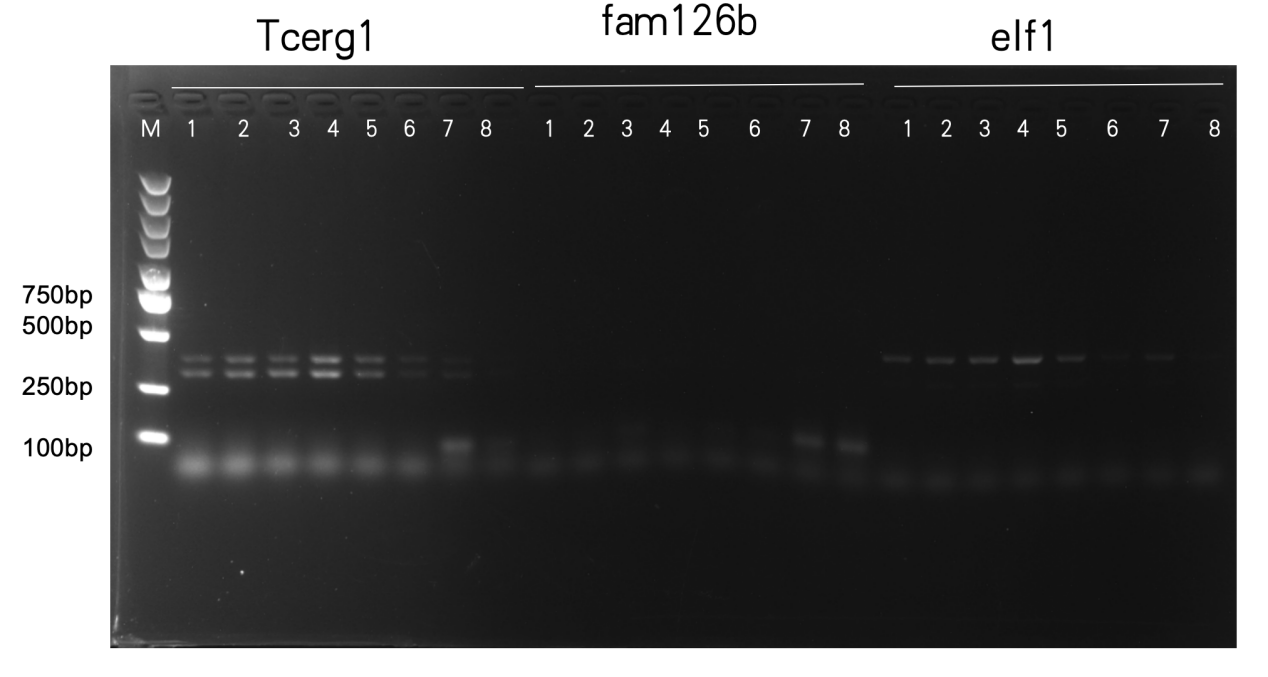


Fig S11 Gel electrophoresis band for gene *Tcerg1*


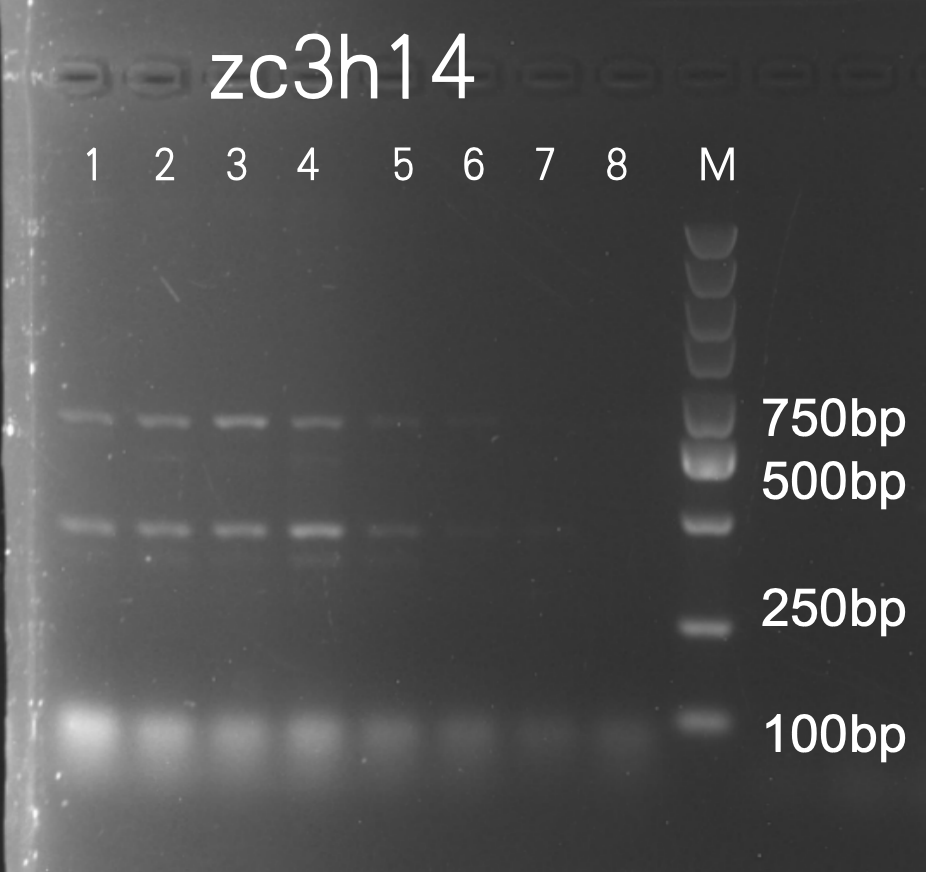


Fig S12 Gel electrophoresis band for gene *Zc3h14*

**Table S14 Global features of the human model**

| No. | Features | Importance | Description |
| --- | --- | --- | --- |
| 1 | length_of_as | 0.00321023 | Length of AS region |
| 2 | DmotifAAAGA | 0.00051058 | Is there this motif in downstream of splicing site: AAAGA (1 for yes, 0 for no) |
| 3 | DmotifTTCTT | 0.00061618 | Is there this motif in downstream of splicing site: TTCTT (1 for yes, 0 for no) |
| 4 | DmotifGTAGG | 0.00266274 | Is there this motif in downstream of splicing site: GTAGG (1 for yes, 0 for no) |
| 5 | DmotifTGAGG | 0.00068823 | Is there this motif in downstream of splicing site: TGAGG (1 for yes, 0 for no) |
| 6 | DmotifTCTTT | 0.00087175 | Is there this motif in downstream of splicing site: TCTTT (1 for yes, 0 for no) |
| 7 | DmotifTAAGT | 0.00069154 | Is there this motif in downstream of splicing site: TAAGT (1 for yes, 0 for no) |
| 8 | DmotifGTGAG | 0.00479803 | Is there this motif in downstream of splicing site: GTGAG (1 for yes, 0 for no) |
| 9 | DmotifGTAAG | 0.00259515 | Is there this motif in downstream of splicing site: GTAAG (1 for yes, 0 for no) |
| 10 | DmotifGTCTG | 0.00071273 | Is there this motif in downstream of splicing site: GTCTG (1 for yes, 0 for no) |
| 11 | DmotifGTTTT | 0.00067621 | Is there this motif in downstream of splicing site: GTTTT (1 for yes, 0 for no) |
| 12 | DmotifTTCTCT | 0.00104417 | Is there this motif in downstream of splicing site: TTCTCT (1 for yes, 0 for no) |
| 13 | DmotifTAAGG | 0.00058586 | Is there this motif in downstream of splicing site: TAAGG (1 for yes, 0 for no) |
| 14 | DmotifTCCTTT | 0.00057234 | Is there this motif in downstream of splicing site: TCCTTT (1 for yes, 0 for no) |
| 15 | DmotifTGTCT | 0.00074713 | Is there this motif in downstream of splicing site: TGTCT (1 for yes, 0 for no) |
| 16 | DmotifTTTCTC | 0.00089086 | Is there this motif in downstream of splicing site: TTTCTC (1 for yes, 0 for no) |
| 17 | DmotifCTTTT | 0.00085476 | Is there this motif in downstream of splicing site: CTTTT (1 for yes, 0 for no) |
| 18 | DmotifTGAGT | 0.00124747 | Is there this motif in downstream of splicing site: TGAGT (1 for yes, 0 for no) |
| 19 | DmotifTAGGT | 0.00133475 | Is there this motif in downstream of splicing site: TAGGT (1 for yes, 0 for no) |
| 20 | DmotifCTTTA | 0.00052679 | Is there this motif in downstream of splicing site: CTTTA (1 for yes, 0 for no) |
| 21 | DmotifTTTAG | 0.00078008 | Is there this motif in downstream of splicing site: TTTAG (1 for yes, 0 for no) |
| 22 | DmotifTGCTT | 0.00070491 | Is there this motif in downstream of splicing site: TGCTT (1 for yes, 0 for no) |
| 23 | DmotifGTGGGT | 0.00094222 | Is there this motif in downstream of splicing site: GTGGGT (1 for yes, 0 for no) |
| 24 | DmotifTCTCC | 0.00068126 | Is there this motif in downstream of splicing site: TCTCC (1 for yes, 0 for no) |
| 25 | DmotifTTTTTC | 0.00139376 | Is there this motif in downstream of splicing site: TTTTTC (1 for yes, 0 for no) |
| 26 | UmotifTTCTT | 0.00054549 | Is there this motif in upstream of splicing site: TTCTT (1 for yes, 0 for no) |
| 27 | UmotifTCTTT | 0.00096571 | Is there this motif in upstream of splicing site: TCTTT (1 for yes, 0 for no) |
| 28 | UmotifTAAGT | 0.00066801 | Is there this motif in upstream of splicing site: TAAGT (1 for yes, 0 for no) |
| 29 | UmotifCTCTG | 0.00061516 | Is there this motif in upstream of splicing site: CTCTG (1 for yes, 0 for no) |
| 30 | UmotifTTTTCC | 0.0007502 | Is there this motif in upstream of splicing site: TTTTCC (1 for yes, 0 for no) |
| 31 | UmotifGTGAG | 0.00550866 | Is there this motif in upstream of splicing site: GTGAG (1 for yes, 0 for no) |
| 32 | UmotifGTAAG | 0.00238317 | Is there this motif in upstream of splicing site: GTAAG (1 for yes, 0 for no) |
| 33 | UmotifCTTCT | 0.00051282 | Is there this motif in upstream of splicing site: CTTCT (1 for yes, 0 for no) |
| 34 | UmotifGTTTT | 0.00054749 | Is there this motif in upstream of splicing site: GTTTT (1 for yes, 0 for no) |
| 35 | UmotifCCTCT | 0.00097555 | Is there this motif in upstream of splicing site: CCTCT (1 for yes, 0 for no) |
| 36 | UmotifTCTCT | 0.00117784 | Is there this motif in upstream of splicing site: TCTCT (1 for yes, 0 for no) |
| 37 | UmotifTGTCT | 0.00059122 | Is there this motif in upstream of splicing site: TGTCT (1 for yes, 0 for no) |
| 38 | UmotifTTCCTT | 0.0005197 | Is there this motif in upstream of splicing site: TTCCTT (1 for yes, 0 for no) |
| 39 | UmotifCTTTT | 0.00080732 | Is there this motif in upstream of splicing site: CTTTT (1 for yes, 0 for no) |
| 40 | UmotifTGAGT | 0.00114328 | Is there this motif in upstream of splicing site: TGAGT (1 for yes, 0 for no) |
| 41 | UmotifCCCCAG | 0.00096588 | Is there this motif in upstream of splicing site: CCCCAG (1 for yes, 0 for no) |
| 42 | UmotifTTTAG | 0.00064961 | Is there this motif in upstream of splicing site: TTTAG (1 for yes, 0 for no) |
| 43 | UmotifTTCTC | 0.00079194 | Is there this motif in upstream of splicing site: TTCTC (1 for yes, 0 for no) |
| 44 | UmotifTGCTT | 0.00068789 | Is there this motif in upstream of splicing site: TGCTT (1 for yes, 0 for no) |
| 45 | allseqGC | 0.00186719 | The GC contant of all sequence |
| 46 | allseqnumberTAA | 0.00085041 | The number of stopdocon TAA in all sequence |
| 47 | allseqnumberTAG | 0.00100172 | The number of stopdocon TAG in all sequence |
| 48 | allseqnumberTGA | 0.00072766 | The number of stopdocon TGA in all sequence |
| 49 | allseqfrequencyA | 0.00094488 | The frequency of A in all sequence |
| 50 | allseqfrequencyAA | 0.00091142 | The frequency of AA in all sequence |
| 51 | allseqfrequencyAAA | 0.00050729 | The frequency of AAA in all sequence |
| 52 | allseqfrequencyAT | 0.00101279 | The frequency of AT in all sequence |
| 53 | allseqfrequencyAC | 0.00053169 | The frequency of AC in all sequence |
| 54 | allseqfrequencyACA | 0.0005611 | The frequency of ACA in all sequence |
| 55 | allseqfrequencyAGG | 0.00052174 | The frequency of AGG in all sequence |
| 56 | allseqfrequencyTT | 0.00054358 | The frequency of TT in all sequence |
| 57 | allseqfrequencyTTT | 0.00056096 | The frequency of TTT in all sequence |
| 58 | allseqfrequencyC | 0.0006169 | The frequency of C in all sequence |
| 59 | allseqfrequencyCA | 0.00096057 | The frequency of CA in all sequence |
| 60 | allseqfrequencyCAA | 0.00084891 | The frequency of CAA in all sequence |
| 61 | allseqfrequencyCC | 0.00064007 | The frequency of CC in all sequence |
| 62 | allseqfrequencyCCT | 0.00065494 | The frequency of CCT in all sequence |
| 63 | allseqfrequencyCCC | 0.00098559 | The frequency of CCC in all sequence |
| 64 | allseqfrequencyCCG | 0.00073578 | The frequency of CCG in all sequence |
| 65 | allseqfrequencyCG | 0.00175652 | The frequency of CG in all sequence |
| 66 | allseqfrequencyCGC | 0.00078686 | The frequency of CGC in all sequence |
| 67 | allseqfrequencyCGG | 0.00133533 | The frequency of CGG in all sequence |
| 68 | allseqfrequencyG | 0.00172969 | The frequency of G in all sequence |
| 69 | allseqfrequencyGA | 0.00059723 | The frequency of GA in all sequence |
| 70 | allseqfrequencyGAA | 0.00061731 | The frequency of GAA in all sequence |
| 71 | allseqfrequencyGTA | 0.00053189 | The frequency of GTA in all sequence |
| 72 | allseqfrequencyGC | 0.00065331 | The frequency of GC in all sequence |
| 73 | allseqfrequencyGCG | 0.00089575 | The frequency of GCG in all sequence |
| 74 | allseqfrequencyGG | 0.00104378 | The frequency of GG in all sequence |
| 75 | allseqfrequencyGGT | 0.00074696 | The frequency of GGT in all sequence |
| 76 | allseqfrequencyGGC | 0.00052334 | The frequency of GGC in all sequence |
| 77 | allseqfrequencyGGG | 0.00212446 | The frequency of GGG in all sequence |
| 78 | allseqdistr50%A | 0.00057426 | The distribution(position/length) of 50%A in all sequence |
| 79 | allseqdistr100%G | 0.00077109 | The distribution(position/length) of 100%G in all sequence |
| 80 | allseqdonerAG | 0.00066435 | Is AG in doner of all sequence (1 for yes, 0 for no) |
| 81 | asseqGC | 0.00273557 | The GC contant of AS region sequence |
| 82 | asseqnumberTAA | 0.00125697 | The number of stopdocon TAA in AS region sequence |
| 83 | asseqnumberTAG | 0.00141324 | The number of stopdocon TAG in AS region sequence |
| 84 | asseqnumberTGA | 0.0011946 | The number of stopdocon TGA in AS region sequence |
| 85 | asseqfrequencyA | 0.00209854 | The frequency of A in AS region sequence |
| 86 | asseqfrequencyAA | 0.00189235 | The frequency of AA in AS region sequence |
| 87 | asseqfrequencyAAA | 0.00068023 | The frequency of AAA in AS region sequence |
| 88 | asseqfrequencyAAT | 0.00066627 | The frequency of AAT in AS region sequence |
| 89 | asseqfrequencyAAC | 0.00065229 | The frequency of AAC in AS region sequence |
| 90 | asseqfrequencyAAG | 0.00130101 | The frequency of AAG in AS region sequence |
| 91 | asseqfrequencyAT | 0.00158739 | The frequency of AT in AS region sequence |
| 92 | asseqfrequencyATA | 0.00056893 | The frequency of ATA in AS region sequence |
| 93 | asseqfrequencyATT | 0.0006019 | The frequency of ATT in AS region sequence |
| 94 | asseqfrequencyATC | 0.00076242 | The frequency of ATC in AS region sequence |
| 95 | asseqfrequencyATG | 0.00077056 | The frequency of ATG in AS region sequence |
| 96 | asseqfrequencyAC | 0.00118974 | The frequency of AC in AS region sequence |
| 97 | asseqfrequencyACA | 0.00096529 | The frequency of ACA in AS region sequence |
| 98 | asseqfrequencyACT | 0.00061111 | The frequency of ACT in AS region sequence |
| 99 | asseqfrequencyACC | 0.00053821 | The frequency of ACC in AS region sequence |
| 100 | asseqfrequencyACG | 0.00055502 | The frequency of ACG in AS region sequence |
| 101 | asseqfrequencyAG | 0.0028336 | The frequency of AG in AS region sequence |
| 102 | asseqfrequencyAGA | 0.00169715 | The frequency of AGA in AS region sequence |
| 103 | asseqfrequencyAGT | 0.00094007 | The frequency of AGT in AS region sequence |
| 104 | asseqfrequencyAGC | 0.00098379 | The frequency of AGC in AS region sequence |
| 105 | asseqfrequencyAGG | 0.0015495 | The frequency of AGG in AS region sequence |
| 106 | asseqfrequencyT | 0.00200717 | The frequency of T in AS region sequence |
| 107 | asseqfrequencyTA | 0.00125172 | The frequency of TA in AS region sequence |
| 108 | asseqfrequencyTAA | 0.00063381 | The frequency of TAA in AS region sequence |
| 109 | asseqfrequencyTAT | 0.00056674 | The frequency of TAT in AS region sequence |
| 110 | asseqfrequencyTAC | 0.00060683 | The frequency of TAC in AS region sequence |
| 111 | asseqfrequencyTAG | 0.00067511 | The frequency of TAG in AS region sequence |
| 112 | asseqfrequencyTT | 0.00147658 | The frequency of TT in AS region sequence |
| 113 | asseqfrequencyTTA | 0.00055822 | The frequency of TTA in AS region sequence |
| 114 | asseqfrequencyTTT | 0.0011774 | The frequency of TTT in AS region sequence |
| 115 | asseqfrequencyTTC | 0.00084713 | The frequency of TTC in AS region sequence |
| 116 | asseqfrequencyTTG | 0.00057696 | The frequency of TTG in AS region sequence |
| 117 | asseqfrequencyTC | 0.00113531 | The frequency of TC in AS region sequence |
| 118 | asseqfrequencyTCA | 0.0012888 | The frequency of TCA in AS region sequence |
| 119 | asseqfrequencyTCT | 0.000873 | The frequency of TCT in AS region sequence |
| 120 | asseqfrequencyTCC | 0.0008606 | The frequency of TCC in AS region sequence |
| 121 | asseqfrequencyTCG | 0.00052793 | The frequency of TCG in AS region sequence |
| 122 | asseqfrequencyTG | 0.00084508 | The frequency of TG in AS region sequence |
| 123 | asseqfrequencyTGA | 0.00085939 | The frequency of TGA in AS region sequence |
| 124 | asseqfrequencyTGT | 0.00097791 | The frequency of TGT in AS region sequence |
| 125 | asseqfrequencyTGC | 0.00076763 | The frequency of TGC in AS region sequence |
| 126 | asseqfrequencyTGG | 0.00084692 | The frequency of TGG in AS region sequence |
| 127 | asseqfrequencyC | 0.00157183 | The frequency of C in AS region sequence |
| 128 | asseqfrequencyCA | 0.00265721 | The frequency of CA in AS region sequence |
| 129 | asseqfrequencyCAA | 0.00124177 | The frequency of CAA in AS region sequence |
| 130 | asseqfrequencyCAT | 0.00069481 | The frequency of CAT in AS region sequence |
| 131 | asseqfrequencyCAC | 0.00064456 | The frequency of CAC in AS region sequence |
| 132 | asseqfrequencyCAG | 0.00254851 | The frequency of CAG in AS region sequence |
| 133 | asseqfrequencyCT | 0.00102412 | The frequency of CT in AS region sequence |
| 134 | asseqfrequencyCTT | 0.00082232 | The frequency of CTT in AS region sequence |
| 135 | asseqfrequencyCTC | 0.00065311 | The frequency of CTC in AS region sequence |
| 136 | asseqfrequencyCTG | 0.00069029 | The frequency of CTG in AS region sequence |
| 137 | asseqfrequencyCC | 0.00125255 | The frequency of CC in AS region sequence |
| 138 | asseqfrequencyCCA | 0.00066105 | The frequency of CCA in AS region sequence |
| 139 | asseqfrequencyCCT | 0.00098925 | The frequency of CCT in AS region sequence |
| 140 | asseqfrequencyCCC | 0.00127424 | The frequency of CCC in AS region sequence |
| 141 | asseqfrequencyCCG | 0.00081053 | The frequency of CCG in AS region sequence |
| 142 | asseqfrequencyCG | 0.00158243 | The frequency of CG in AS region sequence |
| 143 | asseqfrequencyCGA | 0.00062279 | The frequency of CGA in AS region sequence |
| 144 | asseqfrequencyCGC | 0.00062939 | The frequency of CGC in AS region sequence |
| 145 | asseqfrequencyCGG | 0.00114093 | The frequency of CGG in AS region sequence |
| 146 | asseqfrequencyG | 0.00352234 | The frequency of G in AS region sequence |
| 147 | asseqfrequencyGA | 0.00239326 | The frequency of GA in AS region sequence |
| 148 | asseqfrequencyGAA | 0.00104029 | The frequency of GAA in AS region sequence |
| 149 | asseqfrequencyGAT | 0.00075181 | The frequency of GAT in AS region sequence |
| 150 | asseqfrequencyGAC | 0.00066078 | The frequency of GAC in AS region sequence |
| 151 | asseqfrequencyGAG | 0.00106982 | The frequency of GAG in AS region sequence |
| 152 | asseqfrequencyGT | 0.00298577 | The frequency of GT in AS region sequence |
| 153 | asseqfrequencyGTA | 0.00115602 | The frequency of GTA in AS region sequence |
| 154 | asseqfrequencyGTT | 0.00055317 | The frequency of GTT in AS region sequence |
| 155 | asseqfrequencyGTC | 0.00056694 | The frequency of GTC in AS region sequence |
| 156 | asseqfrequencyGTG | 0.00132808 | The frequency of GTG in AS region sequence |
| 157 | asseqfrequencyGC | 0.00095184 | The frequency of GC in AS region sequence |
| 158 | asseqfrequencyGCA | 0.00073113 | The frequency of GCA in AS region sequence |
| 159 | asseqfrequencyGCT | 0.00055435 | The frequency of GCT in AS region sequence |
| 160 | asseqfrequencyGCC | 0.00066351 | The frequency of GCC in AS region sequence |
| 161 | asseqfrequencyGCG | 0.00093908 | The frequency of GCG in AS region sequence |
| 162 | asseqfrequencyGG | 0.00222462 | The frequency of GG in AS region sequence |
| 163 | asseqfrequencyGGA | 0.00122948 | The frequency of GGA in AS region sequence |
| 164 | asseqfrequencyGGT | 0.00081454 | The frequency of GGT in AS region sequence |
| 165 | asseqfrequencyGGC | 0.00117968 | The frequency of GGC in AS region sequence |
| 166 | asseqfrequencyGGG | 0.00267595 | The frequency of GGG in AS region sequence |
| 167 | asseqdistr1%A | 0.00411731 | The distribution(position/length) of 1%A in AS region sequence |
| 168 | asseqdistr25%A | 0.00606579 | The distribution(position/length) of 25%A in AS region sequence |
| 169 | asseqdistr50%A | 0.00183051 | The distribution(position/length) of 50%A in AS region sequence |
| 170 | asseqdistr75%A | 0.00264277 | The distribution(position/length) of 75%A in AS region sequence |
| 171 | asseqdistr100%A | 0.00284182 | The distribution(position/length) of 100%A in AS region sequence |
| 172 | asseqdistr1%T | 0.00188463 | The distribution(position/length) of 1%T in AS region sequence |
| 173 | asseqdistr25%T | 0.00151007 | The distribution(position/length) of 25%T in AS region sequence |
| 174 | asseqdistr50%T | 0.00237935 | The distribution(position/length) of 50%T in AS region sequence |
| 175 | asseqdistr75%T | 0.00290814 | The distribution(position/length) of 75%T in AS region sequence |
| 176 | asseqdistr100%T | 0.00607481 | The distribution(position/length) of 100%T in AS region sequence |
| 177 | asseqdistr1%C | 0.00244276 | The distribution(position/length) of 1%C in AS region sequence |
| 178 | asseqdistr25%C | 0.00142548 | The distribution(position/length) of 25%C in AS region sequence |
| 179 | asseqdistr50%C | 0.00135641 | The distribution(position/length) of 50%C in AS region sequence |
| 180 | asseqdistr75%C | 0.00293903 | The distribution(position/length) of 75%C in AS region sequence |
| 181 | asseqdistr100%C | 0.00404454 | The distribution(position/length) of 100%C in AS region sequence |
| 182 | asseqdistr1%G | 0.00910389 | The distribution(position/length) of 1%G in AS region sequence |
| 183 | asseqdistr25%G | 0.00879149 | The distribution(position/length) of 25%G in AS region sequence |
| 184 | asseqdistr50%G | 0.004301 | The distribution(position/length) of 50%G in AS region sequence |
| 185 | asseqdistr75%G | 0.00253368 | The distribution(position/length) of 75%G in AS region sequence |
| 186 | asseqdistr100%G | 0.00106038 | The distribution(position/length) of 100%G in AS region sequence |
| 187 | asseqdonerGT | 0.00077499 | Is there GT in doner of AS region sequence (1 for yes, 0 for no) |
| 188 | asseqacceptorGT | 0.0232506 | Is there GT in acceptor of AS region sequence (1 for yes, 0 for no) |
| 189 | asseqdonerGC | 0.00053073 | Is there GC in doner of AS region sequence (1 for yes, 0 for no) |
| 190 | asseqacceptorGC | 0.00103237 | Is there GC in acceptor of AS region sequence (1 for yes, 0 for no) |
| 191 | asseqdonerAT | 0.00087271 | Is there AT in doner of AS region sequence (1 for yes, 0 for no) |
| 192 | asseqacceptorAT | 0.0011296 | Is there AT in acceptor of AS region sequence (1 for yes, 0 for no) |
| 193 | asseqdonerAG | 0.00848124 | Is there AG in doner of AS region sequence (1 for yes, 0 for no) |
| 194 | asseqacceptorAG | 0.00450759 | Is there AG in acceptor of AS region sequence (1 for yes, 0 for no) |
| 195 | asseqacceptorAC | 0.00057082 | Is there AC in acceptor of AS region sequence (1 for yes, 0 for no) |
| 196 | updistr100%A | 0.00050752 | The distribution(position/length) of 100%A in upstream sequence |
| 197 | updistr100%G | 0.00156791 | The distribution(position/length) of 100%G in upstream sequence |
| 198 | updonerAG | 0.00112393 | Is AG in doner of upstream sequence (1 for yes, 0 for no) |
| 199 | downGC | 0.00129222 | The GC contant of downstream sequence |
| 200 | downfrequencyAT | 0.00075052 | The frequency of AT in downstream sequence |
| 201 | downfrequencyT | 0.00055035 | The frequency of T in downstream sequence |
| 202 | downfrequencyCCG | 0.00058042 | The frequency of CCG in downstream sequence |
| 203 | downfrequencyCG | 0.00116404 | The frequency of CG in downstream sequence |
| 204 | downfrequencyCGC | 0.00057317 | The frequency of CGC in downstream sequence |
| 205 | downfrequencyCGG | 0.00059605 | The frequency of CGG in downstream sequence |
| 206 | downfrequencyG | 0.00052572 | The frequency of G in downstream sequence |
| 207 | downdistr100%G | 0.00063384 | The distribution(position/length) of 100%G in downstream sequence |
| 208 | downdonerAG | 0.00062139 | Is there AG in doner of downstream sequence (1 for yes, 0 for no) |
| 209 | up30as30if%3 | 0.00474754 | Whethere the length of upstream30 + AS 30bp sequence divisible by three |
| 210 | up30as30GC | 0.00097856 | The GC contant of upstream30AS 30bp + AS 30bp sequence |
| 211 | up30as30frequencyAT | 0.00059268 | The frequency of AT in upstream 30 bp + AS 30bp sequence |
| 212 | up30as30frequencyAGG | 0.00050739 | The frequency of AGG in upstream 30 bp + AS 30bp sequence |
| 213 | up30as30frequencyTAA | 0.00051832 | The frequency of TAA in upstream 30 bp + AS 30bp sequence |
| 214 | up30as30frequencyCA | 0.00054439 | The frequency of CA in upstream 30 bp + AS 30bp sequence |
| 215 | up30as30frequencyCG | 0.0005249 | The frequency of CG in upstream 30 bp + AS 30bp sequence |
| 216 | up30as30frequencyG | 0.00090055 | The frequency of G in upstream 30 bp + AS 30bp sequence |
| 217 | up30as30frequencyGT | 0.00100878 | The frequency of GT in upstream 30 bp + AS 30bp sequence |
| 218 | up30as30frequencyGTA | 0.00186856 | The frequency of GTA in upstream 30 bp + AS 30bp sequence |
| 219 | up30as30frequencyGTG | 0.00104536 | The frequency of GTG in upstream 30 bp + AS 30bp sequence |
| 220 | up30as30frequencyGG | 0.00068261 | The frequency of GG in upstream 30 bp + AS 30bp sequence |
| 221 | up30as30frequencyGGT | 0.00203348 | The frequency of GGT in upstream 30 bp + AS 30bp sequence |
| 222 | up30as30frequencyGGG | 0.00101225 | The frequency of GGG in upstream 30 bp + AS 30bp sequence |
| 223 | up30as30distr50%A | 0.00057693 | The distribution(position/length) of 50%A in upstream 30 bp + AS 30bp sequence |
| 224 | up30as30distr75%A | 0.00065496 | The distribution(position/length) of 75%A in upstream 30 bp + AS 30bp sequence |
| 225 | up30as30distr100%A | 0.00079364 | The distribution(position/length) of 100%A in upstream 30 bp + AS 30bp sequence |
| 226 | up30as30distr75%T | 0.00076725 | The distribution(position/length) of 75%T in upstream 30 bp + AS 30bp sequence |
| 227 | up30as30distr100%T | 0.00055656 | The distribution(position/length) of 100%T in upstream 30 bp + AS 30bp sequence |
| 228 | up30as30distr50%G | 0.00061018 | The distribution(position/length) of 50%G in upstream 30 bp + AS 30bp sequence |
| 229 | up30as30distr75%G | 0.00056901 | The distribution(position/length) of 75%G in upstream 30 bp + AS 30bp sequence |
| 230 | up30as30distr100%G | 0.00069336 | The distribution(position/length) of 100%G in upstream 30 bp + AS 30bp sequence |
| 231 | up30as30donerGT | 0.00055622 | Is there GT in doner of upstream 30 bp + AS 30bp sequence (1 for yes, 0 for no) |
| 232 | up30as30donerAG | 0.00164825 | Is there AG in doner of upstream 30 bp + AS 30bp sequence (1 for yes, 0 for no) |
| 233 | up30down30GC | 0.00052924 | The GC contant of upstream 30 + downstream 30GC sequence |
| 234 | as30down30if%3 | 0.00650359 | Whethere the length of AS 30bp + downstream 30bp sequence divisible by three |
| 235 | as30down30GC | 0.00137061 | The GC contant of AS 30bp + downstream 30bp sequence |
| 236 | as30down30frequencyA | 0.00248632 | The frequency of A in AS 30bp + downstream 30bp sequence |
| 237 | as30down30frequencyAA | 0.00118563 | The frequency of AA in AS 30bp + downstream 30bp sequence |
| 238 | as30down30frequencyAAA | 0.00069696 | The frequency of AAA in AS 30bp + downstream 30bp sequence |
| 239 | as30down30frequencyAAT | 0.00051676 | The frequency of AAT in AS 30bp + downstream 30bp sequence |
| 240 | as30down30frequencyAAG | 0.00085099 | The frequency of AAG in AS 30bp + downstream 30bp sequence |
| 241 | as30down30frequencyAT | 0.00061518 | The frequency of AT in AS 30bp + downstream 30bp sequence |
| 242 | as30down30frequencyAG | 0.0007348 | The frequency of AG in AS 30bp + downstream 30bp sequence |
| 243 | as30down30frequencyT | 0.00096321 | The frequency of T in AS 30bp + downstream 30bp sequence |
| 244 | as30down30frequencyTT | 0.00078277 | The frequency of TT in AS 30bp + downstream 30bp sequence |
| 245 | as30down30frequencyTTT | 0.00072603 | The frequency of TTT in AS 30bp + downstream 30bp sequence |
| 246 | as30down30frequencyTTC | 0.00069752 | The frequency of TTC in AS 30bp + downstream 30bp sequence |
| 247 | as30down30frequencyTC | 0.00114611 | The frequency of TC in AS 30bp + downstream 30bp sequence |
| 248 | as30down30frequencyTCT | 0.00088733 | The frequency of TCT in AS 30bp + downstream 30bp sequence |
| 249 | as30down30frequencyTCC | 0.00068028 | The frequency of TCC in AS 30bp + downstream 30bp sequence |
| 250 | as30down30frequencyTGT | 0.00050917 | The frequency of TGT in AS 30bp + downstream 30bp sequence |
| 251 | as30down30frequencyC | 0.0015108 | The frequency of C in AS 30bp + downstream 30bp sequence |
| 252 | as30down30frequencyCAA | 0.00057239 | The frequency of CAA in AS 30bp + downstream 30bp sequence |
| 253 | as30down30frequencyCT | 0.00100102 | The frequency of CT in AS 30bp + downstream 30bp sequence |
| 254 | as30down30frequencyCTT | 0.00062108 | The frequency of CTT in AS 30bp + downstream 30bp sequence |
| 255 | as30down30frequencyCTC | 0.00068795 | The frequency of CTC in AS 30bp + downstream 30bp sequence |
| 256 | as30down30frequencyCC | 0.00124744 | The frequency of CC in AS 30bp + downstream 30bp sequence |
| 257 | as30down30frequencyCCT | 0.00069083 | The frequency of CCT in AS 30bp + downstream 30bp sequence |
| 258 | as30down30frequencyCCC | 0.00112537 | The frequency of CCC in AS 30bp + downstream 30bp sequence |
| 259 | as30down30frequencyCG | 0.00112878 | The frequency of CG in AS 30bp + downstream 30bp sequence |
| 260 | as30down30frequencyCGC | 0.00057624 | The frequency of CGC in AS 30bp + downstream 30bp sequence |
| 261 | as30down30frequencyCGG | 0.00063691 | The frequency of CGG in AS 30bp + downstream 30bp sequence |
| 262 | as30down30frequencyG | 0.00119789 | The frequency of G in AS 30bp + downstream 30bp sequence |
| 263 | as30down30frequencyGA | 0.0007453 | The frequency of GA in AS 30bp + downstream 30bp sequence |
| 264 | as30down30frequencyGAA | 0.000782 | The frequency of GAA in AS 30bp + downstream 30bp sequence |
| 265 | as30down30frequencyGAG | 0.00055691 | The frequency of GAG in AS 30bp + downstream 30bp sequence |
| 266 | as30down30frequencyGC | 0.00059211 | The frequency of GC in AS 30bp + downstream 30bp sequence |
| 267 | as30down30frequencyGCG | 0.00084407 | The frequency of GCG in AS 30bp + downstream 30bp sequence |
| 268 | as30down30frequencyGG | 0.00070159 | The frequency of GG in AS 30bp + downstream 30bp sequence |
| 269 | as30down30frequencyGGA | 0.00054978 | The frequency of GGA in AS 30bp + downstream 30bp sequence |
| 270 | as30down30frequencyGGC | 0.0005576 | The frequency of GGC in AS 30bp + downstream 30bp sequence |
| 271 | as30down30frequencyGGG | 0.00068282 | The frequency of GGG in AS 30bp + downstream 30bp sequence |
| 272 | as30down30distr1%A | 0.00066735 | The distribution(position/length) of 1%A in AS 30bp + downstream 30bp sequence |
| 273 | as30down30distr25%A | 0.00076062 | The distribution(position/length) of 25%A in AS 30bp + downstream 30bp sequence |
| 274 | as30down30distr50%A | 0.0007277 | The distribution(position/length) of 50%A in AS 30bp + downstream 30bp sequence |
| 275 | as30down30distr75%A | 0.00051167 | The distribution(position/length) of 75%A in AS 30bp + downstream 30bp sequence |
| 276 | as30down30distr1%T | 0.00071621 | The distribution(position/length) of 1%T in AS 30bp + downstream 30bp sequence |
| 277 | as30down30distr50%T | 0.00263898 | The distribution(position/length) of 50%T in AS 30bp + downstream 30bp sequence |
| 278 | as30down30distr75%T | 0.0010547 | The distribution(position/length) of 75%T in AS 30bp + downstream 30bp sequence |
| 279 | as30down30distr1%C | 0.00056924 | The distribution(position/length) of 1%C in AS 30bp + downstream 30bp sequence |
| 280 | as30down30distr25%C | 0.00051178 | The distribution(position/length) of 25%C in AS 30bp + downstream 30bp sequence |
| 281 | as30down30distr50%C | 0.00052881 | The distribution(position/length) of 50%C in AS 30bp + downstream 30bp sequence |
| 282 | as30down30distr1%G | 0.00118134 | The distribution(position/length) of 1%G in AS 30bp + downstream 30bp sequence |
| 283 | as30down30distr25%G | 0.00084032 | The distribution(position/length) of 25%G in AS 30bp + downstream 30bp sequence |
| 284 | as30down30distr50%G | 0.00153808 | The distribution(position/length) of 50%G in AS 30bp + downstream 30bp sequence |
| 285 | as30down30distr75%G | 0.00058458 | The distribution(position/length) of 75%G in AS 30bp + downstream 30bp sequence |
| 286 | as30down30acceptorGT | 0.00180244 | Is there GT in acceptor of AS 30bp + downstream 30bp sequence (1 for yes, 0 for no) |
| 287 | up50as50if%3 | 0.00217258 | Whethere the length of upstream 50bp + AS 50bp sequence divisible by three |
| 288 | up50as50GC | 0.00161372 | The GC contant of upstream 50bp + AS 50bp sequence |
| 289 | up50as50numberTGA | 0.00051969 | The numberof stopdocon TGA in upstream 50bp + AS 50bp sequence |
| 290 | up50as50frequencyA | 0.00073539 | The frequency of A in upstream 50bp + AS 50bp sequence |
| 291 | up50as50frequencyAA | 0.0005253 | The frequency of AA in upstream 50bp + AS 50bp sequence |
| 292 | up50as50frequencyAAA | 0.00054986 | The frequency of AAA in upstream 50bp + AS 50bp sequence |
| 293 | up50as50frequencyAT | 0.00050509 | The frequency of AT in upstream 50bp + AS 50bp sequence |
| 294 | up50as50frequencyAGG | 0.00053668 | The frequency of AGG in upstream 50bp + AS 50bp sequence |
| 295 | up50as50frequencyCA | 0.00063607 | The frequency of CA in upstream 50bp + AS 50bp sequence |
| 296 | up50as50frequencyCC | 0.00054102 | The frequency of CC in upstream 50bp + AS 50bp sequence |
| 297 | up50as50frequencyCCT | 0.0005109 | The frequency of CCT in upstream 50bp + AS 50bp sequence |
| 298 | up50as50frequencyCCC | 0.00054381 | The frequency of CCC in upstream 50bp + AS 50bp sequence |
| 299 | up50as50frequencyCG | 0.0006102 | The frequency of CG in upstream 50bp + AS 50bp sequence |
| 300 | up50as50frequencyG | 0.0008402 | The frequency of G in upstream 50bp + AS 50bp sequence |
| 301 | up50as50frequencyGT | 0.00064936 | The frequency of GT in upstream 50bp + AS 50bp sequence |
| 302 | up50as50frequencyGTA | 0.00108651 | The frequency of GTA in upstream 50bp + AS 50bp sequence |
| 303 | up50as50frequencyGTG | 0.00062282 | The frequency of GTG in upstream 50bp + AS 50bp sequence |
| 304 | up50as50frequencyGG | 0.00088383 | The frequency of GG in upstream 50bp + AS 50bp sequence |
| 305 | up50as50frequencyGGT | 0.00109505 | The frequency of GGT in upstream 50bp + AS 50bp sequence |
| 306 | up50as50frequencyGGG | 0.00142172 | The frequency of GGG in upstream 50bp + AS 50bp sequence |
| 307 | up50as50distr25%A | 0.00052063 | The distribution(position/length) of 25%A in upstream 50bp + AS 50bp sequence |
| 308 | up50as50distr50%A | 0.00074426 | The distribution(position/length) of 50%A in upstream 50bp + AS 50bp sequence |
| 309 | up50as50distr75%A | 0.00075071 | The distribution(position/length) of 75%A in upstream 50bp + AS 50bp sequence |
| 310 | up50as50distr100%A | 0.00089748 | The distribution(position/length) of 100%A in upstream 50bp + AS 50bp sequence |
| 311 | up50as50distr50%T | 0.00061052 | The distribution(position/length) of 50%T in upstream 50bp + AS 50bp sequence |
| 312 | up50as50distr75%T | 0.00067725 | The distribution(position/length) of 75%T in upstream 50bp + AS 50bp sequence |
| 313 | up50as50distr100%T | 0.00060157 | The distribution(position/length) of 100%T in upstream 50bp + AS 50bp sequence |
| 314 | up50as50distr100%C | 0.00062694 | The distribution(position/length) of 100%C in upstream 50bp + AS 50bp sequence |
| 315 | up50as50distr25%G | 0.00051214 | The distribution(position/length) of 25%G in upstream 50bp + AS 50bp sequence |
| 316 | up50as50distr50%G | 0.00074642 | The distribution(position/length) of 50%G in upstream 50bp + AS 50bp sequence |
| 317 | up50as50distr75%G | 0.00067369 | The distribution(position/length) of 75%G in upstream 50bp + AS 50bp sequence |
| 318 | up50as50distr100%G | 0.0008374 | The distribution(position/length) of 100%G in upstream 50bp + AS 50bp sequence |
| 319 | up50as50donerGT | 0.00059529 | Is there GT in doner of upstream 50bp + AS 50bp sequence (1 for yes, 0 for no) |
| 320 | up50as50donerAG | 0.00252162 | Is there AG in doner of upstream 50bp + AS 50bp sequence (1 for yes, 0 for no) |
| 321 | up50down50GC | 0.00054293 | The GC contant upstream 50bp + + downstream 50bp sequence |
| 322 | up50down50distr100%G | 0.00068183 | The distribution(position/length) of 100%G in upstream 50bp + downstream 50bp sequence |
| 323 | up50down50donerAG | 0.00064568 | Is there AG in doner of upstream 50bp + downstream 50bp sequence (1 for yes, 0 for no) |
| 324 | as50down50if%3 | 0.00219184 | Whethere the length of AS 50bp + downstream 50bp sequence divisible by three |
| 325 | as50down50GC | 0.00183917 | The GC contant of GC in AS 50bp + downstream 50bp sequence |
| 326 | as50down50numberTAA | 0.00050581 | The number of stopdocon TAA in AS 50bp + downstream 50bp sequence |
| 327 | as50down50numberTGA | 0.0006772 | The number of stopdocon TGA in AS 50bp + downstream 50bp sequence |
| 328 | as50down50frequencyA | 0.00170553 | The frequency of A in AS 50bp + downstream 50bp sequence |
| 329 | as50down50frequencyAA | 0.00095836 | The frequency of AA in AS 50bp + downstream 50bp sequence |
| 330 | as50down50frequencyAAA | 0.00094627 | The frequency of AAA in AS 50bp + downstream 50bp sequence |
| 331 | as50down50frequencyAAG | 0.00079716 | The frequency of AAG in AS 50bp + downstream 50bp sequence |
| 332 | as50down50frequencyAT | 0.00081654 | The frequency of AT in AS 50bp + downstream 50bp sequence |
| 333 | as50down50frequencyATT | 0.00055137 | The frequency of ATT in AS 50bp + downstream 50bp sequence |
| 334 | as50down50frequencyAG | 0.00058994 | The frequency of AG in AS 50bp + downstream 50bp sequence |
| 335 | as50down50frequencyAGA | 0.00052927 | The frequency of AGA in AS 50bp + downstream 50bp sequence |
| 336 | as50down50frequencyT | 0.00079415 | The frequency of T in AS 50bp + downstream 50bp sequence |
| 337 | as50down50frequencyTT | 0.00059693 | The frequency of TT in AS 50bp + downstream 50bp sequence |
| 338 | as50down50frequencyTTT | 0.00068869 | The frequency of TTT in AS 50bp + downstream 50bp sequence |
| 339 | as50down50frequencyTTC | 0.00052754 | The frequency of TTC in AS 50bp + downstream 50bp sequence |
| 340 | as50down50frequencyTC | 0.00062893 | The frequency of TC in AS 50bp + downstream 50bp sequence |
| 341 | as50down50frequencyTCT | 0.00061056 | The frequency of TCT in AS 50bp + downstream 50bp sequence |
| 342 | as50down50frequencyTCC | 0.00058486 | The frequency of TCC in AS 50bp + downstream 50bp sequence |
| 343 | as50down50frequencyC | 0.00108165 | The frequency of C in AS 50bp + downstream 50bp sequence |
| 344 | as50down50frequencyCAA | 0.00084815 | The frequency of CAA in AS 50bp + downstream 50bp sequence |
| 345 | as50down50frequencyCT | 0.000901 | The frequency of CT in AS 50bp + downstream 50bp sequence |
| 346 | as50down50frequencyCTT | 0.000519 | The frequency of CTT in AS 50bp + downstream 50bp sequence |
| 347 | as50down50frequencyCTC | 0.00057286 | The frequency of CTC in AS 50bp + downstream 50bp sequence |
| 348 | as50down50frequencyCC | 0.0008822 | The frequency of CC in AS 50bp + downstream 50bp sequence |
| 349 | as50down50frequencyCCT | 0.00066117 | The frequency of CCT in AS 50bp + downstream 50bp sequence |
| 350 | as50down50frequencyCCC | 0.00123427 | The frequency of CCC in AS 50bp + downstream 50bp sequence |
| 351 | as50down50frequencyCCG | 0.00064359 | The frequency of CCG in AS 50bp + downstream 50bp sequence |
| 352 | as50down50frequencyCG | 0.00149152 | The frequency of CG in AS 50bp + downstream 50bp sequence |
| 353 | as50down50frequencyCGC | 0.00062161 | The frequency of CGC in AS 50bp + downstream 50bp sequence |
| 354 | as50down50frequencyCGG | 0.0009457 | The frequency of CGG in AS 50bp + downstream 50bp sequence |
| 355 | as50down50frequencyG | 0.00090804 | The frequency of G in AS 50bp + downstream 50bp sequence |
| 356 | as50down50frequencyGA | 0.0008211 | The frequency of GA in AS 50bp + downstream 50bp sequence |
| 357 | as50down50frequencyGAA | 0.00077229 | The frequency of GAA in AS 50bp + downstream 50bp sequence |
| 358 | as50down50frequencyGC | 0.00057992 | The frequency of GC in AS 50bp + downstream 50bp sequence |
| 359 | as50down50frequencyGCG | 0.00090251 | The frequency of GCG in AS 50bp + downstream 50bp sequence |
| 360 | as50down50frequencyGG | 0.00058702 | The frequency of GG in AS 50bp + downstream 50bp sequence |
| 361 | as50down50frequencyGGG | 0.00083036 | The frequency of GGG in AS 50bp + downstream 50bp sequence |
| 362 | as50down50distr1%A | 0.00076284 | The distribution(position/length) of 1%A in AS 50bp + downstream 50bp sequence |
| 363 | as50down50distr50%A | 0.00066968 | The distribution(position/length) of 50%A in AS 50bp + downstream 50bp sequence |
| 364 | as50down50distr1%T | 0.00078609 | The distribution(position/length) of 1%T in AS 50bp + downstream 50bp sequence |
| 365 | as50down50distr50%T | 0.00128706 | The distribution(position/length) of 50%T in AS 50bp + downstream 50bp sequence |
| 366 | as50down50distr75%T | 0.00079896 | The distribution(position/length) of 75%T in AS 50bp + downstream 50bp sequence |
| 367 | as50down50distr1%C | 0.00070529 | The distribution(position/length) of 1%C in AS 50bp + downstream 50bp sequence |
| 368 | as50down50distr1%G | 0.00118752 | The distribution(position/length) of 1%G in AS 50bp + downstream 50bp sequence |
| 369 | as50down50distr25%G | 0.00074848 | The distribution(position/length) of 25%G in AS 50bp + downstream 50bp sequence |
| 370 | as50down50distr50%G | 0.00079151 | The distribution(position/length) of 50%G in AS 50bp + downstream 50bp sequence |
| 371 | as50down50distr100%G | 0.00058932 | The distribution(position/length) of 100%G in AS 50bp + downstream 50bp sequence |
| 372 | as50down50acceptorGT | 0.00320036 | Is there GT in acceptor of AS 50bp + downstream 50bp sequence (1 for yes, 0 for no) |
| 373 | as50down50donerAG | 0.00065088 | Is there AG in doner of AS 50bp + downstream 50bp sequence (1 for yes, 0 for no) |
| 374 | as50down50acceptorAG | 0.00065056 | Is there AG in acceptor of AS 50bp + downstream 50bp sequence (1 for yes, 0 for no) |
| 375 | down20bpGC | 0.00064652 | The GC contant of downstream 20bp sequence |
| 376 | down20bpfrequencyCG | 0.00064071 | The frequency of CG in downstream 20bp sequence |

**Table S15 Global features of the *Arabidopsis thaliana* model**

| No. | Features | Importance | Description |
| --- | --- | --- | --- |
| 1 | length_of_as | 0.0031287 | Length of AS region |
| 2 | DmotifTTCTT | 0.00090826 | Is there this motif in downstream of splicing site: TTCTT (1 for yes, 0 for no) |
| 3 | DmotifTAACT | 0.0005252 | Is there this motif in downstream of splicing site: TAACT (1 for yes, 0 for no) |
| 4 | DmotifTCTTT | 0.00086944 | Is there this motif in downstream of splicing site: TCTTT (1 for yes, 0 for no) |
| 5 | DmotifTCTGG | 0.00050688 | Is there this motif in downstream of splicing site: TCTGG (1 for yes, 0 for no) |
| 6 | DmotifGTAAG | 0.00074236 | Is there this motif in downstream of splicing site: GTAAG (1 for yes, 0 for no) |
| 7 | DmotifGTTTT | 0.00120458 | Is there this motif in downstream of splicing site: GTTTT (1 for yes, 0 for no) |
| 8 | DmotifGTAAT | 0.00055544 | Is there this motif in downstream of splicing site: GTAAT (1 for yes, 0 for no) |
| 9 | DmotifTTCTCT | 0.00052963 | Is there this motif in downstream of splicing site: TTCTCT (1 for yes, 0 for no) |
| 10 | DmotifTATGT | 0.00129761 | Is there this motif in downstream of splicing site: TATGT (1 for yes, 0 for no) |
| 11 | DmotifTTTCTC | 0.00052936 | Is there this motif in downstream of splicing site: TTTCTC (1 for yes, 0 for no) |
| 12 | DmotifCTTTT | 0.00175439 | Is there this motif in downstream of splicing site: CTTTT (1 for yes, 0 for no) |
| 13 | DmotifTTTAG | 0.0006886 | Is there this motif in downstream of splicing site: TTTAG (1 for yes, 0 for no) |
| 14 | DmotifTTTTTC | 0.00077446 | Is there this motif in downstream of splicing site: TTTTTC (1 for yes, 0 for no) |
| 15 | DmotifTCTTG | 0.00056064 | Is there this motif in downstream of splicing site: TCTTG (1 for yes, 0 for no) |
| 16 | DmotifTCTTC | 0.00055469 | Is there this motif in downstream of splicing site: TCTTC (1 for yes, 0 for no) |
| 17 | UmotifTTCTT | 0.00099871 | Is there this motif in upstream of splicing site: TTCTT (1 for yes, 0 for no) |
| 18 | UmotifTCTTT | 0.00168493 | Is there this motif in upstream of splicing site: TCTTT (1 for yes, 0 for no) |
| 19 | UmotifCTCTG | 0.00054215 | Is there this motif in upstream of splicing site: CTCTG (1 for yes, 0 for no) |
| 20 | UmotifGTAAG | 0.00091884 | Is there this motif in upstream of splicing site: GTAAG (1 for yes, 0 for no) |
| 21 | UmotifGTTTT | 0.00129736 | Is there this motif in upstream of splicing site: GTTTT (1 for yes, 0 for no) |
| 22 | UmotifAAATT | 0.00053055 | Is there this motif in upstream of splicing site: AAATT (1 for yes, 0 for no) |
| 23 | UmotifTCTCT | 0.00069679 | Is there this motif in upstream of splicing site: TCTCT (1 for yes, 0 for no) |
| 24 | UmotifCTTTT | 0.00125088 | Is there this motif in upstream of splicing site: CTTTT (1 for yes, 0 for no) |
| 25 | UmotifTTCTC | 0.00050946 | Is there this motif in upstream of splicing site: TTCTC (1 for yes, 0 for no) |
| 26 | allseqGC | 0.00197553 | The GC contant of all sequence |
| 27 | allseqnumberTAA | 0.00093801 | The number of stopdocon TAA in all sequence |
| 28 | allseqnumberTAG | 0.00051914 | The number of stopdocon TAG in all sequence |
| 29 | allseqnumberTGA | 0.00079594 | The number of stopdocon TGA in all sequence |
| 30 | allseqfrequencyATA | 0.00054714 | The frequency of ATA in all sequence |
| 31 | allseqfrequencyAGA | 0.00056293 | The frequency of AGA in all sequence |
| 32 | allseqfrequencyT | 0.00120963 | The frequency of T in all sequence |
| 33 | allseqfrequencyTA | 0.00144838 | The frequency of TA in all sequence |
| 34 | allseqfrequencyTAA | 0.00086404 | The frequency of TAA in all sequence |
| 35 | allseqfrequencyTAT | 0.00055247 | The frequency of TAT in all sequence |
| 36 | allseqfrequencyTT | 0.00068956 | The frequency of TT in all sequence |
| 37 | allseqfrequencyTTA | 0.00065315 | The frequency of TTA in all sequence |
| 38 | allseqfrequencyTTT | 0.00102308 | The frequency of TTT in all sequence |
| 39 | allseqfrequencyCAG | 0.00080081 | The frequency of CAG in all sequence |
| 40 | allseqfrequencyCG | 0.00050537 | The frequency of CG in all sequence |
| 41 | allseqfrequencyG | 0.00064716 | The frequency of G in all sequence |
| 42 | allseqfrequencyGA | 0.00090113 | The frequency of GA in all sequence |
| 43 | allseqfrequencyGTA | 0.00082611 | The frequency of GTA in all sequence |
| 44 | allseqfrequencyGC | 0.00056062 | The frequency of GC in all sequence |
| 45 | allseqfrequencyGGA | 0.00072377 | The frequency of GGA in all sequence |
| 46 | allseqdistr1%A | 0.00050853 | The distribution(position/length) of 1% A in all sequence |
| 47 | allseqdistr25%T | 0.00075153 | The distribution(position/length) of 25% T in all sequence |
| 48 | allseqdistr50%T | 0.00061404 | The distribution(position/length) of 50% T in all sequence |
| 49 | allseqdistr75%T | 0.00062282 | The distribution(position/length) of 75% T in all sequence |
| 50 | allseqdistr25%C | 0.00053424 | The distribution(position/length) of 25% C in all sequence |
| 51 | allseqdistr50%C | 0.00078249 | The distribution(position/length) of 50% C in all sequence |
| 52 | allseqdistr1%G | 0.00053172 | The distribution(position/length) of 1% G in all sequence |
| 53 | allseqdistr25%G | 0.00054524 | The distribution(position/length) of 25% G in all sequence |
| 54 | allseqdistr50%G | 0.00058275 | The distribution(position/length) of 50% G in all sequence |
| 55 | allseqdistr75%G | 0.00100307 | The distribution(position/length) of 75% G in all sequence |
| 56 | allseqdistr100%G | 0.00058178 | The distribution(position/length) of 100% G in all sequence |
| 57 | asseqif%3 | 0.00075603 | Whethere the length of AS region sequence divisible by three |
| 58 | asseqGC | 0.00267662 | The GC contant of AS region sequence |
| 59 | asseqnumberTAA | 0.00154151 | The number of stopdocon TAA in AS region sequence |
| 60 | asseqnumberTAG | 0.00105079 | The number of stopdocon TAG in AS region sequence |
| 61 | asseqnumberTGA | 0.0013909 | The number of stopdocon TGA in AS region sequence |
| 62 | asseqfrequencyA | 0.0013278 | The frequency of A in AS region sequence |
| 63 | asseqfrequencyAA | 0.00189244 | The frequency of AA in AS region sequence |
| 64 | asseqfrequencyAAA | 0.00073439 | The frequency of AAA in AS region sequence |
| 65 | asseqfrequencyAAT | 0.0006585 | The frequency of AAT in AS region sequence |
| 66 | asseqfrequencyAAC | 0.00072175 | The frequency of AAC in AS region sequence |
| 67 | asseqfrequencyAAG | 0.00102058 | The frequency of AAG in AS region sequence |
| 68 | asseqfrequencyAT | 0.00071711 | The frequency of AT in AS region sequence |
| 69 | asseqfrequencyATA | 0.00074491 | The frequency of ATA in AS region sequence |
| 70 | asseqfrequencyATT | 0.00098504 | The frequency of ATT in AS region sequence |
| 71 | asseqfrequencyATC | 0.0007468 | The frequency of ATC in AS region sequence |
| 72 | asseqfrequencyATG | 0.00079964 | The frequency of ATG in AS region sequence |
| 73 | asseqfrequencyAC | 0.00132906 | The frequency of AC in AS region sequence |
| 74 | asseqfrequencyACA | 0.00084261 | The frequency of ACA in AS region sequence |
| 75 | asseqfrequencyACT | 0.00096637 | The frequency of ACT in AS region sequence |
| 76 | asseqfrequencyACG | 0.0005231 | The frequency of ACG in AS region sequence |
| 77 | asseqfrequencyAG | 0.00310413 | The frequency of AG in AS region sequence |
| 78 | asseqfrequencyAGA | 0.00105 | The frequency of AGA in AS region sequence |
| 79 | asseqfrequencyAGT | 0.00071315 | The frequency of AGT in AS region sequence |
| 80 | asseqfrequencyAGC | 0.00061105 | The frequency of AGC in AS region sequence |
| 81 | asseqfrequencyAGG | 0.00095123 | The frequency of AGG in AS region sequence |
| 82 | asseqfrequencyT | 0.0015526 | The frequency of T in AS region sequence |
| 83 | asseqfrequencyTA | 0.00210749 | The frequency of TA in AS region sequence |
| 84 | asseqfrequencyTAA | 0.00279647 | The frequency of TAA in AS region sequence |
| 85 | asseqfrequencyTAT | 0.00092955 | The frequency of TAT in AS region sequence |
| 86 | asseqfrequencyTAC | 0.00071859 | The frequency of TAC in AS region sequence |
| 87 | asseqfrequencyTAG | 0.00100277 | The frequency of TAG in AS region sequence |
| 88 | asseqfrequencyTT | 0.00325989 | The frequency of TT in AS region sequence |
| 89 | asseqfrequencyTTA | 0.00210937 | The frequency of TTA in AS region sequence |
| 90 | asseqfrequencyTTT | 0.00232819 | The frequency of TTT in AS region sequence |
| 91 | asseqfrequencyTTC | 0.00114105 | The frequency of TTC in AS region sequence |
| 92 | asseqfrequencyTTG | 0.00223335 | The frequency of TTG in AS region sequence |
| 93 | asseqfrequencyTC | 0.00106129 | The frequency of TC in AS region sequence |
| 94 | asseqfrequencyTCA | 0.00068795 | The frequency of TCA in AS region sequence |
| 95 | asseqfrequencyTCT | 0.00123544 | The frequency of TCT in AS region sequence |
| 96 | asseqfrequencyTCC | 0.00077003 | The frequency of TCC in AS region sequence |
| 97 | asseqfrequencyTCG | 0.00078591 | The frequency of TCG in AS region sequence |
| 98 | asseqfrequencyTG | 0.00172659 | The frequency of TG in AS region sequence |
| 99 | asseqfrequencyTGA | 0.00067501 | The frequency of TGA in AS region sequence |
| 100 | asseqfrequencyTGT | 0.00217143 | The frequency of TGT in AS region sequence |
| 101 | asseqfrequencyTGC | 0.00131095 | The frequency of TGC in AS region sequence |
| 102 | asseqfrequencyTGG | 0.00078633 | The frequency of TGG in AS region sequence |
| 103 | asseqfrequencyC | 0.00094733 | The frequency of C in AS region sequence |
| 104 | asseqfrequencyCA | 0.00177709 | The frequency of CA in AS region sequence |
| 105 | asseqfrequencyCAA | 0.00201401 | The frequency of CAA in AS region sequence |
| 106 | asseqfrequencyCAT | 0.00066235 | The frequency of CAT in AS region sequence |
| 107 | asseqfrequencyCAG | 0.00379374 | The frequency of CAG in AS region sequence |
| 108 | asseqfrequencyCT | 0.00460841 | The frequency of CT in AS region sequence |
| 109 | asseqfrequencyCTA | 0.00052012 | The frequency of CTA in AS region sequence |
| 110 | asseqfrequencyCTT | 0.00092103 | The frequency of CTT in AS region sequence |
| 111 | asseqfrequencyCTC | 0.00079791 | The frequency of CTC in AS region sequence |
| 112 | asseqfrequencyCTG | 0.00055332 | The frequency of CTG in AS region sequence |
| 113 | asseqfrequencyCC | 0.00059355 | The frequency of CC in AS region sequence |
| 114 | asseqfrequencyCCA | 0.00054019 | The frequency of CCA in AS region sequence |
| 115 | asseqfrequencyCCT | 0.00051827 | The frequency of CCT in AS region sequence |
| 116 | asseqfrequencyCG | 0.00099069 | The frequency of CG in AS region sequence |
| 117 | asseqfrequencyCGA | 0.00087573 | The frequency of CGA in AS region sequence |
| 118 | asseqfrequencyG | 0.0013486 | The frequency of G in AS region sequence |
| 119 | asseqfrequencyGA | 0.00160472 | The frequency of GA in AS region sequence |
| 120 | asseqfrequencyGAA | 0.00073121 | The frequency of GAA in AS region sequence |
| 121 | asseqfrequencyGAT | 0.00075458 | The frequency of GAT in AS region sequence |
| 122 | asseqfrequencyGAC | 0.00051875 | The frequency of GAC in AS region sequence |
| 123 | asseqfrequencyGAG | 0.00086106 | The frequency of GAG in AS region sequence |
| 124 | asseqfrequencyGT | 0.00295507 | The frequency of GT in AS region sequence |
| 125 | asseqfrequencyGTA | 0.00190334 | The frequency of GTA in AS region sequence |
| 126 | asseqfrequencyGTT | 0.00073332 | The frequency of GTT in AS region sequence |
| 127 | asseqfrequencyGTC | 0.00063169 | The frequency of GTC in AS region sequence |
| 128 | asseqfrequencyGTG | 0.00068685 | The frequency of GTG in AS region sequence |
| 129 | asseqfrequencyGC | 0.00111047 | The frequency of GC in AS region sequence |
| 130 | asseqfrequencyGCA | 0.00112206 | The frequency of GCA in AS region sequence |
| 131 | asseqfrequencyGCT | 0.00070271 | The frequency of GCT in AS region sequence |
| 132 | asseqfrequencyGG | 0.00135013 | The frequency of GG in AS region sequence |
| 133 | asseqfrequencyGGA | 0.00124547 | The frequency of GGA in AS region sequence |
| 134 | asseqfrequencyGGT | 0.00070964 | The frequency of GGT in AS region sequence |
| 135 | asseqfrequencyGGC | 0.0006017 | The frequency of GGC in AS region sequence |
| 136 | asseqfrequencyGGG | 0.00068444 | The frequency of GGG in AS region sequence |
| 137 | asseqdistr1%A | 0.00726205 | The distribution(position/length) of 1% A in AS region sequence |
| 138 | asseqdistr25%A | 0.00679882 | The distribution(position/length) of 25% A in AS region sequence |
| 139 | asseqdistr50%A | 0.00161645 | The distribution(position/length) of 50% A in AS region sequence |
| 140 | asseqdistr75%A | 0.00142922 | The distribution(position/length) of 75% A in AS region sequence |
| 141 | asseqdistr100%A | 0.0075252 | The distribution(position/length) of 100% A in AS region sequence |
| 142 | asseqdistr1%T | 0.00601582 | The distribution(position/length) of 1% T in AS region sequence |
| 143 | asseqdistr25%T | 0.0024819 | The distribution(position/length) of 25% T in AS region sequence |
| 144 | asseqdistr50%T | 0.00409853 | The distribution(position/length) of 50% T in AS region sequence |
| 145 | asseqdistr75%T | 0.004453 | The distribution(position/length) of 75% T in AS region sequence |
| 146 | asseqdistr100%T | 0.00978825 | The distribution(position/length) of 100% T in AS region sequence |
| 147 | asseqdistr1%C | 0.00352705 | The distribution(position/length) of 1% C in AS region sequence |
| 148 | asseqdistr25%C | 0.0019445 | The distribution(position/length) of 25% C in AS region sequence |
| 149 | asseqdistr50%C | 0.00090629 | The distribution(position/length) of 50% C in AS region sequence |
| 150 | asseqdistr75%C | 0.00240864 | The distribution(position/length) of 75% C in AS region sequence |
| 151 | asseqdistr100%C | 0.00857147 | The distribution(position/length) of 100% C in AS region sequence |
| 152 | asseqdistr1%G | 0.01593786 | The distribution(position/length) of 1% G in AS region sequence |
| 153 | asseqdistr25%G | 0.01090831 | The distribution(position/length) of 25% G in AS region sequence |
| 154 | asseqdistr50%G | 0.00478994 | The distribution(position/length) of 50% G in AS region sequence |
| 155 | asseqdistr75%G | 0.00422594 | The distribution(position/length) of 75% G in AS region sequence |
| 156 | asseqdistr100%G | 0.00723644 | The distribution(position/length) of 100% G in AS region sequence |
| 157 | asseqdonerGT | 0.00131268 | Is there GT in doner of AS region sequence (1 for yes, 0 for no) |
| 158 | asseqacceptorGT | 0.02884584 | Is there GT in acceptor of AS region sequence (1 for yes, 0 for no) |
| 159 | asseqdonerGC | 0.00222911 | Is there GC in doner of AS region sequence (1 for yes, 0 for no) |
| 160 | asseqacceptorGC | 0.00105832 | Is there GC in acceptor of AS region sequence (1 for yes, 0 for no) |
| 161 | asseqdonerAT | 0.00205132 | Is there AT in doner of AS region sequence (1 for yes, 0 for no) |
| 162 | asseqacceptorAT | 0.00089301 | Is there AT in acceptor of AS region sequence (1 for yes, 0 for no) |
| 163 | asseqdonerAG | 0.0279886 | Is there AG in doner of AS region sequence (1 for yes, 0 for no) |
| 164 | asseqacceptorAG | 0.00086802 | Is there AG in acceptor of AS region sequence (1 for yes, 0 for no) |
| 165 | upfrequencyTC | 0.00054334 | The frequency of TC in upstream sequence |
| 166 | upfrequencyC | 0.00057141 | The frequency of C in upstream sequence |
| 167 | updistr100%G | 0.00087752 | The distribution(position/length) of 100% G in upstream sequence |
| 168 | updonerGT | 0.00056503 | Is there GT in doner of upstream sequence (1 for yes, 0 for no) |
| 169 | updonerAG | 0.0010452 | Is there AG in doner of upstream sequence (1 for yes, 0 for no) |
| 170 | downfrequencyTC | 0.00063755 | The frequency of TC in downstream sequence |
| 171 | downfrequencyTCT | 0.00057412 | The frequency of TCT in downstream sequence |
| 172 | downfrequencyCT | 0.00065043 | The frequency of CT in downstream sequence |
| 173 | downdistr100%G | 0.00058024 | The distribution(position/length) of 100% G in downstream sequence |
| 174 | downdonerAG | 0.00055697 | Is there AG in doner of in downstream sequence (1 for yes, 0 for no) |
| 175 | up30as30if%3 | 0.00526169 | Whethere the length of upstream30 + AS 30bp sequence divisible by three |
| 176 | up30as30GC | 0.00102788 | The GC contant of upstream30AS 30bp + AS 30bp sequence |
| 177 | up30as30numberTAA | 0.00062303 | The number of stopdocon TAA in upstream30AS 30bp + AS 30bp sequence |
| 178 | up30as30frequencyAG | 0.00083881 | The frequency of AG in upstream30AS 30bp + AS 30bp sequence |
| 179 | up30as30frequencyAGG | 0.00063295 | The frequency of AGG in upstream30AS 30bp + AS 30bp sequence |
| 180 | up30as30frequencyTA | 0.00071584 | The frequency of TA in upstream30AS 30bp + AS 30bp sequence |
| 181 | up30as30frequencyTAA | 0.00066461 | The frequency of TAA in upstream30AS 30bp + AS 30bp sequence |
| 182 | up30as30frequencyTTT | 0.00054375 | The frequency of TTT in upstream30AS 30bp + AS 30bp sequence |
| 183 | up30as30frequencyCAG | 0.00129916 | The frequency of CAG in upstream30AS 30bp + AS 30bp sequence |
| 184 | up30as30frequencyG | 0.00057868 | The frequency of G in upstream30AS 30bp + AS 30bp sequence |
| 185 | up30as30frequencyGA | 0.00090161 | The frequency of GA in upstream30AS 30bp + AS 30bp sequence |
| 186 | up30as30frequencyGAT | 0.0005604 | The frequency of GAT in upstream30AS 30bp + AS 30bp sequence |
| 187 | up30as30frequencyGTA | 0.00224494 | The frequency of GTA in upstream30AS 30bp + AS 30bp sequence |
| 188 | up30as30frequencyGGA | 0.00056531 | The frequency of GGA in upstream30AS 30bp + AS 30bp sequence |
| 189 | up30as30frequencyGGT | 0.00241256 | The frequency of GGT in upstream30AS 30bp + AS 30bp sequence |
| 190 | up30as30frequencyGGC | 0.00053686 | The frequency of GGC in upstream30AS 30bp + AS 30bp sequence |
| 191 | up30as30distr100%A | 0.00146039 | The distribution(position/length) of 100% A in upstream30AS 30bp + AS 30bp sequence |
| 192 | up30as30distr50%T | 0.00124887 | The distribution(position/length) of 50% T in upstream30AS 30bp + AS 30bp sequence |
| 193 | up30as30distr75%T | 0.00116163 | The distribution(position/length) of 75% T in upstream30AS 30bp + AS 30bp sequence |
| 194 | up30as30distr100%T | 0.00142157 | The distribution(position/length) of 100% T in upstream30AS 30bp + AS 30bp sequence |
| 195 | up30as30distr50%C | 0.00056688 | The distribution(position/length) of 50% C in upstream30AS 30bp + AS 30bp sequence |
| 196 | up30as30distr75%C | 0.00078279 | The distribution(position/length) of 75% C in upstream30AS 30bp + AS 30bp sequence |
| 197 | up30as30distr25%G | 0.00052417 | The distribution(position/length) of 25% G in upstream30AS 30bp + AS 30bp sequence |
| 198 | up30as30distr50%G | 0.00143519 | The distribution(position/length) of 50% G in upstream30AS 30bp + AS 30bp sequence |
| 199 | up30as30distr75%G | 0.00368218 | The distribution(position/length) of 75% G in upstream30AS 30bp + AS 30bp sequence |
| 200 | up30as30distr100%G | 0.00300135 | The distribution(position/length) of 100% G in upstream30AS 30bp + AS 30bp sequence |
| 201 | up30as30donerGT | 0.00075511 | Is there GT in doner of upstream30AS 30bp + AS 30bp sequence (1 for yes, 0 for no) |
| 202 | up30as30donerGC | 0.00082364 | Is there GC in doner of upstream30AS 30bp + AS 30bp sequence (1 for yes, 0 for no) |
| 203 | up30as30donerAT | 0.00057632 | Is there AT in doner of upstream30AS 30bp + AS 30bp sequence (1 for yes, 0 for no) |
| 204 | up30as30donerAG | 0.00882961 | Is there AG in doner of upstream30AS 30bp + AS 30bp sequence (1 for yes, 0 for no) |
| 205 | up30down30distr50%G | 0.00066765 | The distribution(position/length) of 50% G in upstream 30 + downstream 30 sequence |
| 206 | up30down30distr75%G | 0.00055362 | The distribution(position/length) of 75% G in upstream 30 + downstream 30 sequence |
| 207 | as30down30if%3 | 0.00412092 | Whethere the length of AS 30bp + downstream 30bp sequence divisible by three |
| 208 | as30down30GC | 0.00102468 | The GC contant of AS 30bp + downstream 30bp sequence |
| 209 | as30down30frequencyAAG | 0.00055681 | The frequency of AAG in AS 30bp + downstream 30bp sequence |
| 210 | as30down30frequencyAG | 0.00066375 | The frequency of AG in AS 30bp + downstream 30bp sequence |
| 211 | as30down30frequencyAGG | 0.00057326 | The frequency of AGG in AS 30bp + downstream 30bp sequence |
| 212 | as30down30frequencyT | 0.00060534 | The frequency of T in AS 30bp + downstream 30bp sequence |
| 213 | as30down30frequencyTTT | 0.00064802 | The frequency of TTT in AS 30bp + downstream 30bp sequence |
| 214 | as30down30frequencyTC | 0.00051054 | The frequency of TC in AS 30bp + downstream 30bp sequence |
| 215 | as30down30frequencyTG | 0.00051856 | The frequency of TG in AS 30bp + downstream 30bp sequence |
| 216 | as30down30frequencyC | 0.00052519 | The frequency of C in AS 30bp + downstream 30bp sequence |
| 217 | as30down30frequencyCAG | 0.00161718 | The frequency of CAG in AS 30bp + downstream 30bp sequence |
| 218 | as30down30frequencyGAG | 0.00060058 | The frequency of GAG in AS 30bp + downstream 30bp sequence |
| 219 | as30down30distr1%A | 0.00071615 | The distribution(position/length) of 1% A in AS 30bp + downstream 30bp sequence |
| 220 | as30down30distr1%T | 0.00065785 | The distribution(position/length) of 1% T in AS 30bp + downstream 30bp sequence |
| 221 | as30down30distr25%T | 0.00051918 | The distribution(position/length) of 25% T in AS 30bp + downstream 30bp sequence |
| 222 | as30down30distr50%T | 0.00121401 | The distribution(position/length) of 50% T in AS 30bp + downstream 30bp sequence |
| 223 | as30down30distr75%T | 0.00051052 | The distribution(position/length) of 75% T in AS 30bp + downstream 30bp sequence |
| 224 | as30down30distr1%G | 0.00161531 | The distribution(position/length) of 1% G in AS 30bp + downstream 30bp sequence |
| 225 | as30down30distr25%G | 0.00073252 | The distribution(position/length) of 25% G in AS 30bp + downstream 30bp sequence |
| 226 | as30down30distr50%G | 0.00137949 | The distribution(position/length) of 50% G in AS 30bp + downstream 30bp sequence |
| 227 | as30down30distr75%G | 0.00054911 | The distribution(position/length) of 75% G in AS 30bp + downstream 30bp sequence |
| 228 | as30down30acceptorGT | 0.00237995 | Is there GT in acceptor of AS 30bp + downstream 30bp sequence (1 for yes, 0 for no) |
| 229 | as30down30acceptorAG | 0.00103183 | Is there AG in acceptor of AS 30bp + downstream 30bp sequence (1 for yes, 0 for no) |
| 230 | up50as50if%3 | 0.00313826 | Whethere the length of upstream 50bp + AS 50bp sequence divisible by three |
| 231 | up50as50GC | 0.00248468 | The GC contant of upstream 50bp + AS 50bp sequence |
| 232 | up50as50numberTAA | 0.00090647 | The numberof stopdocon TAA in upstream 50bp + AS 50bp sequence |
| 233 | up50as50frequencyAG | 0.00066188 | The frequency of AG in upstream 50bp + AS 50bp sequence |
| 234 | up50as50frequencyAGG | 0.00052156 | The frequency of AGG in upstream 50bp + AS 50bp sequence |
| 235 | up50as50frequencyT | 0.00055684 | The frequency of T in upstream 50bp + AS 50bp sequence |
| 236 | up50as50frequencyTA | 0.00076884 | The frequency of TA in upstream 50bp + AS 50bp sequence |
| 237 | up50as50frequencyTAA | 0.00107979 | The frequency of TAA in upstream 50bp + AS 50bp sequence |
| 238 | up50as50frequencyTTA | 0.00061682 | The frequency of TTA in upstream 50bp + AS 50bp sequence |
| 239 | up50as50frequencyCAG | 0.00110433 | The frequency of CAG in upstream 50bp + AS 50bp sequence |
| 240 | up50as50frequencyG | 0.00076794 | The frequency of G in upstream 50bp + AS 50bp sequence |
| 241 | up50as50frequencyGA | 0.00070365 | The frequency of GA in upstream 50bp + AS 50bp sequence |
| 242 | up50as50frequencyGAG | 0.00051534 | The frequency of GAG in upstream 50bp + AS 50bp sequence |
| 243 | up50as50frequencyGTA | 0.00156303 | The frequency of GTA in upstream 50bp + AS 50bp sequence |
| 244 | up50as50frequencyGGA | 0.00060143 | The frequency of GGA in upstream 50bp + AS 50bp sequence |
| 245 | up50as50frequencyGGT | 0.00100631 | The frequency of GGT in upstream 50bp + AS 50bp sequence |
| 246 | up50as50distr100%A | 0.0013585 | The distribution(position/length) of 100% A in upstream 50bp + AS 50bp sequence |
| 247 | up50as50distr50%T | 0.00222866 | The distribution(position/length) of 50% T in upstream 50bp + AS 50bp sequence |
| 248 | up50as50distr75%T | 0.00060388 | The distribution(position/length) of 75% T in upstream 50bp + AS 50bp sequence |
| 249 | up50as50distr100%T | 0.00220456 | The distribution(position/length) of 100% T in upstream 50bp + AS 50bp sequence |
| 250 | up50as50distr50%C | 0.00052123 | The distribution(position/length) of 50% C in upstream 50bp + AS 50bp sequence |
| 251 | up50as50distr75%C | 0.00059727 | The distribution(position/length) of 75% C in upstream 50bp + AS 50bp sequence |
| 252 | up50as50distr100%C | 0.00066252 | The distribution(position/length) of 100% C in upstream 50bp + AS 50bp sequence |
| 253 | up50as50distr1%G | 0.0005147 | The distribution(position/length) of 1% G in upstream 50bp + AS 50bp sequence |
| 254 | up50as50distr25%G | 0.00066241 | The distribution(position/length) of 25% G in upstream 50bp + AS 50bp sequence |
| 255 | up50as50distr50%G | 0.00175489 | The distribution(position/length) of 50% G in upstream 50bp + AS 50bp sequence |
| 256 | up50as50distr75%G | 0.00172491 | The distribution(position/length) of 75% G in upstream 50bp + AS 50bp sequence |
| 257 | up50as50distr100%G | 0.00265711 | The distribution(position/length) of 100% G in upstream 50bp + AS 50bp sequence |
| 258 | up50as50donerGT | 0.00080078 | Is there GT in doner of upstream 50bp + AS 50bp sequence (1 for yes, 0 for no) |
| 259 | up50as50donerGC | 0.00072178 | Is there GC in doner of upstream 50bp + AS 50bp sequence (1 for yes, 0 for no) |
| 260 | up50as50donerAT | 0.00075531 | Is there AT in doner of upstream 50bp + AS 50bp sequence (1 for yes, 0 for no) |
| 261 | up50as50donerAG | 0.00973815 | Is there AG in doner of upstream 50bp + AS 50bp sequence (1 for yes, 0 for no) |
| 262 | up50down50distr25%C | 0.00056377 | The distribution(position/length) of 25% C in upstream 50bp + downstream 50bp sequence (1 for yes, 0 for no) |
| 263 | up50down50distr50%C | 0.00064309 | The distribution(position/length) of 50% C in upstream 50bp + downstream 50bp sequence (1 for yes, 0 for no) |
| 264 | up50down50distr50%G | 0.00066223 | The distribution(position/length) of 50% G in upstream 50bp + downstream 50bp sequence (1 for yes, 0 for no) |
| 265 | up50down50distr75%G | 0.00055767 | The distribution(position/length) of 75% G in upstream 50bp + downstream 50bp sequence (1 for yes, 0 for no) |
| 266 | up50down50distr100%G | 0.00054658 | The distribution(position/length) of 100% G in upstream 50bp + downstream 50bp sequence (1 for yes, 0 for no) |
| 267 | as50down50if%3 | 0.00369669 | Whethere the length of AS 50bp + downstream 50bp sequence divisible by three |
| 268 | as50down50GC | 0.00349382 | The GC contant of GC in AS 50bp + downstream 50bp sequence |
| 269 | as50down50numberTAA | 0.0008039 | The number of stopdocon TAA in AS 50bp + downstream 50bp sequence |
| 270 | as50down50frequencyAAG | 0.00053626 | The frequency of AAG in AS 50bp + downstream 50bp sequence |
| 271 | as50down50frequencyAG | 0.000601 | The frequency of AG in AS 50bp + downstream 50bp sequence |
| 272 | as50down50frequencyAGA | 0.00051576 | The frequency of AGA in AS 50bp + downstream 50bp sequence |
| 273 | as50down50frequencyAGG | 0.00060834 | The frequency of AGG in AS 50bp + downstream 50bp sequence |
| 274 | as50down50frequencyT | 0.00096353 | The frequency of T in AS 50bp + downstream 50bp sequence |
| 275 | as50down50frequencyTAA | 0.00052375 | The frequency of TAA in AS 50bp + downstream 50bp sequence |
| 276 | as50down50frequencyTT | 0.00064601 | The frequency of TT in AS 50bp + downstream 50bp sequence |
| 277 | as50down50frequencyTTA | 0.00061379 | The frequency of TTA in AS 50bp + downstream 50bp sequence |
| 278 | as50down50frequencyTC | 0.00060552 | The frequency of TC in AS 50bp + downstream 50bp sequence |
| 279 | as50down50frequencyTCG | 0.0005862 | The frequency of TCG in AS 50bp + downstream 50bp sequence |
| 280 | as50down50frequencyTGT | 0.0007312 | The frequency of TGT in AS 50bp + downstream 50bp sequence |
| 281 | as50down50frequencyC | 0.00053764 | The frequency of C in AS 50bp + downstream 50bp sequence |
| 282 | as50down50frequencyCAG | 0.00073511 | The frequency of CAG in AS 50bp + downstream 50bp sequence |
| 283 | as50down50frequencyCTC | 0.00056771 | The frequency of CTC in AS 50bp + downstream 50bp sequence |
| 284 | as50down50frequencyCG | 0.00056719 | The frequency of CG in AS 50bp + downstream 50bp sequence |
| 285 | as50down50frequencyCGA | 0.00051527 | The frequency of CGA in AS 50bp + downstream 50bp sequence |
| 286 | as50down50frequencyG | 0.00068001 | The frequency of G in AS 50bp + downstream 50bp sequence |
| 287 | as50down50distr1%A | 0.0008114 | The distribution(position/length) of 1% A in AS 50bp + downstream 50bp sequence |
| 288 | as50down50distr1%T | 0.00154536 | The distribution(position/length) of 1% T in AS 50bp + downstream 50bp sequence |
| 289 | as50down50distr50%T | 0.00121784 | The distribution(position/length) of 50% T in AS 50bp + downstream 50bp sequence |
| 290 | as50down50distr1%C | 0.00053517 | The distribution(position/length) of 1% C in AS 50bp + downstream 50bp sequence |
| 291 | as50down50distr1%G | 0.00166606 | The distribution(position/length) of 1% G in AS 50bp + downstream 50bp sequence |
| 292 | as50down50distr25%G | 0.00117026 | The distribution(position/length) of 25% G in AS 50bp + downstream 50bp sequence |
| 293 | as50down50distr50%G | 0.00155005 | The distribution(position/length) of 50% G in AS 50bp + downstream 50bp sequence |
| 294 | as50down50distr75%G | 0.00051549 | The distribution(position/length) of 75% G in AS 50bp + downstream 50bp sequence |
| 295 | as50down50distr100%G | 0.00052391 | The distribution(position/length) of 100% G in AS 50bp + downstream 50bp sequence |
| 296 | as50down50acceptorGT | 0.00348638 | Is there GT in acceptor of AS 50bp + downstream 50bp sequence (1 for yes, 0 for no) |
| 297 | as50down50acceptorAG | 0.00065119 | Is there AG in acceptor of AS 50bp + downstream 50bp sequence (1 for yes, 0 for no) |
